# Red Ginseng-Derived 20(S)-Ginsenoside Rg3 and Compound K Restore Redox Homeostasis and Alleviate LPS-Induced Barrier Damage in Bovine Intestinal Epithelial Cells

**DOI:** 10.3390/antiox15101290

**Published:** 2026-10-04

**Authors:** Haowen Wang, Sungkwon Park, Jialin Zhang, Wen Zhang, Xuanying Xin, Hangshu Xin, Xiangzi Li

**Affiliations:** 1 Engineering Research Center of North-East Cold Region Beef Cattle Science & Technology Innovation, Ministry of Education, Yanbian University, Yanji 133002, China; 2 Department of Animal Science, College of Agriculture, Yanbian University, Yanji 133002, China; 3 Department of Food Science and Biotechnology, Sejong University, Seoul 05006, Republic of Korea; 4 College of Animal Science and Technology, Northeast Agricultural University, Harbin 150030, China

**Keywords:** red ginseng processing by-product, 20(S)-ginsenoside Rg3, compound K, redox homeostasis, lipopolysaccharide, intestinal epithelial injury

## Abstract

This study aimed to determine whether 20(S)-ginsenoside Rg3 (S-Rg3) and compound K (CK), two representative ginsenosides retained in red ginseng processing by-products, could restore redox homeostasis and alleviate lipopolysaccharide (LPS)-induced injury in bovine intestinal epithelial cells, and to clarify the associated autophagy–lysosome mechanisms. Ginsenoside constituents were characterized by LC-MS. Network pharmacology, machine learning-assisted target screening, molecular docking, and molecular dynamics simulations were integrated to predict compound–target interactions. For in vitro validation, bovine small intestinal epithelial cells were pretreated with 8 μM S-Rg3, 8 μM CK, or their combination for 24 h, followed by a 24 h challenge with 50 mg/L LPS. Computationally, S-Rg3 and CK showed favorable predicted binding with Keap1, TLR4, NKIRAS2 p65, and iNOS. Experimentally, S-Rg3, CK, and particularly their combined pretreatment significantly improved cell viability, reduced reactive oxygen species and nitric oxide accumulation, restored antioxidant enzyme activities and Nrf2/HO-1-associated antioxidant defense, suppressed inflammatory activation, and preserved tight junction protein expression compared with the LPS group. The treatments further alleviated LPS-induced autophagy–lysosome dysfunction, endoplasmic reticulum stress, mitochondrial injury, and apoptosis. Multi-omics integration linked these protective effects to inflammatory lipid mediator, arginine/nitric oxide, amino acid, and energy metabolism, together with redox-related signaling pathways. Collectively, S-Rg3 and CK protected bovine intestinal epithelial cells from LPS-induced injury primarily by restoring redox homeostasis and supporting autophagy–lysosome function. These findings provide mechanistic evidence supporting the valorization of red ginseng processing by-products as potential bioactive resources for improving intestinal epithelial resilience in animal production.

## 1. Introduction

The intestinal epithelial barrier is a critical defense system that maintains intestinal homeostasis, supports nutrient absorption, and limits the translocation of pathogenic microorganisms and their toxic products into the internal milieu [1]. In modern ruminant production, intestinal barrier dysfunction is closely associated with diarrhea, impaired growth performance, increased antimicrobial use, and economic losses, particularly in neonatal and weaned calves. During early life and weaning transition, the intestinal epithelium of calves is still undergoing development and adaptation, making it highly susceptible to bacterial toxins, dietary changes, nutritional stress, and environmental challenges. Lipopolysaccharide (LPS), a major component of the outer membrane of Gram-negative bacteria, is an important microbial endotoxin involved in intestinal inflammatory injury. LPS can activate Toll-like receptor-associated signaling and induce inflammatory responses and oxidative stress in intestinal epithelial cells, thereby compromising epithelial function under microbial or nutritional stress [2,3]. Excessive accumulation of reactive oxygen species (ROS) and nitric oxide (NO) disrupts cellular redox homeostasis, damages tight junction structures, impairs epithelial barrier integrity, and promotes cell death. Therefore, bovine intestinal epithelial cell models of LPS-induced injury provide a useful platform for investigating redox-associated epithelial damage and for screening natural bioactive compounds with potential applications in maintaining intestinal health and resilience in livestock production [4].

Redox imbalance is not only a consequence of inflammation but also a key regulatory factor that drives the progression of intestinal epithelial injury and increases the metabolic and immune burden of epithelial cells [5]. Maintenance of cellular redox homeostasis is therefore central to epithelial adaptation to inflammatory and metabolic stress in the intestine. Excessive ROS production induced by LPS can activate inflammatory signaling pathways, such as NF-κB and MAPK, thereby promoting the expression of pro-inflammatory cytokines, including IL-1β and TNF-α, and aggravating epithelial inflammatory injury. Meanwhile, oxidative stress can impair the Nrf2/HO-1 antioxidant defense system, weakening the adaptive capacity of epithelial cells to maintain redox homeostasis under inflammatory stimulation [6,7]. In addition to antioxidant signaling, the autophagy–lysosome system is essential for maintaining epithelial cell quality control and redox homeostasis by removing oxidatively damaged proteins and dysfunctional organelles. When autophagic flux is impaired or lysosomal degradation is disrupted, damaged proteins and organelles cannot be efficiently cleared, which may further amplify ROS accumulation, mitochondrial dysfunction, inflammatory activation, and apoptosis in intestinal epithelial cells [8,9]. Thus, elucidating how natural bioactive compounds alleviate LPS-induced intestinal epithelial injury through the redox homeostasis–autophagy/lysosome axis is of considerable importance for understanding calf diarrhea-related epithelial damage and developing nutritional strategies to support intestinal resilience in young ruminants.

Red ginseng is a steamed and dried form of ginseng that contains various ginsenosides with reported antioxidant, anti-inflammatory, and cytoprotective activities, suggesting its potential value as a natural source of bioactive compounds for intestinal health regulation [10]. During red ginseng processing, considerable amounts of processing by-products are generated, which are often discarded or used in low-value applications [11]. From the perspective of sustainable livestock production and agro-industrial resource utilization, these by-products may represent valuable resources for developing natural functional ingredients. Red ginseng processing by-products may still retain multiple parent ginsenosides as well as low-polarity rare ginsenosides generated during thermal processing and biotransformation, providing a chemical basis for their further nutritional and biological evaluation [12,13]. Compared with parent ginsenosides, some low-polarity ginsenosides generally exhibit better membrane permeability and enhanced biological activity. Therefore, identifying bioactive ginsenoside-related constituents retained in red ginseng processing by-products and clarifying whether they can modulate LPS-induced redox-associated epithelial injury may provide a mechanistic basis for evaluating their potential use in managing calf diarrhea-associated intestinal epithelial damage.

**Figure 1:**
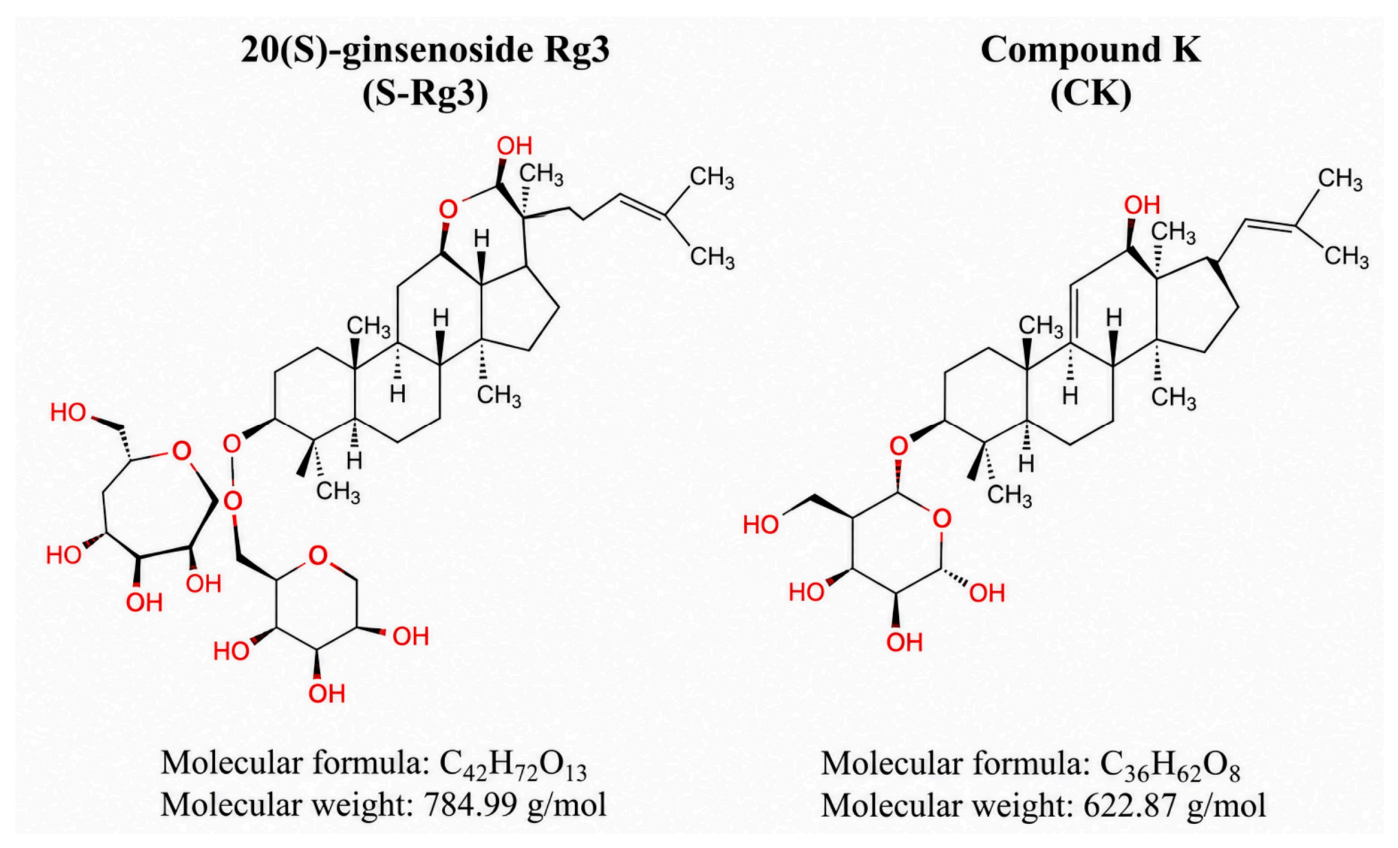
Chemical structures of 20(S)-ginsenoside Rg3 (S-Rg3) and Compound K (CK). The corresponding molecular formulas and molecular weights are shown below each structure.

20(S)-Ginsenoside Rg3 (S-Rg3) and Compound K (CK) are representative ginsenoside-related constituents associated with red ginseng processing and biotransformation. Their chemical structures are shown in Figure 1. Previous studies have linked Rg3 and CK to anti-inflammatory and antioxidant responses, apoptosis regulation, and cellular homeostasis [14,15], but their relevance to bovine intestinal epithelial protection remains insufficiently understood. However, whether S-Rg3 and CK can restore redox homeostasis in bovine intestinal epithelial cells exposed to LPS, and how this redox regulation is coordinated with autophagy–lysosome function, mitochondrial integrity, and apoptosis, remain unclear. Given the chemical complexity of red ginseng processing by-products and the multi-level nature of LPS-induced epithelial injury, an integrated strategy combining constituent identification, network pharmacology, molecular docking, molecular dynamics simulation, cellular validation, transcriptomics, metabolomics, and multi-omics integration is required to clarify the mechanisms of representative bioactive compounds in bovine intestinal epithelial cells.

In this study, LC–MS was used to characterize ginsenoside-related constituents in red ginseng processing by-products, followed by computational screening based on network pharmacology, molecular docking, relative binding energy evaluation, and molecular dynamics simulation to identify representative compounds with potential intestinal protective activity. S-Rg3 and CK were then selected as candidate compounds for validation in an LPS-induced injury model of immortalized bovine small intestinal epithelial cells, which was used as an in vitro model to evaluate epithelial responses relevant to calf diarrhea-associated intestinal injury. Cellular assays, transcriptomics, culture-supernatant-based untargeted metabolomics, and O2PLS-based integrative analysis were used to evaluate redox imbalance, inflammatory responses, epithelial barrier injury, autophagy–lysosome dysfunction, endoplasmic reticulum stress, mitochondrial injury, apoptosis, and extracellular metabolic remodeling. This study aimed to determine whether S-Rg3 and CK could restore redox homeostasis and alleviate LPS-induced injury in bovine intestinal epithelial cells, and to clarify how their redox-protective effects are associated with autophagy–lysosome function, inflammatory signaling, mitochondrial integrity, and apoptosis. The findings may provide mechanistic evidence for the use of red ginseng processing by-products as sources of redox-active bioactive compounds for supporting intestinal epithelial resilience and health in livestock production.

## 2. Materials and Methods

### 2.1. Red Ginseng Processing By-Product Samples and Main Reagents

Red ginseng processing by-product samples were provided by Yanji Kexian Medical Equipment Co., Ltd. (Yanji, China). The samples were collected from by-products generated during red ginseng processing, dried, ground into powder, and stored under dry and light-protected conditions until use. 20(S)-Ginsenoside Rg3 (S-Rg3; catalog no. R-010; CAS no. 14197-60-5; purity > 98%) and ginsenoside Compound K (CK; catalog no. R-028; CAS no. 39262-14-1; purity > 98%) were purchased from Chengdu Ruifensi Biotechnology Co., Ltd. (Chengdu, China). Lipopolysaccharide (LPS) was purchased from Beijing Solarbio Science & Technology Co., Ltd. (Beijing, China). Fetal bovine serum (FBS) and penicillin–streptomycin solution were purchased from Bide Pharmatech Co., Ltd. (Shanghai, China).

### 2.2. LC-MS Analysis of Ginsenosides

Ginsenosides in red ginseng processing by-product samples were analyzed using an ACQUITY UPLC H-Class system coupled with a Xevo G2-XS QTof mass spectrometer (Waters Corporation, Milford, MA, USA). Data acquisition and processing were performed using MassLynx 4.1 software. Chromatographic separation was performed using a Waters HSS T3 column (100 × 2.1 mm, 1.8 μm) maintained at 35 °C, with 0.1% formic acid in water as mobile phase A and 0.1% formic acid in acetonitrile as mobile phase B. The gradient elution was as follows: 0–5 min, 5–10% B; 5–25 min, 10–85% B; 26 min, 5% B; and 26–30 min, 5% B. The injection volume was 2 μL, and the flow rate was 0.2 mL/min.

Mass spectrometric data were acquired in MSE mode over an *m*/*z* range of 100–1500. The cone voltage was 35 V, and the capillary voltage was 3.5 kV in positive ion mode and 3.0 kV in negative ion mode. The desolvation gas flow rate and temperature were 400 L/h and 700 °C, respectively. Leucine enkephalin was used for mass calibration.

Compounds were identified by comparing the retention time, quasi-molecular ions, and major fragment ions of sample peaks with those of authentic ginsenoside standards. Compounds marked with an asterisk (*) in Table 1 were confirmed in the red ginseng processing by-product samples by comparison with authentic standards. Information on the reference standards is provided in Table S1.

### 2.3. Network Pharmacology Analysis

Network pharmacology analysis was performed to identify potential targets and pathways through which red ginseng candidate compounds may regulate calf diarrhea-related intestinal inflammatory injury. Chemical constituents of red ginseng were retrieved from the TCMSP database, and candidate active compounds were screened based on oral bioavailability, drug-likeness, and Caco-2 permeability. Potential targets of the screened compounds were predicted using SwissTargetPrediction and PharmMapper. Disease-related targets were collected from GeneCards and DisGeNET using keywords related to neonatal calf diarrhea. Overlapping targets between compound-related and disease-related targets were used to construct the compound–target–disease network and protein–protein interaction (PPI) network. Hub targets were screened based on topological parameters, followed by Gene Ontology (GO) and Kyoto Encyclopedia of Genes and Genomes (KEGG) enrichment analyses.

To further prioritize the hub targets, the public transcriptomic dataset GSE223548 was downloaded from the GEO database for machine-learning analysis. This dataset contains RNA-seq profiles of HCT-8 intestinal epithelial-like cells infected with bovine coronavirus and/or Cryptosporidium parvum, representing a pathogen-induced intestinal epithelial injury model associated with calf diarrhea. Hub genes identified from the PPI network were mapped to the expression matrix of GSE223548, and shared genes were used as candidate features. Multiple machine-learning algorithms, including random forest, support vector machine, XGBoost, generalized linear model, LASSO, and C5.0-based feature selection, were applied to identify key candidate genes. Model performance was evaluated using ROC curves, residual analysis, random forest error curves, and cross-validation accuracy. Detailed database information, screening criteria, and analytical parameters are provided in Supplementary Methods S1.

### 2.4. Molecular Docking and Relative Binding Energy Calculation

Based on the LC–MS results and network pharmacology analysis, representative ginsenosides detected in red ginseng processing by-products were selected for molecular docking. The three-dimensional structures of the candidate compounds were obtained from the PubChem database. The receptor structures used for molecular docking included Keap1 (PDB ID: 9VEQ), TLR4 (PDB ID: 2Z64), and iNOS (PDB ID: 4CX7), which were obtained from the RCSB Protein Data Bank. For NKIRAS2, the AlphaFold-predicted structure AF-Q9NYR9-F1, corresponding to UniProt accession Q9NYR9, was used.

Blind molecular docking was performed using AutoDock Vina version 1.2.7. Prior to docking, receptor structures were prepared by removing water molecules and non-essential heteroatoms, followed by hydrogen addition and charge assignment, while ligand structures were prepared and converted into PDBQT format. For each receptor, the docking grid was defined to encompass the entire receptor structure rather than a predefined binding pocket, allowing an unbiased search of potential ligand-binding regions across the protein surface. An independent docking box was defined for each receptor according to its spatial dimensions. Docking poses were ranked according to the predicted binding energies, and the pose with the lowest predicted binding energy was selected as the representative binding conformation for subsequent analysis. Protein–ligand interactions were analyzed and visualized using BIOVIA Discovery Studio Visualizer 2021, and two-dimensional interaction diagrams were generated using the “Show 2D Diagram” function.

Docking results were expressed as binding energies. Considering the large differences in molecular weight among ginsenosides, relative binding energy was calculated as an auxiliary screening index using the following formula:

(1) $$\mathrm{Relative binding energy}\;=\;\frac{\mathrm{binding energy}}{\mathrm{relative molecular mass}}$$

Candidate compounds were selected for subsequent analysis by integrating docking binding energy, relative binding energy, and LC-MS detection results. Although Compound K (CK) was not included among the seven red ginseng candidate compounds obtained from the initial network pharmacology screening, it was detected in red ginseng processing by-products by LC-MS and ranked highly in the relative binding energy evaluation. Therefore, CK was included as a supplementary candidate compound and compared with S-Rg3 in the molecular docking analysis of key targets. Detailed procedures for ligand/receptor preparation, docking parameter setting, and interaction visualization are provided in Supplementary Methods S2.

### 2.5. Molecular Dynamics Simulation

To further evaluate the dynamic stability of the ligand-receptor complexes obtained from molecular docking, all-atom molecular dynamics (MD) simulations were performed using AMBER 24. Each complex was solvated, neutralized with Na^+^ and Cl^−^ ions, and subjected to energy minimization, heating, equilibration, and 100 ns production simulation. The simulations were performed at 300 K and 1 bar.

Trajectory analyses were conducted using the CPPTRAJ module, including root mean square deviation (RMSD), root mean square fluctuation (RMSF), radius of gyration (Rg), solvent accessible surface area (SASA), and hydrogen bond analysis. Binding free energies were calculated using the molecular mechanics Poisson–Boltzmann surface area (MM-PBSA) method. Detailed simulation parameters are provided in Supplementary Methods S3.

### 2.6. Cell Culture and Treatment

Immortalized bovine small intestinal epithelial cells were purchased from Zhejiang Meisen Cell Technology Co., Ltd. (Jinhua, China; catalog no. CTCC-001-0886). According to the supplier’s certificate of analysis, the cell line had been authenticated and quality-controlled before use and was negative for mycoplasma contamination. Cells were cultured in complete medium containing 10% fetal bovine serum and 1% penicillin–streptomycin solution at 37 °C in a humidified incubator with 5% CO_2_. Cells from passages 10–15 were used for all experiments.

S-Rg3 and CK stock solutions were prepared at 20 mM in DMSO. At the working concentration of 8 μM, each compound contributed approximately 0.04% (*v*/*v*) DMSO to the culture medium, and the maximum final DMSO concentration in the combined S-Rg3 + CK treatment was therefore 0.08% (*v*/*v*). The Control group received an equivalent vehicle treatment, and the final DMSO concentration did not exceed 0.08% (*v*/*v*) in any experimental group. Based on preliminary concentration- and time-response screening experiments, 8 μM S-Rg3, 8 μM CK, and 50 mg/L LPS were selected for subsequent treatments. The LPS concentration was selected on the basis of the injury response observed in this bovine small intestinal epithelial cell model, together with previous reports showing variable LPS responsiveness and the use of relatively high LPS concentrations in intestinal epithelial injury models [16,17]. Cells were divided into seven groups: Control, LPS, S-Rg3 + LPS, CK + LPS, S-Rg3 + CK + LPS, S-Rg3, and CK.

Cells in the S-Rg3 + LPS, CK + LPS, and S-Rg3 + CK + LPS groups were pretreated with 8 μM S-Rg3, 8 μM CK, or their combination, respectively, for 24 h, followed by stimulation with 50 mg/L LPS for an additional 24 h. The S-Rg3 and CK alone groups were treated with 8 μM S-Rg3 or CK for 24 h, respectively. After treatment, cells were collected for cellular validation assays and transcriptomic analysis, whereas culture supernatants were collected for untargeted metabolomic analysis.

In addition, a fixed-ratio concentration–response CCK-8 assay was performed to evaluate the effect of combined S-Rg3 and CK treatment on cell viability. S-Rg3 and CK were combined at a 1:1 ratio at concentrations of 1 + 1, 2 + 2, 4 + 4, 8 + 8, and 16 + 16 μM. Bliss interaction analysis was further performed to characterize the combined protective effects of S-Rg3 and CK under LPS-induced injury conditions, as described in Supplementary Methods S4.

### 2.7. Cell Viability Assay

Cell viability under different treatment conditions was assessed using the CCK-8 assay. The Cell Counting Kit-8 (CCK-8; catalog no. K1018) was purchased from APExBIO Technology LLC (Houston, TX, USA). Cells were seeded into 96-well plates and treated according to the experimental design after attachment and growth to an appropriate status.

To determine the LPS modeling conditions, cells were treated with different concentrations of LPS for 6, 12, and 24 h. To evaluate the effects of S-Rg3 and CK on cell viability, cells were treated with different concentrations of S-Rg3 or CK for 24 h. Based on the screening results, cells were further pretreated with different concentrations of S-Rg3 or CK for 24 h, followed by treatment with 50 mg/L LPS for another 24 h, to assess the effects of S-Rg3 and CK on LPS-induced changes in cell viability.

After treatment, CCK-8 working solution was added to each well, and the cells were incubated in the dark at 37 °C in a humidified incubator with 5% CO_2_. The absorbance was then measured at 450 nm using a microplate reader. Cell viability was calculated using the following formula:

(2) $$\mathrm{Cell viability}\left(\%\right)=\frac{A_{\mathrm{treatment}}-A_{\mathrm{blank}}}{A_{\mathrm{control}}-A_{\mathrm{blank}}}\times 100\%$$

where *A*_treatment_ represents the absorbance of the treatment group, *A*_control_ represents the absorbance of the control group, and *A*_blank_ represents the absorbance of the blank wells. Each group included at least three technical replicates, and the experiment was repeated at least three times.

### 2.8. Measurement of Oxidative Stress-Related Indicators

Intracellular ROS levels were detected using a DCFH-DA ROS assay kit (Beyotime Biotechnology, Shanghai, China). After treatment, cells were washed with PBS and incubated with diluted DCFH-DA working solution at 37 °C in the dark. After washing to remove excess probe, fluorescence images were captured using a fluorescence microscope, and green fluorescence intensity was analyzed using Fiji (ImageJ version 1.54k; National Institutes of Health, Bethesda, MD, USA). CAT activity, SOD activity, and MDA content were measured using commercial assay kits (Beijing Solarbio Science & Technology Co., Ltd., Beijing, China) according to the manufacturer’s instructions. After treatment, cells were collected and lysed, and the supernatants were used for the assays. Absorbance was measured at the corresponding wavelengths using a microplate reader. CAT and SOD activities were expressed as U/mg protein, and MDA content was expressed as nmol/mg protein. All experiments were repeated at least three times.

### 2.9. NO Fluorescence Detection

Intracellular NO levels were detected using an NO fluorescent probe assay kit (Beyotime Biotechnology, China). After treatment, cells were washed gently with PBS and incubated with the NO fluorescent probe working solution prepared according to the manufacturer’s instructions at 37 °C in the dark. After incubation, cells were washed with PBS to remove excess probe, and an appropriate volume of PBS was added to keep the cells moist.

Fluorescence images were captured using a fluorescence microscope. Green fluorescence signals were used to reflect intracellular NO levels, and the mean fluorescence intensity was analyzed using Fiji ImageJ software.

### 2.10. Western Blot

After treatment, cells were washed with pre-cooled PBS and lysed with RIPA lysis buffer (Beijing Solarbio Science & Technology Co., Ltd., Beijing, China) to extract total protein. The lysates were centrifuged, and the supernatants were collected for protein concentration determination using a BCA protein assay kit (Shanghai EpiZyme Biomedical Technology Co., Ltd., Shanghai, China). Equal amounts of protein were mixed with loading buffer, boiled for denaturation, and separated by SDS-PAGE. Protein gels were prepared using a gel preparation kit from Yeasen Biotechnology (Shanghai) Co., Ltd. (Shanghai, China).

After electrophoresis, proteins were transferred onto PVDF membranes. The membranes were blocked with 5% skim milk or BSA at room temperature and then incubated with primary antibodies overnight at 4 °C. The next day, the membranes were washed with TBST (Beijing Solarbio Science & Technology Co., Ltd., Beijing, China) and incubated with the corresponding HRP-conjugated secondary antibodies at room temperature. After washing, protein bands were visualized using an ECL chemiluminescence reagent (New Cell & Molecular Biotech Co., Ltd., Suzhou, China) and captured using a gel imaging system. Band intensities were quantified using Fiji (ImageJ version 1.54k; National Institutes of Health, Bethesda, MD, USA). GAPDH was used as the internal reference protein. The antibodies and their dilution ratios are listed in Table S2.

### 2.11. Immunofluorescence Staining

After treatment, cells were washed gently with PBS three times and fixed with 4% paraformaldehyde (Wuhan Servicebio Technology Co., Ltd., Wuhan, China) for 15–20 min. After washing with PBS, cells were permeabilized with 0.1% Triton X-100 (Beijing Labgic Technology Co., Ltd., Beijing, China) at room temperature for 10–15 min when intracellular protein staining was required. Cells were then washed again with PBS and blocked with 5% BSA or normal blocking serum at room temperature for 30–60 min to reduce nonspecific binding.

After blocking, cells were incubated with the corresponding primary antibodies overnight at 4 °C. The next day, cells were washed with PBS three times and incubated with fluorescence-conjugated secondary antibodies at room temperature in the dark for 1 h. After washing, nuclei were counterstained with Hoechst staining solution (Solarbio Science & Technology Co., Ltd., China) for 5–10 min in the dark. The cells were then washed with PBS and mounted with antifade mounting medium.

Images were captured using an Olympus IX73 inverted fluorescence microscope (Olympus Corporation, Tokyo, Japan) or a Multi-SIM imaging system (NanoInsights-Tech Co., Ltd., Beijing, China), as required. The primary and secondary antibodies and their dilution ratios are listed in Table S2. Representative images were used to show changes in the expression and distribution of target proteins among different treatment groups.

### 2.12. Transmission Electron Microscopy

Transmission electron microscopy (TEM) was used to observe ultrastructural changes in bovine small intestinal epithelial cells. After treatment, cells were collected and fixed with 2.5% glutaraldehyde at 4 °C. The samples were then post-fixed with osmium tetroxide, dehydrated through a graded ethanol series, embedded in resin, and cut into ultrathin sections. The ultrathin sections were double-stained with uranyl acetate and lead citrate, and representative images were acquired using a transmission electron microscope (HT7800, Hitachi High-Tech Corporation, Tokyo, Japan). The detailed procedures were performed according to previously described methods with minor modifications [18]. Mitochondrial morphology and organelle integrity were mainly evaluated.

### 2.13. Lysosomal and Mitochondrial Staining, CTSB Detection, and Cell Death/Apoptosis Analysis

After treatment, lysosomes and mitochondria were stained using LysoTracker and mitochondrial fluorescent probes, respectively, both purchased from MedChemExpress LLC (Monmouth Junction, NJ, USA). Cells were incubated with the corresponding working solutions at 37 °C in the dark according to the manufacturer’s instructions, washed with PBS, and imaged using a fluorescence microscope.

Intracellular Cathepsin B (CTSB) levels were measured using a CTSB ELISA kit (Xiamen Huijia Biotechnology Co., Ltd., Xiamen, China) according to the manufacturer’s instructions. Absorbance was measured using a microplate reader, and CTSB concentrations were calculated from the standard curve.

Mitochondrial membrane potential was assessed using JC-1 staining, and PI staining was used to evaluate cell membrane integrity. Cell apoptosis was detected using a TUNEL flow cytometry assay kit. The JC-1, PI, and TUNEL kits were purchased from Beyotime Biotechnology (China), and all procedures were performed according to the manufacturer’s instructions. TUNEL-positive cells were quantified by flow cytometry.

### 2.14. Pharmacological Inhibition of Autophagy–Lysosomal Function

To determine whether autophagy–lysosomal restoration functionally contributes to the protective effects of S-Rg3 and CK, pharmacological inhibition experiments were performed using bafilomycin A1 (BafA1; MedChemExpress, MCE, USA), an inhibitor of lysosomal acidification and autophagic degradation. Before the formal inhibition experiment, the working concentration of BafA1 was determined by preliminary concentration-response experiments. Cells were treated with 0, 25, 50, 75, or 100 nM BafA1 for 6 h, and cell viability was assessed using the CCK-8 assay. To further evaluate the inhibitory effect of BafA1 on autophagic degradation, cells were treated with 0, 50, 75, or 100 nM BafA1 for 6 h, followed by Western blot analysis of LC3-II and p62 protein expression. Protein levels were normalized to GAPDH. Based on these preliminary experiments, 75 nM BafA1 was selected as the working concentration because it was the lowest concentration that induced clear accumulation of LC3-II and p62 without significantly affecting cell viability. For the formal inhibition experiment, cells were divided into five groups: Control, LPS, S-Rg3 + CK + LPS, LPS + BafA1, and S-Rg3 + CK + LPS + BafA1. Cells in the S-Rg3 + CK-containing groups were pretreated with 8 μM S-Rg3 and 8 μM CK for 24 h, followed by stimulation with 50 mg/L LPS for an additional 24 h. In the BafA1-treated groups, 75 nM BafA1 was added during the final 6 h of LPS exposure. Cells from all groups were collected at the same experimental endpoint. After treatment, autophagy–lysosomal status was evaluated by Western blot analysis of LC3-II, p62, LAMP1, and CTSB, and epithelial barrier integrity was further assessed by ZO-1 protein expression. These experiments were used to determine whether pharmacological disruption of autophagy–lysosomal function attenuated the protective effects of combined S-Rg3 and CK treatment under LPS-induced inflammatory injury conditions.

### 2.15. RNA Sequencing and Transcriptomic Data Analysis

After the corresponding treatments, cells from the Control, LPS, S-Rg3 + LPS, CK + LPS, and S-Rg3 + CK + LPS groups were collected for RNA sequencing, with six biological replicates per group (*n* = 6). Transcriptome sequencing was performed by Shanghai Personal Biotechnology Co., Ltd. (Shanghai, China).

After total RNA extraction, RNA concentration, purity, and integrity were assessed, and qualified samples were used for library construction. Transcriptome libraries were then prepared and sequenced using an Illumina high-throughput sequencing platform. Raw sequencing data were subjected to quality control to remove adaptor sequences, low-quality reads, and reads with a high proportion of ambiguous bases, generating clean reads for downstream analysis.

Clean reads were mapped to the bovine reference genome, followed by gene expression quantification. Differentially expressed genes (DEGs) were identified using the criteria of |log2(FoldChange)| > 1 and adjusted *p* value < 0.05. The main comparisons included Control vs. LPS, LPS vs. S-Rg3 + LPS, LPS vs. CK + LPS, and LPS vs. S-Rg3 + CK + LPS.

Principal component analysis (PCA) was performed based on the gene expression matrix to evaluate overall sample distribution and between-group differences. Differentially expressed genes (DEGs) were further subjected to GO functional enrichment analysis, KEGG and gene set enrichment analysis (GSEA). The PPI network was constructed using the STRING database and visualized using Cytoscape software (version 3.9.1; Cytoscape Consortium). Key candidate genes were further screened from the DEG results using LASSO regression, and additional machine-learning algorithms were applied as exploratory analyses to evaluate the consistency and relative importance of the selected candidate genes.

### 2.16. Untargeted Metabolomics and Data Analysis

Untargeted metabolomic analysis was performed using culture supernatants from the same experimental groups as those used for transcriptomic analysis, including Control, LPS, S-Rg3 + LPS, CK + LPS, and S-Rg3 + CK + LPS groups, with six biological replicates per group (n = 6). The analysis was conducted by Personal Biotechnology Co., Ltd. (China).

After treatment, culture supernatants were collected, centrifuged to remove cell debris, and subjected to metabolite extraction. The extracted samples were analyzed using an ultra-performance liquid chromatography-tandem mass spectrometry (UPLC-MS/MS) platform in both positive and negative ionization modes. Raw data were processed for peak detection, alignment, retention time correction, peak area integration, normalization, and filtering.

Differentially expressed metabolites (DEMs) were identified using the criteria of fold change ≥ 1.2 or ≤1/1.2, VIP ≥ 1, and FDR-adjusted *p* value < 0.05. The main comparisons included Control vs. LPS, LPS vs. S-Rg3 + LPS, LPS vs. CK + LPS, and LPS vs. S-Rg3 + CK + LPS. DEMs were further used for PCA, clustering analysis, and KEGG pathway enrichment analysis.

### 2.17. Integrated Transcriptomic and Metabolomic Analysis

To further investigate the integrated regulatory effects of S-Rg3, CK, and their combined pretreatment on LPS-induced cellular injury, transcriptomic and untargeted metabolomic data were jointly analyzed. The metabolomic data were obtained from culture supernatants corresponding to the same samples used for transcriptomic analysis, including the Control, LPS, S-Rg3 + LPS, CK + LPS, and S-Rg3 + CK + LPS groups.

Differentially expressed genes and differentially expressed metabolites were first identified for each comparison and annotated using the KEGG database. Two-way orthogonal partial least squares (O2PLS) analysis was then performed to model the integrated variation between transcriptomic and metabolomic datasets and to explore the overall covariation between gene expression changes and metabolite abundance changes. O2PLS joint loading analysis was used to identify genes and metabolites with high contributions to the integrated transcriptomic-metabolomic variation.

In addition, KEGG pathways commonly enriched by differentially expressed genes and metabolites were identified. Pathway-gene-metabolite association networks were further constructed to visualize the relationships among key pathways, genes, and metabolites. By integrating O2PLS joint loading results, shared KEGG enrichment analysis, and pathway-gene-metabolite networks, the potential transcriptomic-metabolomic mechanisms underlying the effects of S-Rg3, CK, and their combined pretreatment on LPS-induced cellular injury were analyzed.

### 2.18. Statistical Analysis

All statistical analyses and graph generation were performed using GraphPad Prism software 11.1.0. Data are presented as mean ± SEM unless otherwise stated. Before statistical analysis, data were assessed for normality using the Shapiro–Wilk test and for homogeneity of variance using the Brown–Forsythe test. For comparisons among multiple groups, one-way analysis of variance (ANOVA) followed by Tukey’s multiple comparison test was used when the data met the assumptions of normality and homogeneity of variance. When the assumption of homogeneity of variance was not met, Welch’s ANOVA followed by Games–Howell’s multiple comparison test was applied. For comparisons between two groups, Student’s *t*-test was used for normally distributed data with equal variances; otherwise, Welch’s *t*-test or the Mann–Whitney U test was used as appropriate.

For the fixed-ratio combination assay, CCK-8-derived Fa values were calculated based on cell viability recovery relative to the Control and LPS groups. The effects of treatment, concentration, and their interaction on Fa values were analyzed using two-way ANOVA followed by Tukey’s multiple comparisons test. Bliss independence analysis was performed as a descriptive interaction analysis. The Bliss-expected effect was calculated as EBliss = ES-Rg3 + ECK − ES-Rg3 × ECK, where ES-Rg3 and ECK represent the Fa values of S-Rg3 and CK single treatments at the corresponding concentrations, respectively. Bliss excess was calculated as Eobserved − EBliss, where Eobserved represents the Fa value of the combined treatment. A positive Bliss excess value was interpreted as an observed combined effect higher than the Bliss-expected additive effect.

A value of *p* < 0.05 was considered statistically significant. In the figures, significant differences between two groups are indicated as * *p* < 0.05, ** *p* < 0.01, and *** *p* < 0.001. For multiple-group comparisons, different lowercase letters indicate significant differences among groups (*p* < 0.05). For the fixed-ratio combination assay, * and # were used to indicate significant differences between the combined treatment and the corresponding single treatments, as specified in the figure legend.

## 3. Results

### 3.1. LC-MS Analysis of Ginsenosides in Red Ginseng Processing By-Products

**Figure 2:**
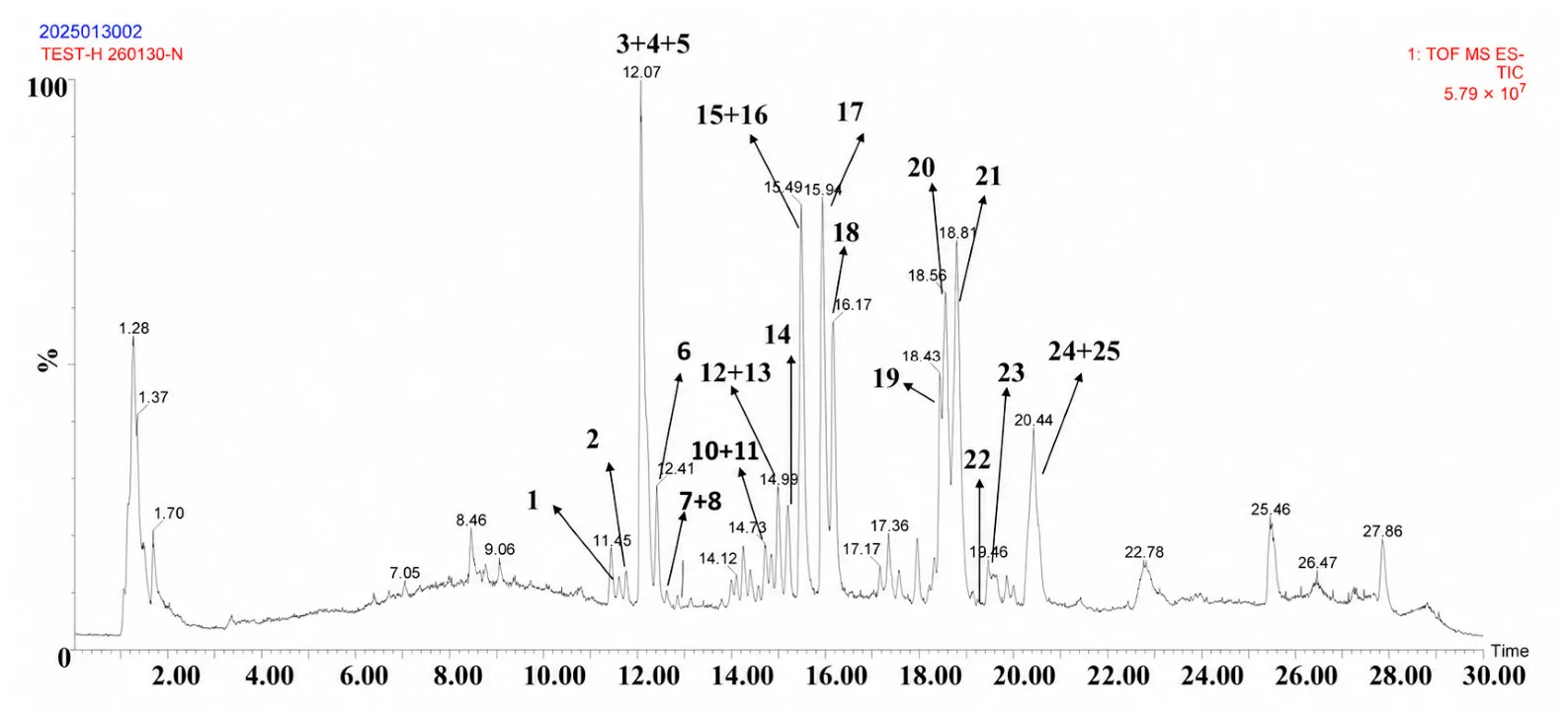
LC-MS profiling of red ginseng processing by-products. TIC acquired in negative ion mode. Labeled peak numbers correspond to the ginsenoside-related candidate peaks listed in Table 1. Asterisks in Table 1 indicate compounds confirmed using authentic standards.

Red ginseng processing by-product samples were analyzed using a Waters G2-XS QTof LC-MS system, and the total ion chromatogram was obtained. As shown in Figure 2, multiple characteristic chromatographic peaks corresponding to ginsenoside-related constituents were observed, mainly distributed between 11 and 21 min, indicating that red ginseng processing by-products still retained multiple ginsenoside-related compounds.

The major chromatographic peaks were further identified by comparison with authentic ginsenoside standards based on retention time, quasi-molecular ions, and major fragment ions. As shown in Table 1, a total of 25 ginsenoside-related candidate peaks were matched or tentatively assigned, among which compounds marked with an asterisk (*) were confirmed in the red ginseng processing by-product samples. These confirmed compounds included ginsenoside Rg1, Rf, Rg2, 20(S/R)-Rh1, Rd, F1, F2, 20(S/R)-Rg3, Compound K, Rk1, Rg5, and 20(S/R)-Rh2. Notably, the samples contained not only prototype ginsenosides such as Rg1, Rf, and Rd, but also low-polarity or processing-related transformed ginsenosides, including Rg3, Rh2, Rk1, Rg5, and Compound K. These findings indicated that red ginseng processing by-products retained several bioactive ginsenoside-related constituents, supporting their further screening and functional validation.

**Table 1:** LC–QTOF–MS characterization of ginsenosides identified in red ginseng processing by-product samples.

Peak	RT (min)	Formula	*m*/*z* Calcd/Obs	Adduct	Major Framents (*m*/*z*)	Mass Eror (ppm)	Identification
1	11.44	C_42_H_72_O_14_	845.4904/845.4863	[M + COOH]^−^	799.4832, 475.7961	−4.85	Ginsenoside Rg1 *
2	11.64	C_42_H_72_O_14_	845.4904/845.4852	[M + COOH]^−^	799.4862	−6.15	Ginsenoside Rf *
3	11.96	C_42_H_72_O_13_	829.4955/829.4929	[M + COOH]^−^	783.4991, 475.3843	−3.13	Ginsenoside Rg2 *
4	12.07	C_48_H_76_O_19_	955.4908/955.4935	[M-H]^−^	793.4901	2.83	Ginsenoside Ro
5	12.17	C_36_H_62_O_9_	683.4376/683.4382	[M + COOH]^−^	637.4362, 435.2494	0.88	20(S)-Ginsenoside Rh1 *
6	12.41	C_36_H_62_O_9_	683.4376/683.4380	[M + COOH]^−^	637.4311	0.59	20(R)-Ginsenoside Rh1 *
7	12.64	C_48_H_82_O_18_	945.5428/945.5326	[M-H]^−^	783.4857	−10.79	Ginsenoside Rd *
8	12.663	C_42_H_72_O_13_	829.4955/829.4936	[M + COOH]^−^	783.4903	−2.29	20(R)-Ginsenoside Rg2
9	12.91	C_36_H_62_O_9_	683.4376/683.4357	[M + COOH]^−^	637.4282	−2.78	Ginsenoside F1 *
10	14.72	C_42_H_72_O_13_	829.4955/829.4905	[M + COOH]^−^	783.4847	−6.03	Ginsenoside F2 *
11	14.73	C_42_H_70_O_12_	811.4849/811.4816	[M + COOH]^−^	765.4789	−4.07	Ginsenoside F4
12	15.00	C_58_H_98_O_26_	1209.6274/1209.6139	[M-H]^−^	793.4398	−11.16	Ginsenoside Ra2
13	15.00	C_42_H_70_O_12_	811.4849/811.4838	[M + COOH]^−^	765.4789, 751.4629	−1.36	Ginsenoside Rg6
14	15.21	C_36_H_60_O_8_	665.4270/665.4281	[M + COOH]^−^	619.4213	1.65	Ginsenoside Rk3
15	15.47	C_55_H_92_O_23_	1119.5957/1119.6122	[M-H]^−^	954.0165	14.74	Ginsenoside Rs2
16	15.51	C_36_H_60_O_8_	665.4270/665.4268	[M + COOH]^−^	619.4219	−0.30	Ginsenoside Rh4
17	15.91	C_42_H_72_O_13_	829.4955/829.5062	[M + COOH]^−^	783.4973	12.90	20(S)-Ginsenoside Rg3 *
18	16.16	C_42_H_72_O_13_	829.4955/829.4980	[M + COOH]^−^	783.4912	3.01	20(R)-Ginsenoside Rg3 *
19	18.47	C_36_H_62_O_8_	667.4427/667.4407	[M + COOH]^−^	621.4352	−3.00	Compound K *
20	18.57	C_42_H_70_O_12_	811.4849/811.4925	[M + COOH]^−^	765.4839	9.37	Ginsenoside Rk1 *
21	18.81	C_42_H_70_O_12_	811.4849/811.4913	[M + COOH]^−^	765.4833	7.89	Ginsenoside Rg5 *
22	19.27	C_36_H_62_O_8_	667.4427/667.4413	[M + COOH]^−^	621.4309	−2.10	20(S)-Ginsenoside Rh2 *
23	19.43	C_36_H_62_O_8_	667.4427/667.4414	[M + COOH]^−^	621.4317	−1.95	20(R)-Ginsenoside Rh2 *
24	20.29	C_44_H_72_O_13_	807.4900/807.4860	[M-H]^−^	765.4794	−4.95	Ginsenoside Rg5 isomer 1
25	20.57	C_44_H_72_O_13_	807.4900/807.4874	[M-H]^−^	765.4771	−3.22	Ginsenoside Rg5 isomer 2

* Compounds marked with an asterisk (*) were confirmed in red ginseng processing by-products by comparison with reference standards based on retention time, quasi-molecular ions, and major fragment ions; unmarked compounds were tentatively assigned as ginsenoside-related candidate peaks. All reference standards were purchased from Chengdu Ruifensi Biotechnology Co., Ltd. (Chengdu, China). Compounds annotated as isomers were assigned based on similar quasi-molecular ions and fragment ions but different retention times. RT, retention time; calcd/obs, calculated/observed; RS, reference standard. The 20(S)/20(R) designation refers specifically to the stereochemical configuration at C-20. These pairs are C-20 epimers (diastereomers), rather than enantiomers.

### 3.2. Virtual Screening and Molecular Docking Analysis of Candidate Bioactive Compounds in Red Ginseng Processing By-Products

Based on the LC-MS profiling of red ginseng processing by-products, the detected ginsenosides were selected as candidate compounds for virtual screening and molecular docking analysis against inflammation- and oxidative stress-related targets. The potential binding ability of each candidate compound to the target proteins was preliminarily evaluated by comparing docking binding energies.

As shown in Figure S1A, the predicted binding energies varied among the different ginsenosides and target proteins, including Keap1, TLR4, NKIRAS2, and iNOS. Several compounds exhibited relatively favorable binding energies toward these targets. Because the candidate ginsenosides differed substantially in molecular mass, relative binding energy was further calculated as an auxiliary index to complement the interpretation of the original docking scores. As shown in Figure S1B, normalization by molecular mass altered the relative ranking of several candidate compounds compared with the original binding-energy results. By integrating the original docking energies, relative binding-energy values, and LC–MS detection results, S-Rg3 and CK were selected as representative candidate molecules for subsequent analyses.

**Figure 3:**
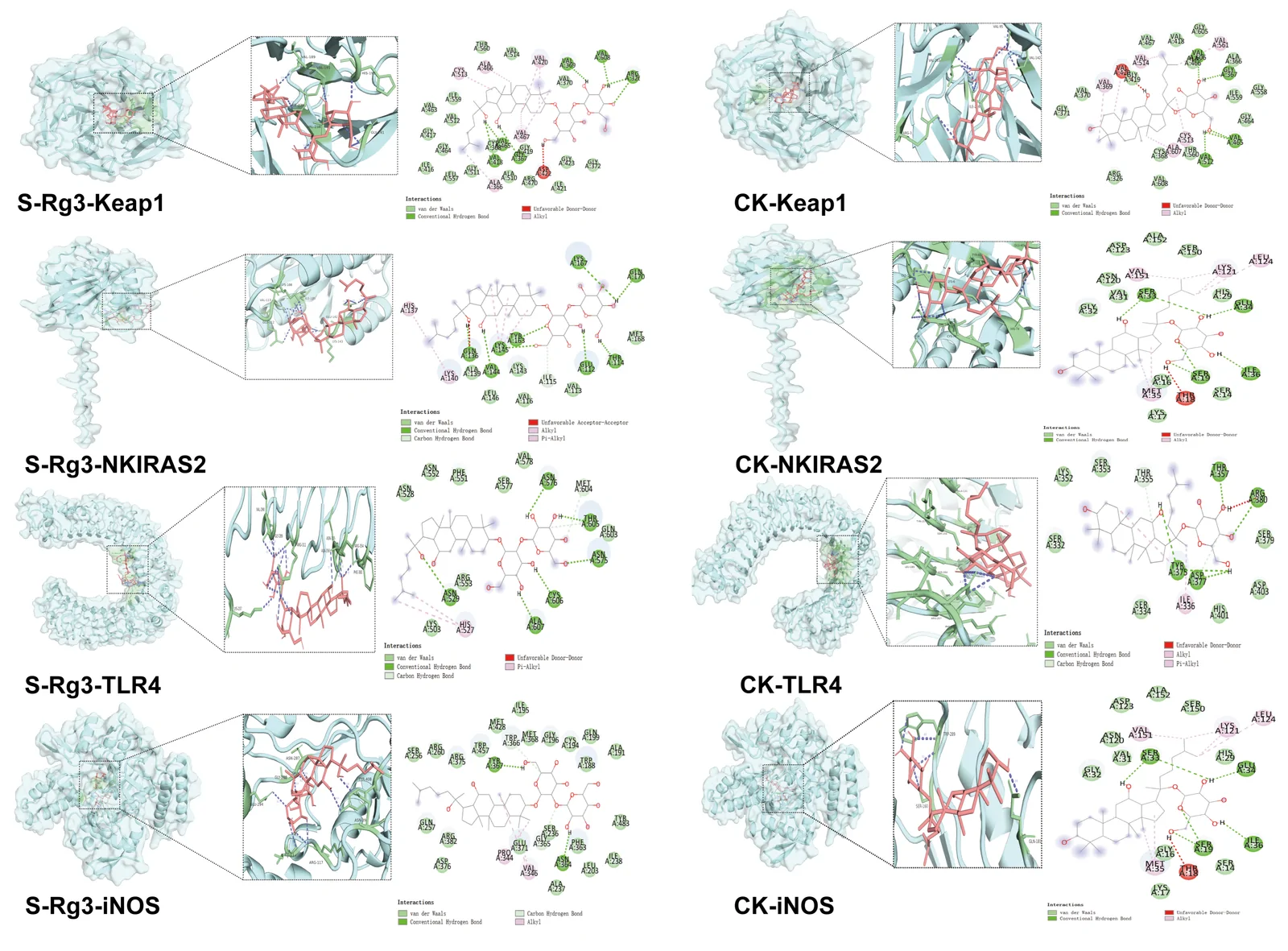
Representative molecular docking conformations of S-Rg3 and Compound K with Keap1, NKIRAS2, TLR4, and iNOS. Three-dimensional binding conformations, enlarged binding regions, and corresponding two-dimensional interaction diagrams are shown for each ligand–target complex. Major predicted non-covalent interactions with surrounding amino acid residues are indicated in the two-dimensional diagrams.

As shown in Figure 3, S-Rg3 and CK occupied predicted binding regions of Keap1, TLR4, NKIRAS2, and iNOS. The three-dimensional docking conformations indicated that both ligands could be accommodated within the corresponding receptor binding regions, while the two-dimensional interaction diagrams revealed multiple non-covalent contacts with surrounding amino acid residues. As summarized in Table 2, these interactions mainly included conventional hydrogen bonds together with hydrophobic interactions such as alkyl, π–alkyl, and π–sigma contacts, depending on the ligand–target complex. For example, S-Rg3 formed hydrogen-bond interactions with Tyr367 and Asn364 in iNOS, several residues including Cys368 and Val465 in Keap1, and multiple residues in NKIRAS2 and TLR4. CK likewise formed multiple hydrogen-bond and hydrophobic contacts with all four target proteins.

Collectively, these docking results suggest that S-Rg3 and CK have favorable predicted interaction potential with proteins associated with inflammatory and redox-related regulation. These computational findings, together with the LC–MS and relative binding-energy analyses, supported the selection of S-Rg3 and CK for subsequent molecular dynamics simulations and cellular validation.

### 3.3. Molecular Dynamics Simulation Confirms the Stability of S-Rg3 and CK Complexes with Key Targets

Following molecular docking analysis, 100 ns molecular dynamics (MD) simulations were performed to further evaluate the dynamic stability of S-Rg3 and CK complexes with Keap1, NKIRAS2, TLR4, and iNOS. Several parameters, including RMSD, radius of gyration (Rg), solvent-accessible surface area (SASA), RMSF, hydrogen bond number, MM-PBSA binding free energy, and residue energy decomposition, were used to assess the conformational stability and binding characteristics of each ligand–receptor complex.

**Figure 4:**
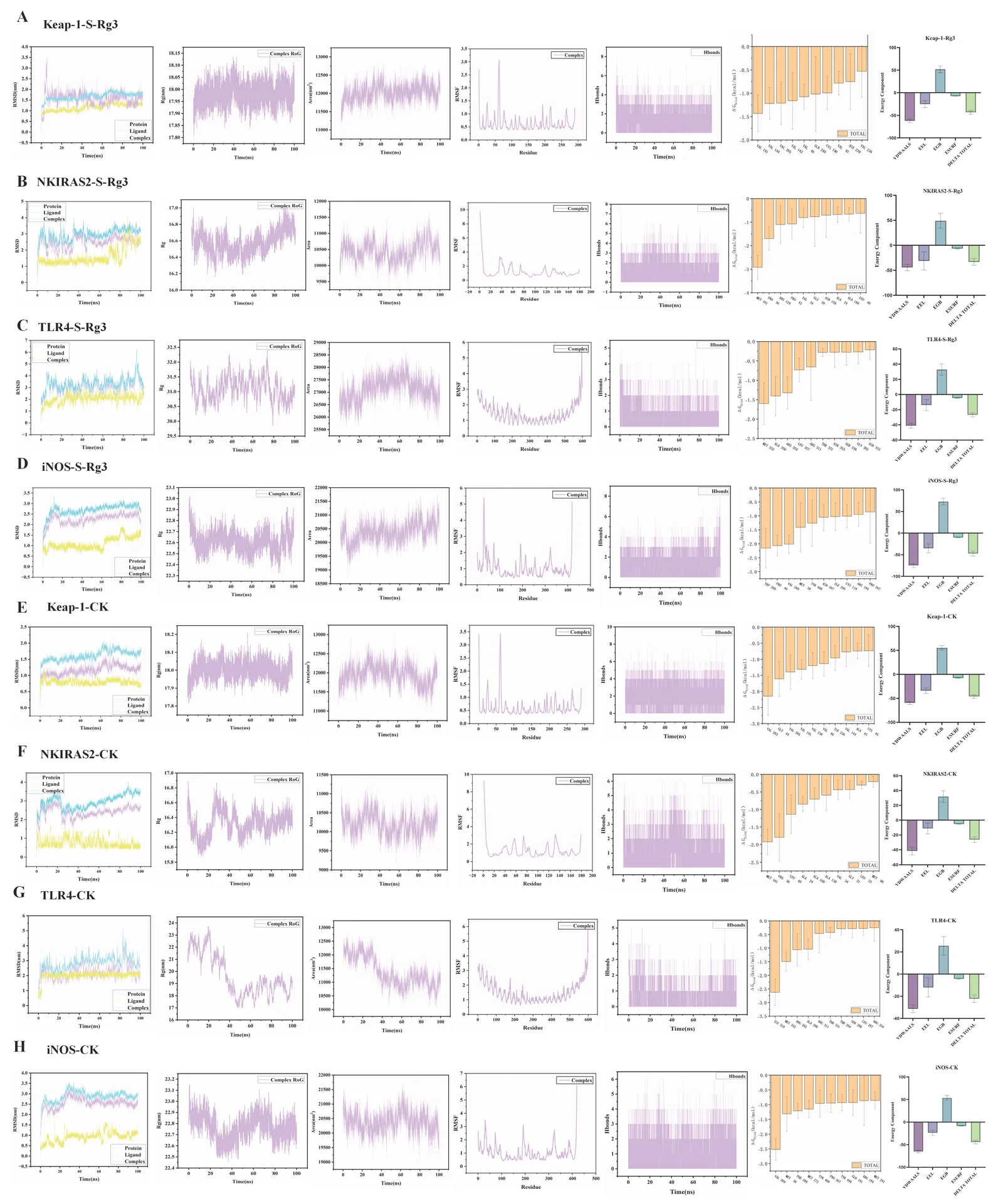
Molecular dynamics simulation of S-Rg3/CK–target protein complexes. (**A**–**D**) Stability and binding characteristics of S-Rg3 with Keap1, NKIRAS2, TLR4, and iNOS. (**E**–**H**) Stability and binding characteristics of CK with Keap1, NKIRAS2, TLR4, and iNOS. RMSD, Rg, SASA, RMSF, hydrogen bonds, residue energy decomposition, and binding free energy components were calculated to assess complex stability and key interaction patterns during the 100-ns simulation.

As shown in Figure 4A–H, the RMSD curves of the S-Rg3- and CK-bound complexes showed an initial adjustment phase and then fluctuated within a relatively stable range during the 100 ns simulations. No obvious continuous conformational drift was observed, suggesting that the complexes generally maintained stable conformations after equilibration. The Rg and SASA curves did not show persistent increasing or decreasing trends, indicating that the overall compactness and solvent exposure of the complexes remained relatively stable throughout the simulations. RMSF analysis further showed that most amino acid residues exhibited low fluctuation amplitudes, whereas relatively higher flexibility was mainly observed in several local regions.

Hydrogen bond analysis showed that S-Rg3 and CK maintained dynamic hydrogen bond interactions with Keap1, NKIRAS2, TLR4, and iNOS during the simulations. Although the number of hydrogen bonds varied over time, ligand–receptor interactions were continuously observed in each complex. MM-PBSA binding free energy analysis showed that all complexes had negative total binding free energies, suggesting favorable predicted binding stability between S-Rg3 or CK and the selected targets. Energy decomposition analysis further indicated that van der Waals and electrostatic interactions were the main favorable contributors to ligand–receptor binding, whereas polar solvation energy contributed unfavorably.

Overall, the 100 ns MD simulation results were consistent with the molecular docking findings and predicted that S-Rg3 and CK could form dynamically stable complexes with Keap1, NKIRAS2, TLR4, and iNOS. These results provide computational support for selecting S-Rg3 and CK as representative candidate compounds for subsequent cellular validation.

### 3.4. Network Pharmacology and Candidate Target Screening of Red Ginseng-Derived Compounds Against Calf Diarrhea-Related Intestinal Inflammatory Injury

To explore the potential targets and pathways through which red ginseng candidate compounds may regulate calf diarrhea-related intestinal inflammatory injury, network pharmacology analysis was performed. As shown in Figure S2A, 182 potential compound-related targets and 5382 calf diarrhea-related targets were identified, with 142 overlapping targets shared between the two datasets. A compound–target–disease network was then constructed based on the seven database-screened candidate compounds and the 142 overlapping targets (Figure S2B). Among these compounds, 20(S)-Ginsenoside Rg3 and Rg3-related entries were included in the subsequent candidate molecule analysis.

PPI network analysis of the 142 overlapping targets revealed several highly connected hub nodes, including IL6, TLR4, PTGS2, NFKB1, NOS2, MAPK8, MAPK14, CASP3, BCL2, MTOR, PPARG, ESR1, HSP90AA1, and HSP90AB1 (Figure S2C,D). These targets are closely associated with LPS recognition, inflammatory signaling, oxidative stress, apoptosis, and autophagy-related cellular responses, suggesting that red ginseng candidate compounds may act on multiple nodes involved in intestinal inflammatory injury.

**Figure 5:**
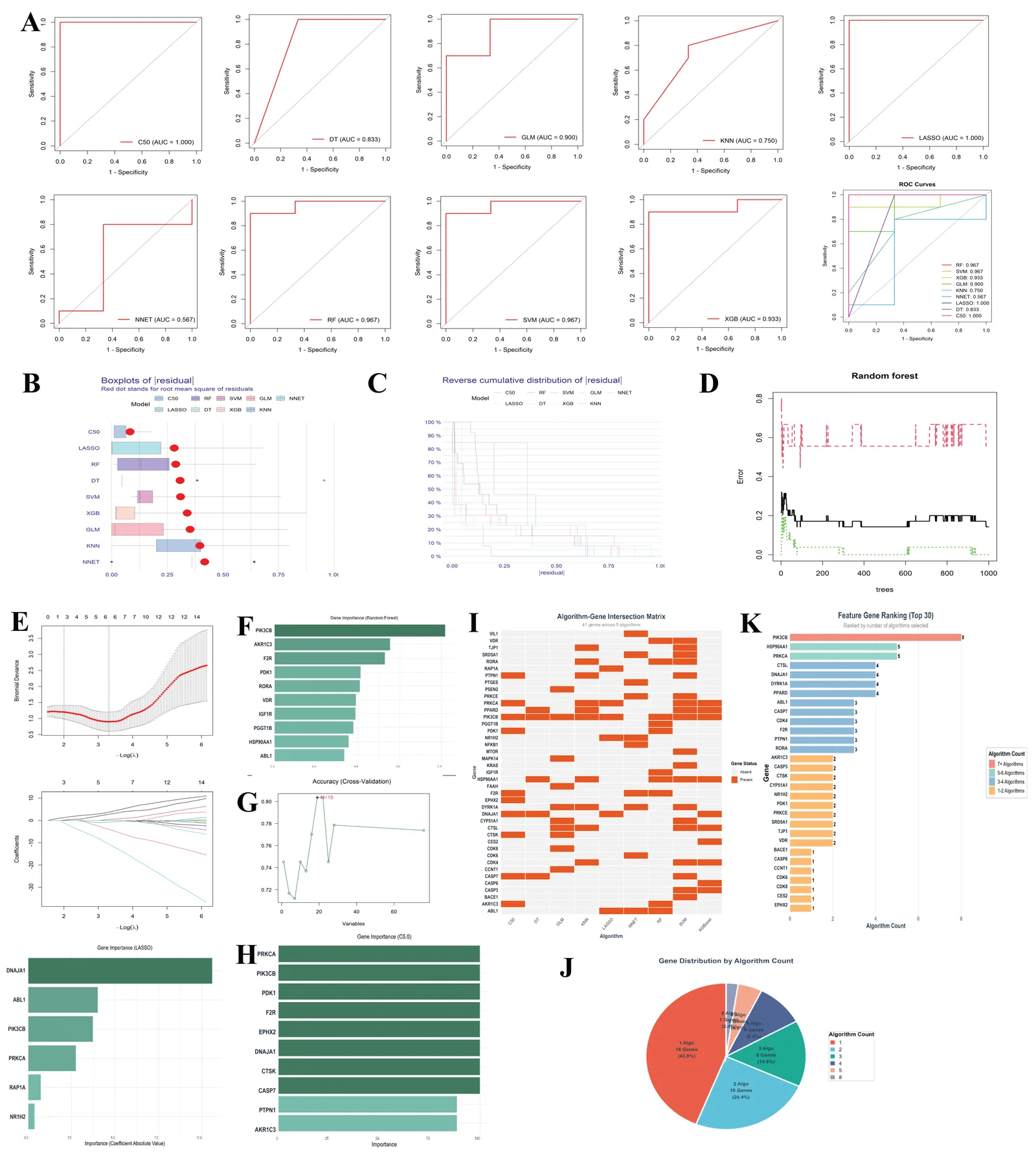
Machine-learning-based identification of key candidate genes. (**A**) ROC curves of different machine-learning models. (**B**,**C**) Residual distribution and reverse cumulative residual distribution of the models. (**D**) Random forest error curve. (**E**) LASSO regression analysis. (**F**,**H**) Gene importance ranking based on random forest and C5.0 models. (**G**) Cross-validation accuracy of different models. (**I**) Algorithm–gene intersection matrix showing genes selected by different algorithms. (**J**,**K**) Distribution and ranking of candidate genes according to the number of algorithms by which they were selected. *PIK3CB*, *HSP90AA1*, *PRKCA*, *CTSL*, and *CASP7* were identified as potential key genes for further validation.

To further prioritize the candidate hub targets and reduce the bias associated with network topology alone, machine-learning analysis was performed using network pharmacology-derived hub genes and an external public transcriptomic dataset related to intestinal inflammatory injury. As shown in Figure 5A, several machine-learning models showed high discriminatory performance, with C5.0 and LASSO achieving the highest AUC values, followed by RF, SVM, XGB, and GLM. Residual distribution, reverse cumulative residual analysis, random forest error curves, and cross-validation accuracy were further used to evaluate model performance and stability (Figure 5B–D,G).

Feature-selection and gene-importance analyses using LASSO regression, random forest, and C5.0 identified multiple candidate genes with relatively high predictive importance (Figure 5E,F,H). To further assess the consistency of gene selection across algorithms, an algorithm–gene intersection matrix and frequency-ranking analysis were performed (Figure 5I–K). These analyses showed that PIK3CB was selected by the largest number of algorithms, followed by HSP90AA1 and PRKCA, with CTSL, CASP7, and several other genes also recurrently identified across multiple models. Collectively, these findings suggest that genes related to PI3K-associated signaling, cellular stress responses, lysosomal function, and apoptosis may represent important regulatory nodes involved in red ginseng candidate compound-mediated protection against intestinal inflammatory injury.

**Figure 6:**
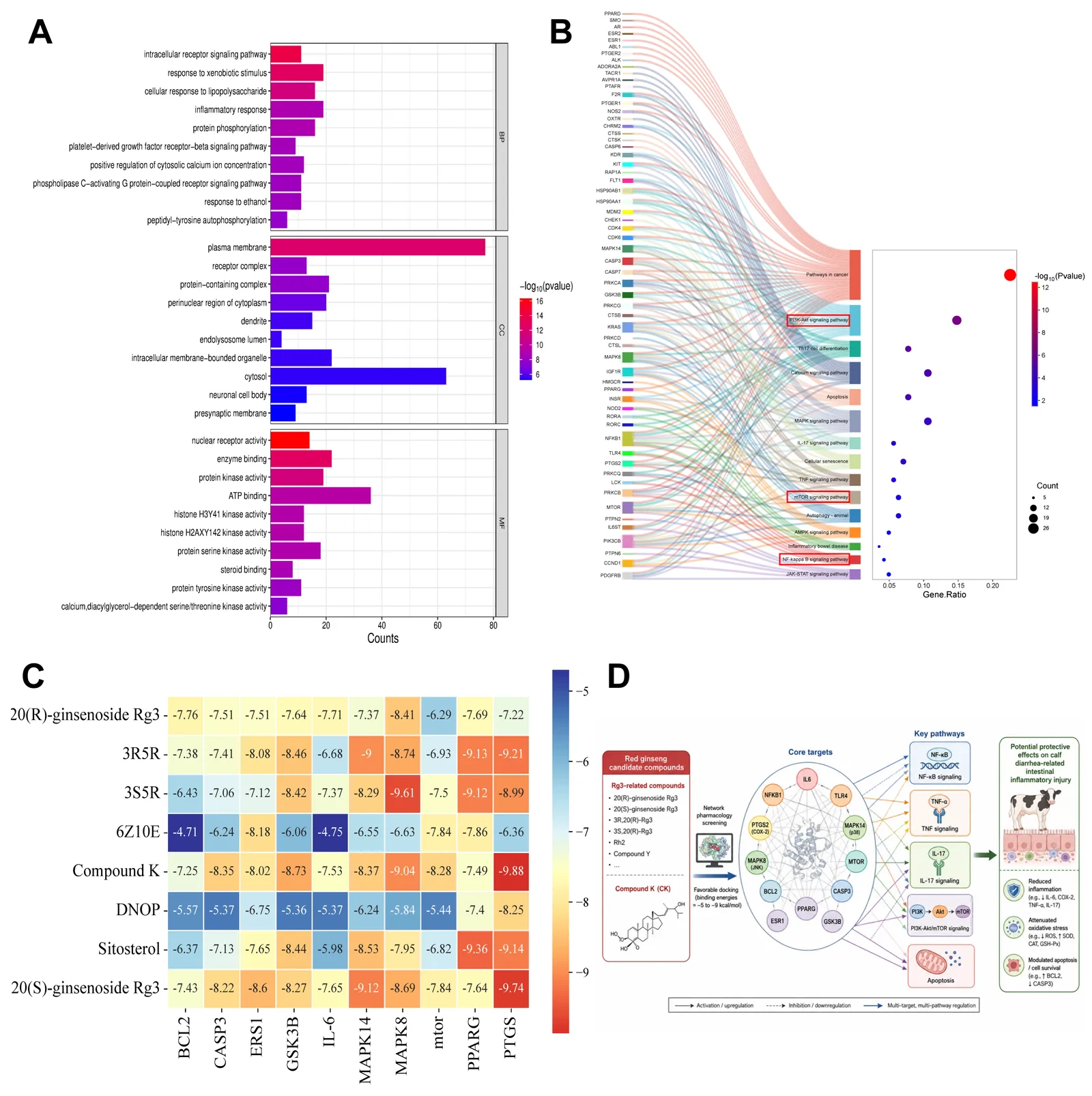
Network pharmacology and docking analysis of red ginseng candidate compounds. (**A**) GO enrichment analysis. (**B**) KEGG enrichment analysis and pathway–target network. (**C**) Docking binding energy heatmap. (**D**) Schematic overview of predicted multi-target and multi-pathway associations.

GO enrichment analysis further showed that the overlapping targets were mainly associated with response to lipopolysaccharide, inflammatory response, intracellular receptor signaling, protein phosphorylation, enzyme binding, and protein kinase activity (Figure 6A). KEGG enrichment analysis showed that these targets were enriched in inflammation- and epithelial injury-related pathways, including NF-κB signaling pathway, TNF signaling pathway, IL-17 signaling pathway, MAPK signaling pathway, JAK-STAT signaling pathway, PI3K-Akt signaling pathway, mTOR signaling pathway, apoptosis, autophagy, and inflammatory bowel disease (Figure 6B). Pathway–target network analysis further indicated that NFKB1, TLR4, IL6, PTGS2, NOS2, MAPK8, MAPK14, CASP3, BCL2, and MTOR participated in multiple enriched pathways, supporting the involvement of an inflammation–redox–apoptosis/autophagy regulatory network.

Although CK was not included among the seven candidate compounds obtained from the initial database screening, it was detected in red ginseng processing by-products by LC-MS and showed favorable performance in relative binding energy evaluation. Therefore, CK was included together with 20(S)-Ginsenoside Rg3 as a representative transformed ginsenoside for subsequent analysis. As shown in Figure 6C, both 20(S)-Ginsenoside Rg3 and CK exhibited predicted binding potential with representative hub targets related to inflammation, oxidative stress, apoptosis, and autophagy regulation. Moreover, molecular dynamics simulation further supported the stability of S-Rg3/CK–target protein complexes (Figure 4). By integrating LC-MS detection, network pharmacology, PPI hub target screening, machine-learning analysis, pathway enrichment, molecular docking, and molecular dynamics simulation, S-Rg3 and CK were selected as representative candidate compounds for further cellular and multi-omics validation.

### 3.5. S-Rg3 and CK Alleviate LPS-Induced Oxidative Stress and Inflammatory Responses

**Figure 7:**
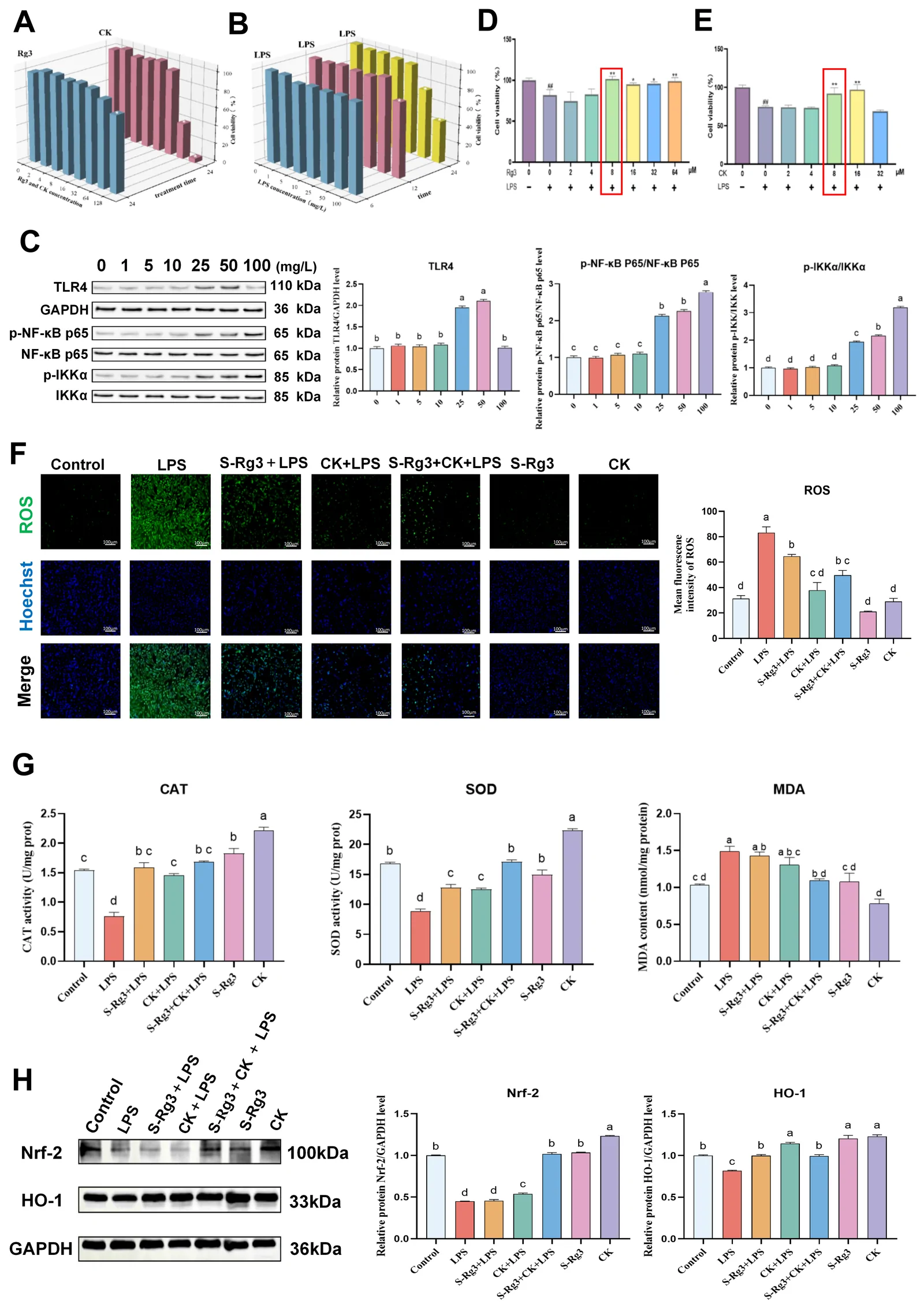
Determination of treatment conditions and effects of S-Rg3 and CK on LPS-induced oxidative stress in bovine small intestinal epithelial cells. (**A**) Cell viability following treatment with different concentrations of S-Rg3 and CK. (**B**) Cell viability following treatment with different concentrations of LPS at different exposure times. (**C**) Western blot analysis and quantification of TLR4, p-NF-κB p65/NF-κB p65, and p-IKK/IKK levels following LPS concentration-gradient treatment. (**D**,**E**) Effects of different concentrations of S-Rg3 or CK pretreatment on LPS-induced changes in cell viability. (**F**) Representative ROS fluorescence images and quantitative analysis. (**G**) CAT activity, SOD activity, and MDA content. (**H**) Western blot analysis and quantification of Nrf2 and HO-1 protein expression. The red box indicates the selected concentration (8 μM) used for subsequent experiments. The different colors of the bars are used only for visual distinction and do not represent additional scientific information. Data are presented as mean ± SEM. Different lowercase letters indicate significant differences among groups (*p* < 0.05); ## *p* < 0.01 vs. Control; * *p* < 0.05 and ** *p* < 0.01 vs. LPS.

To determine the treatment conditions for subsequent experiments, CCK-8 assays were first performed to evaluate the effects of S-Rg3, CK, and LPS on bovine intestinal epithelial cell viability. As shown in Figure 7A,B, low and moderate concentrations of S-Rg3 and CK showed no obvious cytotoxicity, whereas treatment with 50 mg/L LPS for 24 h significantly reduced cell viability (*p* < 0.05).

To further characterize the biological responsiveness of the LPS injury model, a concentration-gradient experiment was performed, and activation of the TLR4/IKK/NF-κB signaling pathway was evaluated by Western blotting. As shown in Figure 7C, TLR4 expression was markedly increased at higher LPS concentrations, with a pronounced elevation at 50 mg/L. Consistently, the p-IKK/IKK and p-NF-κB p65/NF-κB p65 ratios increased with increasing LPS concentrations, indicating robust activation of TLR4-associated inflammatory signaling under the selected LPS challenge condition.

Under LPS-induced injury conditions, 8 μM S-Rg3 and 8 μM CK significantly restored cell viability compared with the LPS group (*p* < 0.05; Figure 7D,E). To further evaluate the safety of the combined treatment, an additional fixed-ratio concentration–response assay was performed using S-Rg3 and CK at 1:1 combinations of 1 + 1, 2 + 2, 4 + 4, 8 + 8, and 16 + 16 μM. As shown in Figure S3, combined treatment at concentrations up to 8 + 8 μM did not significantly affect cell viability compared with the Control group, whereas 16 + 16 μM significantly reduced cell viability (*p* < 0.05). Fixed-ratio interaction analysis further indicated a positive interaction trend between S-Rg3 and CK, as supported by the corresponding Fa values and positive Bliss/HSA interaction scores (Figure S3). Based on the concentration-response, cytotoxicity, and interaction analyses, 8 μM S-Rg3, 8 μM CK, 8 μM S-Rg3 + 8 μM CK, and 50 mg/L LPS were selected as the working conditions for subsequent experiments.

The effects of S-Rg3 and CK on LPS-induced oxidative stress were then evaluated. As shown in Figure 7F–H, LPS treatment markedly increased ROS accumulation and MDA content, decreased CAT and SOD activities, and reduced Nrf2 and HO-1 protein expression (*p* < 0.05), indicating pronounced oxidative stress and impaired antioxidant defense. Compared with the LPS group, pretreatment with S-Rg3, CK, or their combination significantly alleviated these alterations to varying degrees, as evidenced by reduced ROS and MDA levels, restored CAT and SOD activities, and increased Nrf2 and HO-1 expression (*p* < 0.05). Overall, the combined pretreatment showed a more pronounced improvement in antioxidant-related indicators.

**Figure 8:**
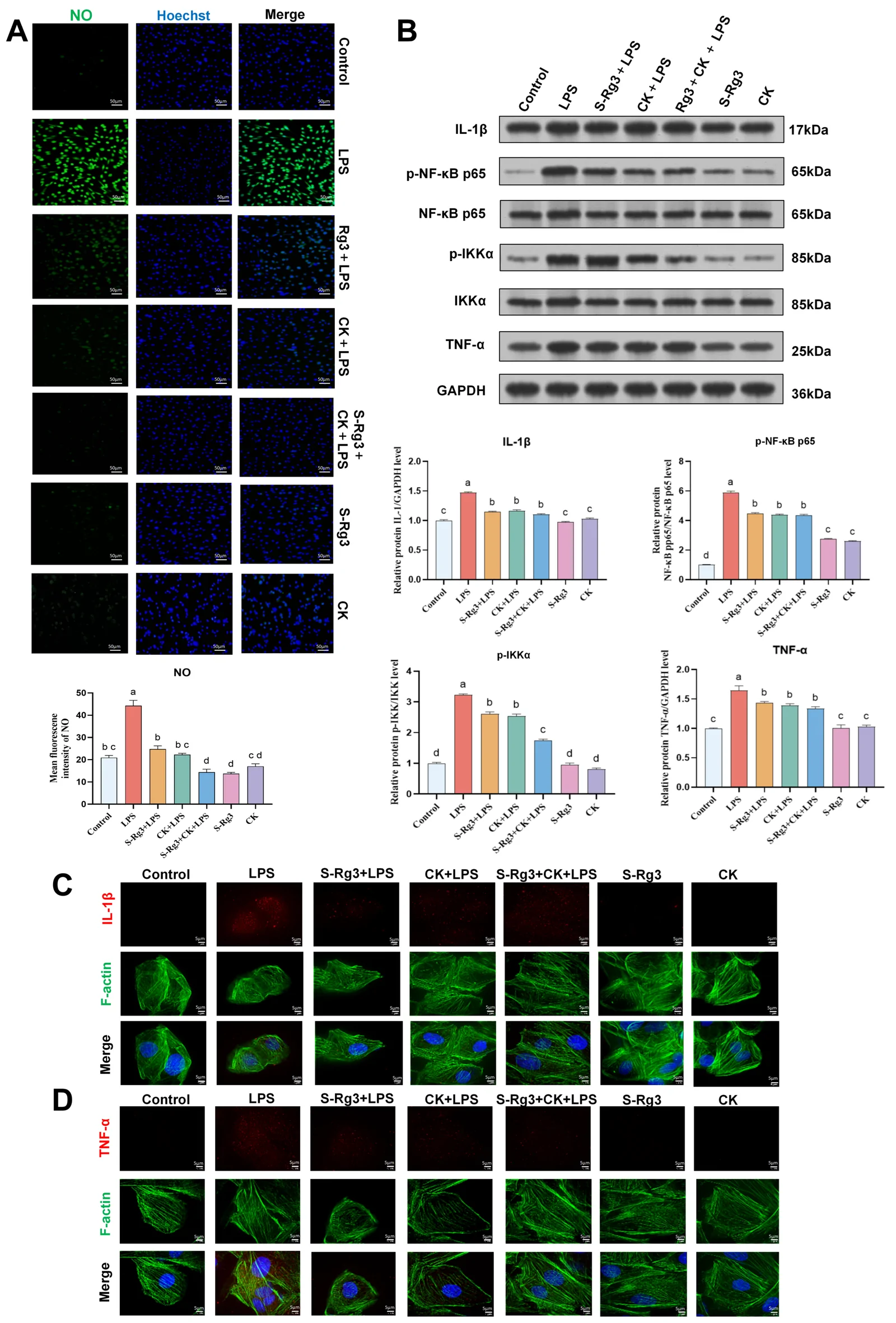
Effects of S-Rg3 and CK on LPS-induced inflammatory responses in bovine intestinal epithelial cells. (**A**) NO fluorescence images and quantification. (**B**) Western blot analysis and quantification of IL-1β, p-NF-κB p65/NF-κB p65, p-IKK/IKK, and TNF-α. (**C**,**D**) Immunofluorescence staining of IL-1β and TNF-α, respectively. Data are presented as mean ± SEM. Different lowercase letters indicate significant differences among groups (*p* < 0.05).

Inflammatory responses were further assessed by NO detection, Western blotting, and immunofluorescence staining. As shown in Figure 8A–D, LPS treatment significantly increased intracellular NO production and elevated IL-1β, p-NF-κB p65/NF-κB p65, p-IKK/IKK, and TNF-α levels compared with the Control group (*p* < 0.05). Consistently, immunofluorescence staining showed enhanced IL-1β and TNF-α signals following LPS exposure. Compared with the LPS group, pretreatment with S-Rg3, CK, or their combination significantly reduced NO production and suppressed inflammatory signaling and cytokine expression to varying degrees (*p* < 0.05). Although the combined pretreatment produced a pronounced reduction in NO production, it did not consistently show greater inhibition of IKK/NF-κB-associated inflammatory activation than either individual treatment. These findings indicate that S-Rg3 and CK effectively attenuated LPS-induced inflammatory responses in bovine intestinal epithelial cells.

### 3.6. S-Rg3 and CK Preserve Tight Junction Protein Expression in LPS-Treated Bovine Small Intestinal Epithelial Cells

**Figure 9:**
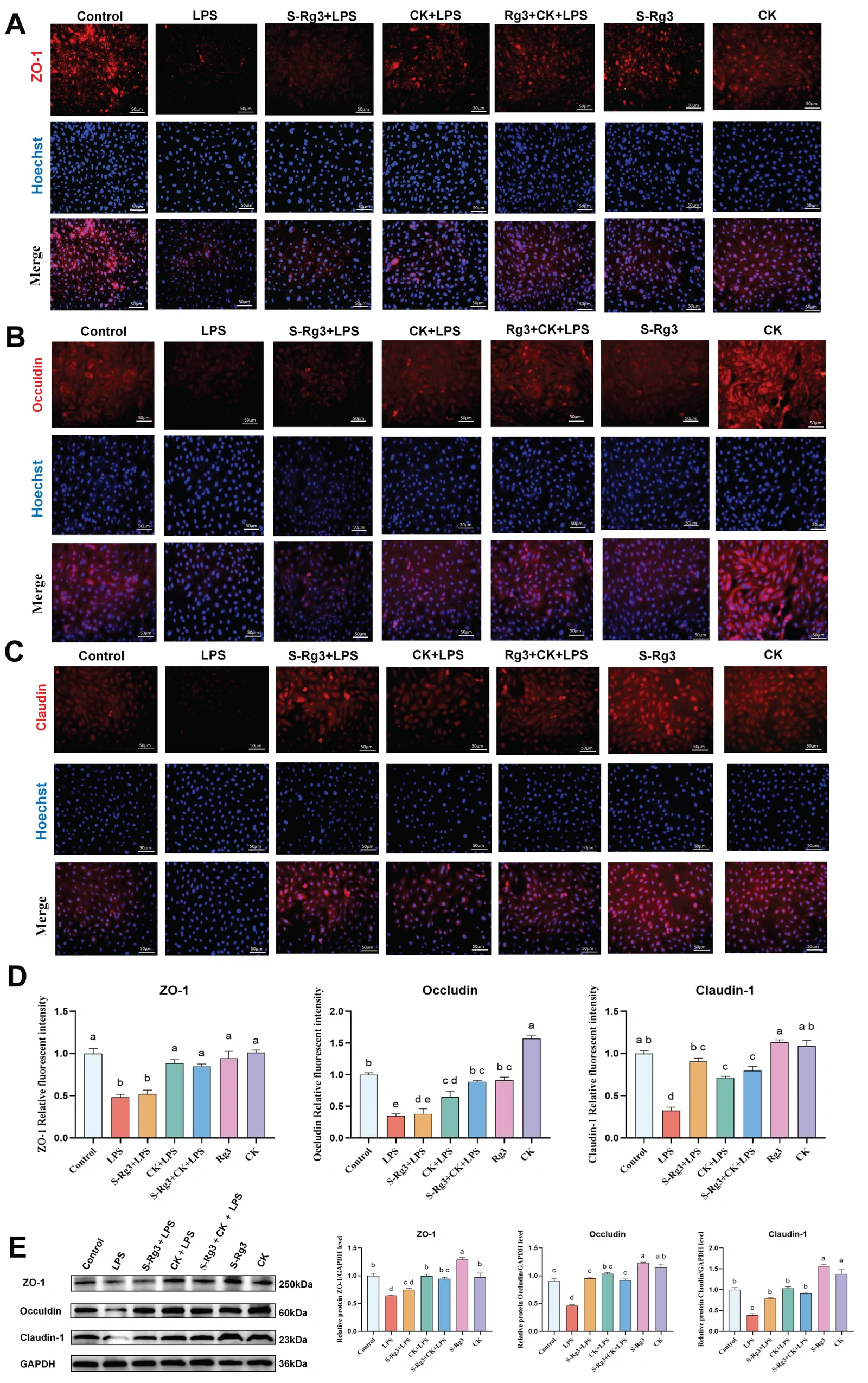
Effects of S-Rg3 and CK on tight junction proteins in LPS-treated bovine small intestinal epithelial cells. (**A**–**C**) Immunofluorescence images of ZO-1, Occludin, and Claudin-1. Red fluorescence indicates tight junction proteins, and blue fluorescence indicates nuclei. (**D**) Quantification of fluorescence intensity. (**E**) Western blot analysis and quantification of protein expression. Data are presented as mean ± SEM. Different lowercase letters indicate significant differences among groups (*p* < 0.05).

To evaluate whether S-Rg3 and CK could alleviate LPS-induced epithelial barrier injury, the expression and distribution of tight junction proteins were examined by immunofluorescence staining and Western blotting. As shown in Figure 9A–D, LPS treatment markedly weakened the fluorescence signals of ZO-1, Occludin, and Claudin-1 and significantly reduced their fluorescence intensity compared with the Control group (*p* < 0.05), indicating disruption of tight junction protein distribution. Compared with the LPS group, pretreatment with S-Rg3, CK, or their combination restored the fluorescence signals and intensities of these tight junction proteins to varying degrees (*p* < 0.05), with the extent of recovery varying across individual proteins and treatments. Consistently, Western blot analysis showed that LPS significantly decreased ZO-1, Occludin, and Claudin-1 protein expression, whereas S-Rg3, CK, and their combined pretreatment increased their expression levels (*p* < 0.05; Figure 9E). These results indicate that S-Rg3 and CK alleviated LPS-induced tight junction disruption and helped maintain epithelial barrier-associated protein expression in bovine intestinal epithelial cells.

### 3.7. Effects of S-Rg3, CK, and Their Combined Pretreatment on LPS-Induced Transcriptomic Changes

#### 3.7.1. Overall Distribution of Transcriptomic Samples

To evaluate the overall expression differences and within-group reproducibility of transcriptomic samples, PCA was performed based on the gene expression matrix. As shown in Figure S4A, samples from the Control and LPS groups were clearly separated in the principal component space, indicating that LPS treatment altered the overall transcriptomic profile of bovine small intestinal epithelial cells. When the LPS group was used as the reference, samples from the S-Rg3 + LPS, CK + LPS, and S-Rg3 + CK + LPS groups also showed varying degrees of separation from the LPS group, indicating that S-Rg3, CK, and their combined pretreatment affected the transcriptomic pattern after LPS exposure. Meanwhile, samples within each treatment group clustered well, suggesting good within-group consistency and suitability for subsequent differential expression and functional enrichment analyses.

#### 3.7.2. Identification of Differentially Expressed Genes

Differential expression analysis was performed using |log2(FoldChange)| > 1 and adjusted *p* value < 0.05 as the screening criteria. As shown in Figure S4B, LPS stimulation markedly altered the transcriptomic profile of bovine intestinal epithelial cells and prominently upregulated inflammation-related genes, including *CXCL8*, *CXCL2*, *GRO1*, *CCL20*, and *IL1B*, indicating a strong inflammatory transcriptional response. Compared with the LPS group, S-Rg3, CK, and especially their combined pretreatment remodeled the LPS-induced DEG profiles and were associated with reduced inflammatory activation and altered expression of epithelial adhesion- and stress-response-related genes. These results suggest that S-Rg3 and CK modulated LPS-induced transcriptional reprogramming, with the combined pretreatment showing a broader effect on inflammatory epithelial injury.

#### 3.7.3. GO Functional Enrichment Analysis of Differentially Expressed Genes

**Figure 10:**
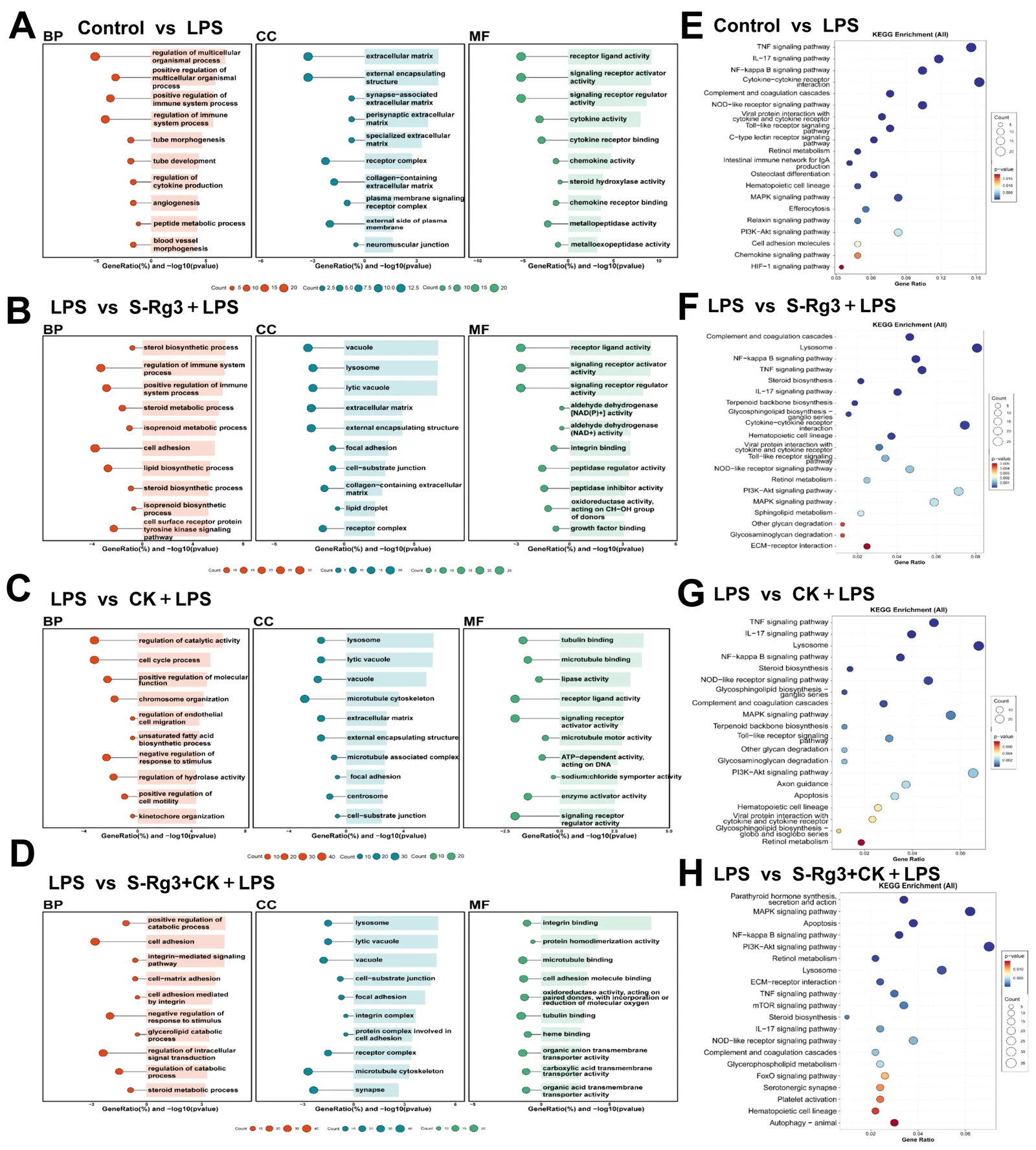
Transcriptomic analysis of LPS-treated bovine small intestinal epithelial cells under S-Rg3, CK, and combined pretreatment. (**A**–**D**) GO enrichment analysis. (**E**–**H**) KEGG pathway enrichment analysis.

To further characterize the biological functions associated with LPS-induced transcriptional changes, GO enrichment analysis was performed for each comparison. As shown in Figure 10A–D, DEGs in the Control vs. LPS comparison were mainly enriched in inflammation- and immune-related terms, including immune system regulation, cytokine production, response to lipopolysaccharide, cytokine activity, chemokine activity, and receptor ligand activity. These results further confirmed that LPS induced a strong inflammatory and immune response in bovine intestinal epithelial cells. Compared with the LPS group, S-Rg3, CK, and their combined pretreatment altered the GO enrichment patterns of LPS-responsive DEGs, with enriched functions mainly involving immune regulation, lipid metabolic processes, extracellular matrix organization, cell adhesion, cytoskeletal remodeling, lysosome/vacuole-related components, and integrin-mediated signaling. Notably, the combined pretreatment showed prominent enrichment in cell adhesion, cell–matrix adhesion, focal adhesion, and integrin-related functions, suggesting that S-Rg3 and CK may attenuate LPS-induced inflammatory epithelial injury while supporting epithelial structural stability and barrier-associated repair.

#### 3.7.4. KEGG Pathway Enrichment Analysis

To further identify the signaling pathways associated with LPS-induced inflammatory epithelial injury, KEGG enrichment analysis was performed using DEGs from each comparison. As shown in Figure 10E–H, DEGs in the Control vs. LPS comparison were mainly enriched in inflammation-, immunity-, and cell death-related pathways, including TNF signaling pathway, IL-17 signaling pathway, NF-kappa B signaling pathway, NOD-like receptor signaling pathway, cytokine-cytokine receptor interaction, MAPK signaling pathway, and apoptosis. These enriched pathways were consistent with the cellular results showing LPS-induced inflammatory activation, redox imbalance, and epithelial injury. Compared with the LPS group, DEGs in the S-Rg3 + LPS and CK + LPS groups were mainly associated with inflammatory signaling, cell adhesion, and cytoskeletal regulation, indicating that both compounds modulated LPS-responsive inflammatory and barrier-related transcriptional programs. Notably, the S-Rg3 + CK + LPS group showed broader enrichment involving PI3K-Akt signaling pathway, mTOR signaling pathway, ECM-receptor interaction, focal adhesion, autophagy, lysosome, apoptosis, and NF-kappa B signaling pathway, suggesting coordinated regulation of inflammation, epithelial barrier integrity, and autophagy–lysosome-associated cellular homeostasis.

KEGG enrichment analysis of upregulated and downregulated DEGs further supported this inflammation-centered regulatory pattern (Figure S5A–D). In the Control vs. LPS comparison, upregulated DEGs were mainly enriched in inflammatory and immune-related pathways, including TNF, IL-17, NF-kappa B, NOD-like receptor signaling, and cytokine-cytokine receptor interaction, whereas downregulated DEGs were associated with PI3K-Akt signaling, MAPK signaling, and metabolism-related pathways. After S-Rg3 or CK pretreatment, downregulated DEGs were mainly enriched in NF-kappa B signaling pathway, TNF signaling pathway, and cytokine-cytokine receptor interaction, suggesting suppression of LPS-induced inflammatory signaling. In the combined pretreatment group, upregulated DEGs were enriched in PI3K-Akt signaling pathway, mTOR signaling pathway, ECM-receptor interaction, focal adhesion, and autophagy, whereas downregulated DEGs were enriched in apoptosis, NF-kappa B signaling pathway, and lysosome. These results indicate that S-Rg3 and CK, particularly in combination, may attenuate LPS-induced inflammatory epithelial injury by suppressing pro-inflammatory signaling while supporting barrier-associated adhesion pathways and autophagy–lysosome-related homeostasis.

#### 3.7.5. GSEA Analysis

GSEA was performed to further evaluate pathway-level transcriptomic changes under different treatment conditions (Figure S4C). In the Control vs. LPS comparison, LPS treatment showed significant positive enrichment of apoptosis (NES = 1.665, FDR = 0.037) and NOD-like receptor signaling pathway (NES = 2.398, FDR < 0.001), indicating activation of cell death- and inflammation-related transcriptional programs. The PI3K-Akt signaling pathway was also positively enriched after LPS exposure (NES = 1.501, FDR = 0.043), suggesting that LPS broadly affected cell survival-related signaling. Compared with the LPS group, S-Rg3 + LPS showed significant negative enrichment of apoptosis (NES = −1.771, FDR = 0.003) and NOD-like receptor signaling pathway (NES = −1.844, FDR = 0.001). Similarly, CK + LPS showed negative enrichment of apoptosis (NES = −1.632, FDR = 0.019) and NOD-like receptor signaling pathway (NES = −1.573, FDR = 0.032). These opposite enrichment directions suggest that S-Rg3 and CK partially reversed LPS-induced inflammatory and apoptotic transcriptional programs. In the LPS vs. S-Rg3 + CK + LPS comparison, PI3K-Akt signaling was significantly positively enriched (NES = 1.909, FDR < 0.001), whereas apoptosis and NOD-like receptor signaling showed negative enrichment trends. Overall, these results indicate that S-Rg3 and CK regulated LPS-induced transcriptomic alterations at the pathway level, with single pretreatments mainly suppressing inflammatory/apoptotic programs and combined pretreatment more prominently enhancing PI3K-Akt-related cell survival and repair signaling.

#### 3.7.6. Machine Learning-Based Screening of Core Candidate Genes

To further screen candidate genes associated with LPS-induced inflammatory epithelial injury and compound intervention, LASSO regression analysis was performed using DEGs between the LPS group and the intervention groups. As shown in Figure S4D, four feature genes, *CTSB*, *TLR4*, *MAP1LC3B*, and *BECN1*, were selected by cross-validation-based dimensionality reduction. Among them, *TLR4* is closely related to LPS recognition and inflammatory activation, whereas *CTSB*, *MAP1LC3B*, and *BECN1* are involved in autophagy–lysosome regulation. Additional exploratory machine-learning analyses (Figure S6) showed that *CTSB*, *MAP1LC3B*, and *BECN1* were repeatedly identified as important candidate genes, supporting the involvement of autophagy–lysosome-associated inflammatory regulation. Considering the limited sample size and lack of external validation, these results were interpreted as exploratory candidate gene screening rather than definitive biomarker identification.

### 3.8. Validation of Transcriptomic Findings Related to Stress Responses, Autophagy-Lysosome Function, and Apoptosis

#### 3.8.1. Effects of S-Rg3 and CK on HSP70 and HSP90 Protein Expression

**Figure 11:**
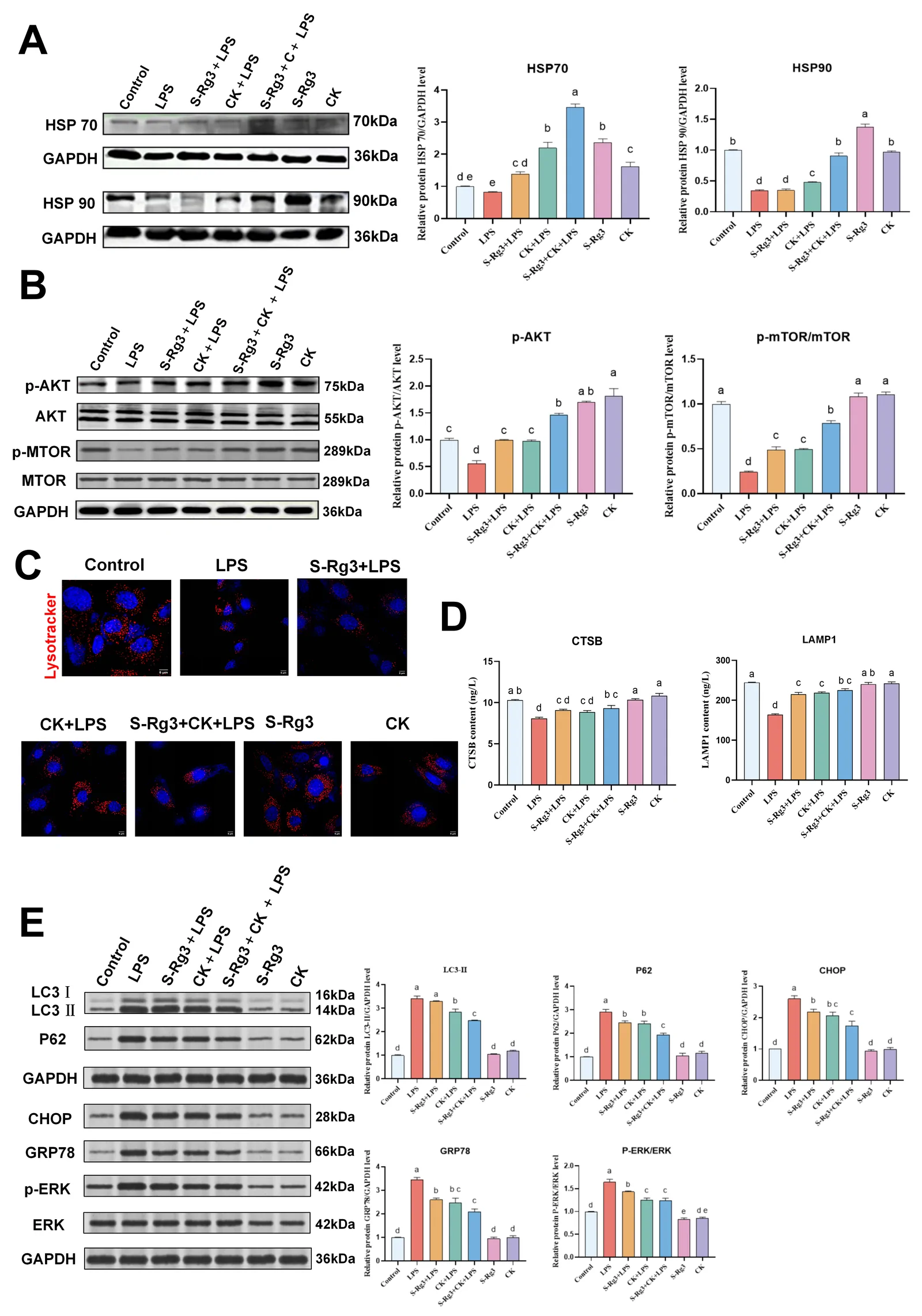
Effects of S-Rg3 and CK on cellular stress, AKT/mTOR signaling, lysosomal function, and autophagy-related responses in LPS-treated bovine intestinal epithelial cells. (**A**) HSP70 and HSP90 protein expression. (**B**) p-AKT/AKT and p-mTOR/mTOR levels. (**C**) Representative LysoTracker staining images. (**D**) CTSB and LAMP1 levels. (**E**) LC3-II, p62, CHOP, GRP78, and p-ERK/ERK protein expression. Data are presented as mean ± SEM. Different lowercase letters indicate significant differences among groups (*p* < 0.05).

To validate the transcriptomic changes related to cellular stress responses, the protein expression levels of HSP70 and HSP90 were examined. As shown in Figure 11A, HSP70 expression showed a decreasing trend in the LPS group compared with the Control group, although the difference was not significant (*p* > 0.05). Compared with the LPS group, HSP70 protein expression was significantly increased in the S-Rg3 + LPS, CK + LPS, S-Rg3 + CK + LPS, S-Rg3, and CK groups (*p* < 0.05), with the combined pretreatment showing the highest HSP70 expression among the LPS-challenged intervention groups. For HSP90, LPS treatment significantly reduced its protein expression compared with the Control group (*p* < 0.05). Compared with the LPS group, HSP90 expression was significantly increased in the CK + LPS, S-Rg3 + CK + LPS, S-Rg3, and CK groups (*p* < 0.05), whereas no significant difference was observed between the S-Rg3 + LPS and LPS groups (*p* > 0.05). Among all groups, the S-Rg3 alone group showed the highest HSP90 expression. These results indicate that S-Rg3, CK, and their combined pretreatment regulated HSP70 and HSP90 protein expression to varying degrees, with the combined pretreatment showing clear recovery of stress-related protein expression under LPS stimulation.

#### 3.8.2. Effects of S-Rg3 and CK on AKT/mTOR Signaling-Related Protein Expression

To further validate the transcriptomic changes related to the PI3K-Akt, mTOR, and autophagy-associated pathways, the protein expression levels of p-AKT/AKT and p-mTOR/mTOR were examined. As shown in Figure 11B, LPS treatment significantly reduced the levels of p-AKT/AKT and p-mTOR/mTOR compared with the Control group (*p* < 0.05). Compared with the LPS group, p-AKT/AKT levels were increased in the S-Rg3 + LPS, CK + LPS, and S-Rg3 + CK + LPS groups, whereas p-mTOR/mTOR showed the most evident recovery in the S-Rg3 + CK + LPS group. In the S-Rg3 and CK alone groups, p-AKT/AKT and p-mTOR/mTOR levels were maintained at relatively high levels. These results indicate that S-Rg3, CK, and their combined pretreatment restored the LPS-induced reduction in AKT/mTOR signaling to varying degrees, with the combined pretreatment showing a more evident restorative effect under LPS stimulation.

#### 3.8.3. Effects of S-Rg3 and CK on Lysosome-Related Indicators

To further validate the lysosome-related changes indicated by transcriptomic and machine-learning analyses, LysoTracker staining, CTSB content, and LAMP1 levels were examined. As shown in Figure 11C,D, LPS treatment reduced LysoTracker fluorescence intensity and decreased CTSB and LAMP1 levels compared with the Control group, indicating impaired lysosomal status in bovine intestinal epithelial cells (*p* < 0.05). Compared with the LPS group, S-Rg3, CK, and especially their combined pretreatment restored lysosome-related indicators to varying degrees, as shown by enhanced LysoTracker fluorescence and increased CTSB and LAMP1 levels (*p* < 0.05). These results suggest that S-Rg3 and CK alleviated LPS-induced lysosomal impairment, which may contribute to the recovery of autophagy–lysosome function.

#### 3.8.4. Effects of S-Rg3 and CK on Autophagy- and Endoplasmic Reticulum Stress-Related Protein Expression

To validate the transcriptomic changes related to autophagy–lysosome dysfunction and stress-associated pathways, LC3, P62, CHOP, GRP78, and p-ERK/ERK protein levels were examined. As shown in Figure 11E, LPS treatment significantly increased LC3-II and P62 levels, suggesting autophagy-related protein accumulation and possible impaired autophagic degradation rather than simple autophagy activation (*p* < 0.05). LPS also upregulated CHOP, GRP78, and p-ERK/ERK levels, indicating activation of endoplasmic reticulum stress and ERK signaling (*p* < 0.05). Compared with the LPS group, LC3-II levels were significantly reduced in the CK+LPS and S-Rg3+CK+LPS groups, but not in the S-Rg3+LPS group. In contrast, p62, CHOP, GRP78, and p-ERK/ERK levels were reduced to varying degrees by S-Rg3, CK, and their combined pretreatment (*p* < 0.05).These results indicate that S-Rg3 and CK alleviated LPS-induced autophagy–lysosome dysfunction and stress-associated signaling activation.

#### 3.8.5. Pharmacological Inhibition of Autophagy–Lysosomal Function Attenuates the Protective Effects of S-Rg3 and CK

**Figure 12:**
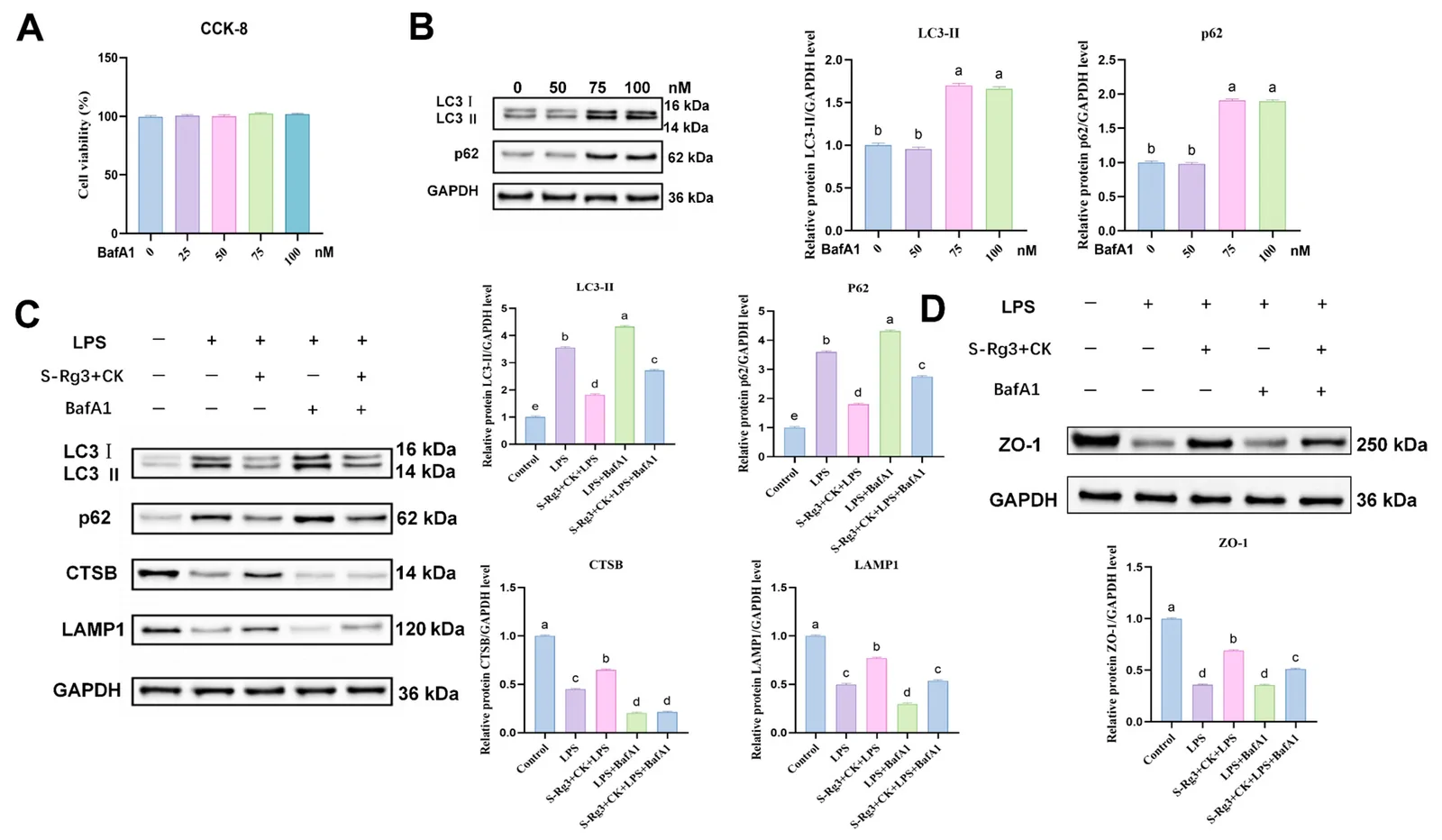
Pharmacological inhibition of autophagy–lysosomal function attenuates the protective effects of combined S-Rg3 and CK treatment. (**A**) Cell viability after treatment with different concentrations of Bafilomycin A1 (BafA1) for 6 h. (**B**) Western blot analysis and quantification of LC3-II and p62 following BafA1 concentration screening. (**C**) Western blot analysis and quantification of LC3-II, p62, CTSB, and LAMP1 in the five treatment groups. (**D**) Western blot analysis and quantification of ZO-1 expression. BafA1 was used at 75 nM for 6 h in the formal inhibition experiment. Data are presented as mean ± SEM. Different lowercase letters indicate significant differences among groups (*p* < 0.05).

Preliminary experiments showed that BafA1 treatment at 0–100 nM for 6 h did not significantly affect cell viability (Figure 12A). Western blot analysis further showed that 75 and 100 nM BafA1 markedly increased LC3-II and p62 accumulation, whereas lower concentrations produced relatively limited changes (Figure 12B). Therefore, 75 nM BafA1 was selected as the lowest effective concentration for subsequent inhibition experiments.

As shown in Figure 12C, LPS treatment increased LC3-II and p62 accumulation and decreased CTSB and LAMP1 levels, whereas combined S-Rg3 and CK pretreatment partially reversed these alterations. Importantly, addition of BafA1 markedly attenuated the effects of S-Rg3 and CK on LC3-II, p62, CTSB, and LAMP1. Consistently, S-Rg3 and CK restored the LPS-induced reduction in ZO-1 expression, whereas this protective effect was partially diminished following BafA1 treatment (Figure 12D). These findings indicate that pharmacological disruption of autophagy–lysosomal function weakens the protective effects of combined S-Rg3 and CK treatment, supporting a functional contribution of autophagy–lysosomal restoration to the maintenance of epithelial barrier integrity.

#### 3.8.6. Effects of S-Rg3 and CK on LPS-Induced Apoptosis and Mitochondrial Injury

**Figure 13:**
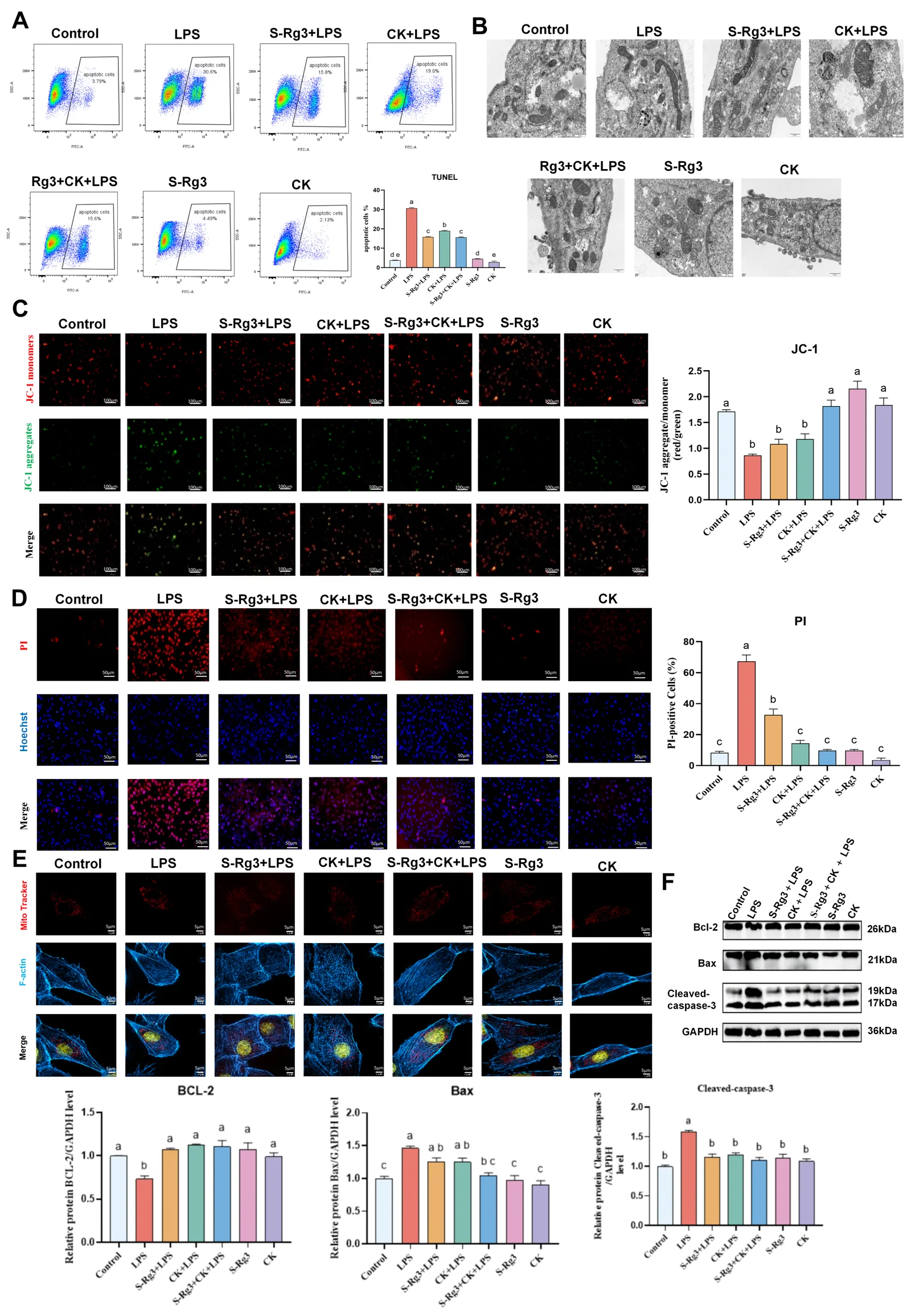
S-Rg3 and CK alleviate LPS-induced mitochondrial injury and apoptosis in bovine small intestinal epithelial cells. (**A**) TUNEL flow cytometry analysis and quantification of apoptotic cells. (**B**) Representative transmission electron microscopy images showing cellular and mitochondrial ultrastructural changes in the different treatment groups (Scale: 500nm). (**C**) JC-1 fluorescence staining and quantitative analysis of mitochondrial membrane potential; the JC-1 aggregate/monomer ratio was used to reflect changes in mitochondrial membrane potential. (**D**) Propidium iodide (PI) staining and quantification of PI-positive cells to assess cell membrane integrity and cell death. (**E**) Representative MitoTracker and F-actin fluorescence images showing mitochondrial and cytoskeletal morphology. (**F**) Western blot analysis and quantitative assessment of apoptosis-related proteins, including Bcl-2, Bax, and cleaved caspase-3. GAPDH was used as the internal loading control. Data are presented as mean ± SEM. Different lowercase letters indicate significant differences among groups (*p* < 0.05).

To further validate apoptosis- and mitochondrial injury-related transcriptomic changes, TUNEL flow cytometry, TEM, JC-1, PI, MitoTracker/F-actin staining, and apoptosis-related protein analysis were performed. As shown in Figure 13A–F, LPS treatment significantly increased apoptotic cell proportion and PI-positive cells, decreased mitochondrial membrane potential, disrupted mitochondrial ultrastructure and cytoskeletal organization, and promoted apoptosis-related protein changes, including decreased Bcl-2 and increased Bax and cleaved-caspase-3 levels (*p* < 0.05). Compared with the LPS group, S-Rg3, CK, and especially their combined pretreatment reduced apoptosis, improved mitochondrial membrane potential and cellular ultrastructure, decreased PI-positive cells, restored mitochondrial and cytoskeletal morphology, and partially normalized Bcl-2, Bax, and cleaved-caspase-3 expression (*p* < 0.05). These results indicate that S-Rg3 and CK alleviated LPS-induced mitochondrial injury, membrane damage, and apoptosis in bovine intestinal epithelial cells.

### 3.9. Untargeted Metabolomic Analysis of LPS-Induced Metabolic Changes and Ginsenoside-Mediated Regulation

#### 3.9.1. Untargeted Metabolomic Profiling and Differential Metabolite Screening

As shown in Figure S8A–D, PCA in both negative (NEG) and positive (POS) ionization modes showed clear separation between the Control and LPS groups, while the S-Rg3+LPS, CK+LPS, and S-Rg3+CK+LPS groups were also separated from the LPS group to varying degrees. PLS-DA and OPLS-DA analyses showed similar group-separation trends (Figure S7), indicating that LPS stimulation and S-Rg3/CK pretreatment markedly altered the extracellular metabolic profiles. Differential metabolite screening further identified distinct upregulated and downregulated metabolites across Control vs. LPS and LPS vs. intervention comparisons in both ionization modes (Figure S8E–H).

#### 3.9.2. KEGG Enrichment Analysis of Differential Metabolites

**Figure 14:**
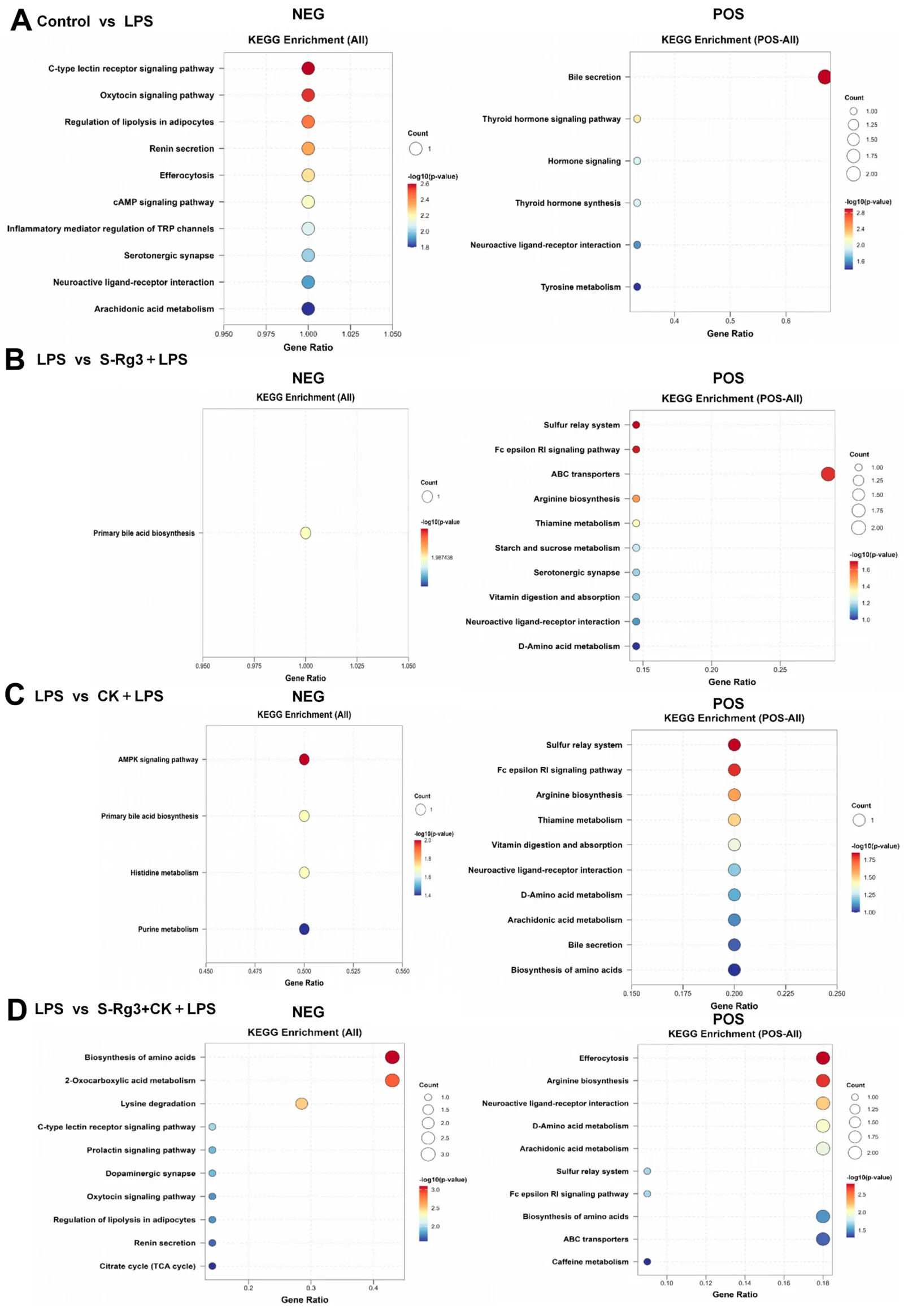
KEGG pathway enrichment analysis of differential metabolites in bovine small intestinal epithelial cells following LPS challenge and S-Rg3/CK pretreatment. (**A**) Control vs. LPS; (**B**) LPS vs. S-Rg3 + LPS; (**C**) LPS vs. CK + LPS; and (**D**) LPS vs. S-Rg3 + CK + LPS. For each comparison, KEGG enrichment results are shown separately for negative ionization mode (NEG) and positive ionization mode (POS). Bubble size indicates the number of differential metabolites enriched in the corresponding pathway, and bubble color represents −log10(*p* value). NEG, negative ionization mode; POS, positive ionization mode; KEGG, Kyoto Encyclopedia of Genes and Genomes.

To clarify the metabolic pathways associated with LPS-induced inflammatory epithelial injury and compound intervention, KEGG enrichment analysis was performed for differential metabolites in each comparison. As shown in Figure 14A–D, LPS-responsive metabolites were mainly enriched in arachidonic acid metabolism, inflammatory mediator regulation of TRP channels, C-type lectin receptor signaling pathway, bile secretion, and amino acid-related metabolic pathways, indicating that LPS induced extracellular metabolic remodeling associated with inflammatory lipid mediators, immune-related signaling, and amino acid metabolism. Compared with the LPS group, S-Rg3 and CK pretreatment mainly affected pathways related to arachidonic acid metabolism, arginine biosynthesis, sulfur relay system, primary bile acid biosynthesis, amino acid metabolism, and energy metabolism. These changes suggest that S-Rg3 and CK alleviated LPS-induced inflammatory metabolic disturbance by modulating inflammatory lipid mediator metabolism, arginine/NO-related metabolism, and redox-associated metabolic processes. The combined pretreatment group showed broader pathway enrichment involving biosynthesis of amino acids, 2-oxocarboxylic acid metabolism, citrate cycle, pyruvate metabolism, arachidonic acid metabolism, arginine biosynthesis, sulfur relay system, and efferocytosis, suggesting more extensive regulation of inflammation-related metabolism, energy remodeling, redox balance, and apoptotic cell clearance. KEGG enrichment of upregulated and downregulated metabolites further supported this pattern (Figure S9), indicating that S-Rg3, CK, and especially their combined pretreatment reshaped LPS-induced extracellular metabolic changes toward reduced inflammatory lipid mediator disturbance and improved metabolic homeostasis.

### 3.10. Integrated Transcriptomic and Untargeted Metabolomic Analysis

To characterize the coordinated changes between the transcriptome and untargeted metabolome, O2PLS-based integrative analysis was performed for each comparison. As shown in Figure S10A–D, LPS-induced high-contribution variables mainly included inflammation-related genes and lipid mediator-related metabolites. In the Control vs. LPS comparison, inflammatory cytokines and chemokines, including IL6, CXCL8, CXCL5, CXCL2, CCL20, and PTGES, together with metabolites such as Prostaglandin E2, contributed strongly to the joint transcriptomic–metabolomic variation, indicating that LPS-induced epithelial injury was closely associated with inflammatory transcriptional activation and inflammatory lipid mediator remodeling.

Compared with the LPS group, S-Rg3, CK, and their combined pretreatment altered the high-contribution gene–metabolite profiles. The major contributing variables in the intervention comparisons were mainly associated with inflammatory responses, lipid mediator metabolism, cellular stress, arginine/NO metabolism, amino acid metabolism, and redox-related metabolic processes. Notably, the combined pretreatment showed a broader regulatory profile, involving inflammatory genes such as *IL6*, stress-related genes such as *DDIT4* and *CHAC1*, and metabolites related to prostaglandin metabolism, L-arginine metabolism, and energy/redox-associated pathways. These results suggest that S-Rg3 and CK, especially in combination, reshaped the coordinated inflammatory and metabolic responses induced by LPS.

**Figure 15:**
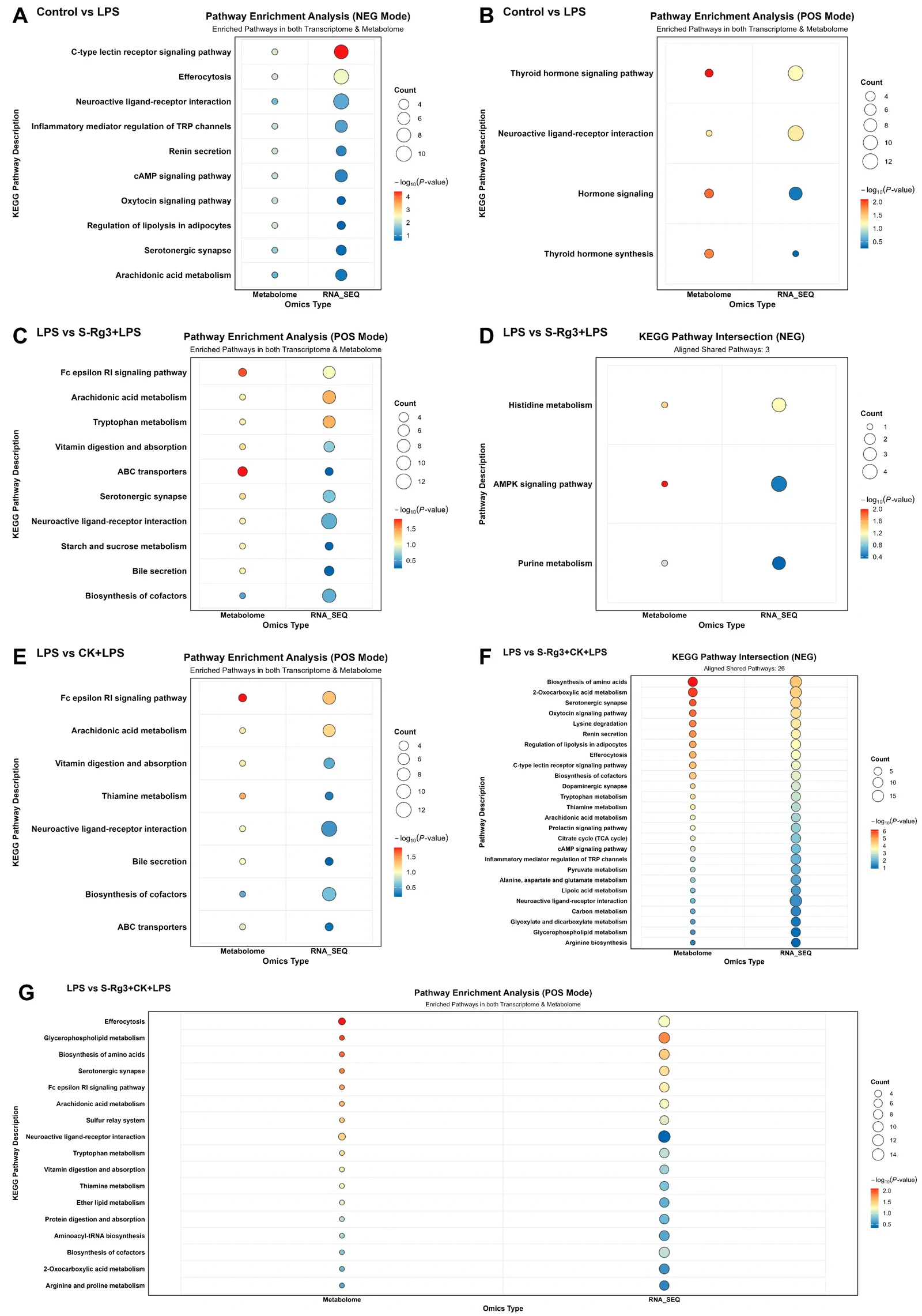
Integrated KEGG pathway enrichment analysis of transcriptomic and metabolomic data. (**A**,**B**) Control vs. LPS under NEG and POS modes; (**C**,**D**) LPS vs. S-Rg3 + LPS under POS and NEG modes; (**E**) LPS vs. CK + LPS under POS mode; (**F**,**G**) LPS vs. S-Rg3 + CK + LPS under NEG and POS modes. Bubble size indicates the number of differential features enriched in each pathway, and bubble color represents −log10(*p* value). NEG, negative ionization mode; POS, positive ionization mode.

Shared KEGG enrichment analysis further supported this inflammation-centered regulatory pattern (Figure 15A–G). LPS-responsive transcriptomic and metabolomic changes were commonly associated with inflammatory signaling and arachidonic acid metabolism, whereas S-Rg3 and CK pretreatment affected pathways related to arachidonic acid metabolism, arginine and proline metabolism, amino acid metabolism, sulfur relay system, and energy metabolism. The combined pretreatment showed broader shared enrichment involving amino acid biosynthesis, 2-oxocarboxylic acid metabolism, citrate cycle, pyruvate metabolism, arachidonic acid metabolism, sulfur relay system, glycerophospholipid metabolism, efferocytosis, and arginine and proline metabolism, indicating coordinated regulation of inflammation-related metabolism, redox balance, energy remodeling, and apoptotic cell clearance.

Pathway–gene–metabolite interaction networks further showed that LPS-induced networks were mainly centered on inflammatory cytokines, chemokines, and inflammation-related metabolites, including IL6, IL1B, PTGS2, NFKBIA, NFKB2, PTGER2, and PTGES (Figure S10E–K). After S-Rg3, CK, or combined pretreatment, the networks shifted toward reduced inflammatory signaling and broader metabolic regulation. Overall, the integrated transcriptomic and metabolomic analysis indicates that S-Rg3 and CK alleviated LPS-induced epithelial injury by coordinating inflammatory gene expression, inflammatory lipid mediator metabolism, arginine/NO metabolism, redox-related metabolism, and energy homeostasis.

## 4. Discussion

Calf diarrhea is a major intestinal disorder in young ruminant production and is closely associated with epithelial barrier dysfunction, endotoxin exposure, oxidative stress, inflammatory activation, and impaired growth performance. Although in vivo validation remains necessary, LPS-challenged bovine intestinal epithelial cells provide a useful in vitro system for investigating endotoxin-associated inflammatory epithelial injury. It should be emphasized that the 50 mg/L LPS concentration used in the present study represents a strong and supraphysiological in vitro challenge and should not be interpreted as directly reproducing the absolute LPS exposure occurring in calves with diarrhea. Nevertheless, the concentration-dependent activation of TLR4/IKK/NF-κB signaling observed in the present study supports the use of this condition as a robust model of LPS-associated inflammatory epithelial stress. In this study, S-Rg3 and CK derived from red ginseng processing by-products attenuated LPS-induced epithelial cytotoxicity, redox imbalance, inflammatory activation, tight junction disruption, autophagy–lysosome dysfunction, mitochondrial injury, and apoptosis. These findings suggest that red ginseng processing by-products may contain bioactive constituents with potential value for improving intestinal epithelial resilience in livestock production.

Red ginseng and its ginsenoside constituents have been widely reported to exert antioxidant, anti-inflammatory, and cytoprotective effects by regulating ROS production, antioxidant enzyme activity, and signaling pathways such as Nrf2/HO-1 and NF-κB [19]. During steaming, drying, and subsequent processing, several prototype ginsenosides can be converted into low-polarity rare ginsenosides, including Rg3, Rh2, Rg5, Rk1, and CK, through deglycosylation, dehydration, and isomerization [12]. These transformed ginsenosides generally exhibit better membrane permeability and stronger bioactivity than some prototype ginsenosides [20]. Consistent with these reports, the present LC–MS analysis confirmed that red ginseng processing by-products retained multiple ginsenoside-related constituents, including low-polarity or processing-related transformed ginsenosides such as Rg3, Rh2, Rk1, Rg5, and CK. Therefore, red ginseng processing by-products may serve as a valuable source of bioactive ginsenoside-related constituents, and representative rare ginsenosides retained in these by-products may contribute to the modulation of endotoxin-induced redox-associated epithelial injury.

In the present study, S-Rg3 and CK were selected through an integrated strategy combining LC-MS profiling, virtual screening, molecular docking, relative binding energy evaluation, molecular dynamics simulation, and network pharmacology, rather than solely based on previous reports. LC-MS analysis confirmed the presence of S-Rg3 and CK in red ginseng processing by-products, supporting their relevance as representative constituents. Because ginsenosides differ greatly in molecular mass, relative binding energy was introduced as an auxiliary index to reduce the potential bias of raw docking energy toward larger molecules. S-Rg3 and CK showed favorable predicted binding performance in both docking and relative binding energy evaluation, while 100 ns molecular dynamics simulations further suggested that these compounds could form dynamically stable complexes with redox- and inflammation-related targets, including Keap1, iNOS, NKIRAS2, and TLR4. These computational results should be interpreted as predictive evidence supporting the selection of S-Rg3 and CK for subsequent cellular validation, rather than direct experimental confirmation of target binding. Network pharmacology further suggested that red ginseng candidate compounds were associated with targets and pathways related to intestinal inflammatory injury, including inflammatory response, immune regulation, oxidative stress, apoptosis, autophagy, and intestinal inflammation-related signaling. Although CK was not included among the seven initially screened compounds in the network pharmacology analysis, it was detected in red ginseng processing by-products by LC-MS and showed favorable performance in relative binding energy evaluation and hub-target docking. Although CK was not included among the seven compounds obtained from the initial database-based network pharmacology screening, it was experimentally detected in the red ginseng processing by-products by LC-MS and showed favorable performance in the subsequent docking and relative binding energy analyses. In contrast, S-Rg3 was supported by both database-based candidate screening and LC-MS detection. Therefore, the final prioritization of S-Rg3 and CK was based on an integrated multi-criteria strategy combining experimental detection, network-based screening, docking performance, relative binding energy, and molecular dynamics stability rather than on a single numerical cutoff. This strategy provided a transparent rationale for selecting S-Rg3 and CK as representative candidates for subsequent cellular and multi-omics validation. Collectively, these computational and network-based results provided a rational basis for selecting S-Rg3 and CK as key candidate compounds for cellular validation.

Based on these screening results, S-Rg3 and CK were selected as representative candidate ginsenosides from red ginseng processing by-products to evaluate their protective effects against LPS-induced inflammatory epithelial injury. Consistent with previous findings on the antioxidant and anti-inflammatory activities of rare ginsenosides [15,21], 8 μM S-Rg3 and 8 μM CK showed no obvious cytotoxicity and improved the LPS-induced reduction in cell viability. Additional fixed-ratio concentration screening showed that the combined treatment remained non-cytotoxic at 8 μM S-Rg3 + 8 μM CK, whereas 16 μM + 16 μM significantly reduced cell viability, further supporting the selection of 8 μM + 8 μM as the combined working concentration. It should be noted that the abundance of Rg3 and CK in red ginseng-derived materials can vary substantially depending on processing and bioconversion conditions. In the present study, LC-MS was primarily used for qualitative identification of S-Rg3 and CK in the red ginseng processing by-products rather than for absolute quantitative determination. Therefore, the 8 μM concentrations used in the cellular experiments were selected on the basis of concentration-response and cytotoxicity screening and should be regarded as experimentally defined working concentrations rather than as direct estimates of their native abundance or intestinal exposure following consumption of red ginseng-derived materials. LPS markedly increased intracellular ROS and NO accumulation, decreased CAT and SOD activities, and increased MDA content, indicating pronounced redox imbalance and lipid peroxidation. Pretreatment with S-Rg3, CK, or their combination alleviated these changes to varying degrees. In parallel, S-Rg3 and CK restored Nrf2/HO-1 protein expression and reduced LPS-induced IL-1β, TNF-α, p-NF-κB p65, and p-IKK/IKK levels, suggesting that these compounds attenuated both oxidative stress and NF-κB-related inflammatory activation. Notably, S-Rg3 or CK alone did not induce oxidative or inflammatory injury and maintained antioxidant-related indicators at relatively high levels, suggesting that these compounds may help preserve the basal antioxidant capacity of bovine intestinal epithelial cells. The combined pretreatment showed a more pronounced protective effect than either single treatment at the selected working concentration. Fixed-ratio and matrix-based interaction analyses further supported a positive interaction between S-Rg3 and CK. Specifically, Bliss excess analysis suggested that the observed combined effect at 8 μM was higher than the Bliss-expected additive effect, while SynergyFinder+ analysis based on the normalized protective response matrix showed positive Bliss and HSA synergy scores. These findings support the use of 8 μM S-Rg3 plus 8 μM CK as the combined treatment condition. However, because the interaction analysis was based on cell viability recovery in vitro, the combined effect should be conservatively interpreted as a positive interaction trend rather than definitive pharmacological synergy.

Tight junction proteins, including ZO-1, Occludin, and Claudin-1, are essential for maintaining intestinal epithelial barrier integrity [22]. LPS-induced ROS accumulation and inflammatory activation can reduce tight junction protein expression and compromise epithelial barrier integrity, thereby aggravating intestinal epithelial injury [23]. In this study, LPS reduced the protein expression and immunofluorescence signals of ZO-1, Occludin, and Claudin-1, confirming epithelial barrier impairment in bovine small intestinal epithelial cells. Pretreatment with S-Rg3, CK, or their combination partially restored the expression and distribution of these tight junction proteins, suggesting an improvement in epithelial barrier-associated protein integrity. Together with the ROS, NO, and inflammatory cytokine results, these findings suggest that S-Rg3 and CK may preserve epithelial barrier structure by reducing oxidative stress and inflammatory responses. The combined pretreatment showed a more evident protective effect, indicating that S-Rg3 and CK may enhance the resistance of intestinal epithelial cells to subsequent inflammatory stimulation.

Transcriptomic analysis further provided a broader molecular view of the protective effects of S-Rg3 and CK. PCA showed a clear separation between the Control and LPS groups, indicating that LPS markedly altered the global gene expression profile of bovine small intestinal epithelial cells. The S-Rg3, CK, and combined pretreatment groups were also separated from the LPS group to varying degrees, suggesting that these treatments reshaped the transcriptional response induced by LPS. Consistent with the cellular inflammatory phenotypes, LPS markedly upregulated inflammation-related genes such as *CXCL8*, *CCL20,* and *IL1B*, further supporting the activation of inflammatory transcriptional programs. After S-Rg3, CK, or combined pretreatment, genes related to inflammation, cell adhesion, extracellular matrix organization, and cellular stress were altered. These transcriptomic changes were consistent with the observed reductions in inflammatory markers and the restoration of tight junction proteins, suggesting that the protective effects of S-Rg3 and CK were not limited to suppressing cytokine expression but also involved the regulation of epithelial structural integrity and cellular homeostasis.

GO and KEGG enrichment analyses showed that LPS-induced differentially expressed genes were mainly enriched in immune system regulation, cytokine production, response to lipopolysaccharide, extracellular matrix-related terms, TNF, NF-κB, IL-17, NOD-like receptor, and MAPK signaling pathways, consistent with the observed changes in NO, IL-1β, TNF-α, p-NF-κB p65, and p-IKK/IKK. Notably, the combined pretreatment group showed enrichment in pathways related to cell adhesion, integrin-mediated signaling, ECM-receptor interaction, and focal adhesion, suggesting that S-Rg3 and CK together may help maintain intestinal epithelial cell junctions and barrier-related functions. In addition to inflammatory and barrier-related pathways, transcriptomic results also highlighted changes in autophagy, lysosome, PI3K-Akt/mTOR signaling, protein processing in the endoplasmic reticulum, and apoptosis-related pathways. GSEA further showed that S-Rg3, CK, and their combined pretreatment altered pathway-level enrichment patterns associated with PI3K-Akt signaling, apoptosis, NOD-like receptor signaling, and endocytosis, supporting the involvement of cell survival, inflammatory, and vesicle transport-related processes. Machine learning analysis identified *MAP1LC3B*, *BECN1*, *CTSB*, and *TLR4* as candidate key genes, among which *MAP1LC3B*, *BECN1*, and *CTSB* are closely related to autophagosome formation, autophagy initiation, and lysosomal degradation [24]. These findings suggest that the autophagy-lysosome system may be an important regulatory node linking redox imbalance, inflammatory activation, and cell injury, and may contribute to the protective effects of S-Rg3 and CK against LPS-induced epithelial damage.

HSP70 and HSP90 are ATP-dependent molecular chaperones that cooperate in protein folding, client protein maturation, and protein quality control, thereby contributing to the maintenance of cellular proteostasis [25,26]. Reduced heat shock protein expression may weaken cellular stress adaptation and increase susceptibility to protein misfolding-related injury and apoptosis. In this study, S-Rg3 and CK restored HSP70 and HSP90 expression to varying degrees, which may contribute to improved stress adaptation and protein homeostasis under LPS stimulation. LPS also decreased p-AKT/AKT and p-mTOR/mTOR levels and increased LC3-II and P62 levels, accompanied by reduced LAMP1 and CTSB levels and weakened LysoTracker fluorescence signals. The concomitant accumulation of LC3-II and P62 does not necessarily indicate autophagy activation; instead, it may reflect impaired autophagic degradation or blockage of autophagic flux [27]. Therefore, together with the reduced lysosome-related indicators, these findings suggest that LPS induced autophagy-related protein accumulation and possible autophagy–lysosome dysfunction in bovine small intestinal epithelial cells. In parallel, LPS upregulated GRP78 and CHOP levels and increased the p-ERK/ERK ratio, indicating activation of endoplasmic reticulum stress accompanied by ERK phosphorylation [28,29]. Pretreatment with S-Rg3, CK, or their combination restored AKT/mTOR signaling, reduced the abnormal accumulation of LC3-II and P62, improved lysosome-related indicators, and decreased GRP78, CHOP, and p-ERK/ERK expression, with the combined pretreatment showing a more pronounced effect. These results suggest that S-Rg3 and CK may reduce LPS-induced cellular injury by maintaining cell survival signaling, alleviating autophagy–lysosome dysregulation, and reducing endoplasmic reticulum stress, thereby contributing to the restoration of redox-associated protein homeostasis. Importantly, the additional BafA1 inhibition experiment provided pharmacological loss-of-function evidence for the functional involvement of the autophagy–lysosome system. BafA1 treatment caused further accumulation of LC3-II and p62 and reduced lysosome-related indicators, confirming effective disruption of autophagic degradation. When BafA1 was introduced into the S-Rg3 + CK + LPS condition, the beneficial effects of the combined treatment on LC3-II, p62, CTSB, and LAMP1 were partially attenuated. Moreover, the recovery of ZO-1 expression induced by S-Rg3 and CK was also partially diminished following BafA1 treatment. These findings indicate that restoration of autophagy–lysosomal homeostasis is not merely a secondary consequence of improved cellular status, but functionally contributes to the epithelial protective effects of S-Rg3 and CK. Nevertheless, because pharmacological inhibition alone cannot establish an exclusive causal mechanism, autophagy–lysosomal restoration should be interpreted as an important contributing mechanism rather than the sole mediator of the protective effects.

Mitochondrial dysfunction is a key event in LPS-induced apoptosis. JC-1 staining showed that LPS decreased the JC-1 aggregate/monomer ratio, indicating loss of mitochondrial membrane potential [30]. MitoTracker staining further showed abnormal mitochondrial signals and morphology, while PI staining and flow cytometry revealed increased cell death and apoptosis after LPS exposure. These results indicate that oxidative stress, inflammation, and organelle dysfunction induced by LPS ultimately promote apoptosis in bovine small intestinal epithelial cells. S-Rg3, CK, and their combined pretreatment restored mitochondrial membrane potential, reduced the proportion of PI-positive cells, and decreased apoptotic rates to varying degrees, with a more evident effect in the combined pretreatment group. Western blot analysis further showed that LPS decreased the expression of the anti-apoptotic protein Bcl-2 and increased the expression of the pro-apoptotic protein Bax and cleaved-caspase-3, whereas S-Rg3, CK, and their combination reversed these apoptosis-related changes [31]. Together with the AKT/mTOR, HSP70/HSP90, autophagy-lysosome, and endoplasmic reticulum stress results, these findings suggest that S-Rg3 and CK may reduce LPS-induced apoptosis by maintaining cell survival signaling, improving protein and lysosomal homeostasis, and alleviating mitochondrial damage.

Untargeted metabolomics further revealed the regulatory effects of S-Rg3 and CK on LPS-induced cellular injury at the extracellular metabolic level. In this study, metabolomic profiling was performed using culture supernatants; therefore, the detected metabolite changes represent the extracellular metabolic profile of the conditioned medium rather than the intracellular metabolome. These extracellular metabolites may reflect the combined outcome of cellular secretion, uptake, metabolic exchange, and stress-related release under different treatment conditions. PCA, PLS-DA, and OPLS-DA showed clear separation between the extracellular metabolic profiles of the LPS and Control groups, indicating that LPS-induced cellular injury was accompanied by marked changes in the metabolite composition of the culture supernatant. Differential metabolite analysis showed that S-Rg3, CK, and their combined pretreatment altered the abundance distribution of LPS-responsive extracellular metabolites, suggesting that these treatments regulated LPS-induced cellular stress-associated metabolic exchange. KEGG enrichment analysis showed that LPS-induced extracellular metabolic changes were mainly associated with arachidonic acid metabolism, inflammatory mediator regulation of TRP channels, and the C-type lectin receptor signaling pathway, indicating that extracellular metabolic disturbance after LPS exposure was closely related to inflammatory lipid mediator production and inflammatory signaling regulation. Arachidonic acid metabolism is an important source of eicosanoids, including prostaglandins, leukotrienes, and other inflammatory lipid mediators, and dysregulation of this pathway may further amplify inflammatory responses [32,33]. Therefore, the changes in arachidonic acid metabolism-related metabolites observed in the culture supernatants were consistent with the elevated inflammatory response induced by LPS and its attenuation after S-Rg3 and CK pretreatment.

In this study, S-Rg3, CK, and their combined pretreatment affected arachidonic acid metabolism-related pathways. O2PLS analysis showed that lipid mediator-related metabolites, such as Prostaglandin E2, Leukotriene D4, and 15-deoxy-Δ12,14-prostaglandin A2, were located in high-contribution regions together with inflammation-related genes. This suggests that lipid mediator metabolism may serve as an important metabolic link through which S-Rg3 and CK regulate LPS-induced inflammatory responses. In addition to lipid mediator metabolism, arginine-related metabolism was repeatedly enriched. Arginine is an important substrate for NO synthesis, and alterations in arginine metabolism are closely associated with inflammation, oxidative stress, and cellular injury [34,35]. Differential metabolites in the S-Rg3, CK, and combined pretreatment groups were enriched in arginine biosynthesis or arginine and proline metabolism, and L-arginine was located in a high-contribution region in the combined pretreatment group in the O2PLS analysis. Together with the NO fluorescence results, which showed that LPS markedly increased intracellular NO levels while S-Rg3 and CK reduced NO signals, these findings suggest that S-Rg3 and CK may regulate the arginine/NO metabolic axis, thereby helping to alleviate LPS-induced nitrosative stress and redox imbalance.

The combined pretreatment group showed a broader metabolic regulatory profile than either single pretreatment group. Compared with the single pretreatments, differential metabolites in the S-Rg3 + CK + LPS group were more widely enriched in pathways related to the biosynthesis of amino acids, 2-oxocarboxylic acid metabolism, citrate cycle (TCA cycle), pyruvate metabolism, carbon metabolism, sulfur relay system, thiamine metabolism, and efferocytosis. These pathways are closely associated with amino acid supply, energy metabolism, redox-related metabolic processes, and apoptotic cell clearance [36,37,38]. LPS-induced inflammation and oxidative stress can increase cellular energy demand and disrupt metabolic homeostasis; therefore, the regulation of amino acid metabolism, the TCA cycle, and pyruvate metabolism by combined pretreatment may reflect metabolic remodeling that supports cellular recovery. Meanwhile, the sulfur relay system and thiamine metabolism are involved in redox metabolism and cofactor generation, suggesting that combined pretreatment may help maintain an antioxidant metabolic environment. The enrichment of efferocytosis and glycerophospholipid metabolism in the combined pretreatment group is also noteworthy. Efferocytosis is related to apoptotic cell clearance and inflammation resolution, whereas glycerophospholipid metabolism is closely associated with membrane structure, mitochondrial function, and lipid signaling [39,40]. Together with the PI staining, JC-1, flow cytometry, and apoptosis-related protein results, these metabolic changes may be associated with reduced cell death, improved mitochondrial stability, and better maintenance of membrane integrity.

Integrated transcriptomic and untargeted metabolomic analysis further supported these findings at the multi-omics level. O2PLS analysis showed that after LPS treatment, inflammation-related genes such as *IL6*, *CXCL8*, *CCL20*, and *PTGES*, together with lipid inflammatory mediator-related metabolites such as Prostaglandin E2 and Leukotriene D4, contributed strongly to the joint transcriptomic-metabolomic variation. This indicates that LPS-induced cellular injury involved coordinated inflammatory transcriptional responses and metabolic remodeling. After S-Rg3, CK, or combined pretreatment, the composition of high-contribution genes and metabolites changed, with the combined pretreatment group involving IL6, DDIT4, INHBE, CHAC1, CTH, L-arginine, and multiple lipid- and amino acid-related metabolites. Shared KEGG enrichment further showed that the combined pretreatment group was commonly enriched at both transcriptomic and metabolomic levels in pathways related to amino acid metabolism, the TCA cycle, pyruvate metabolism, arachidonic acid metabolism, the sulfur relay system, and efferocytosis. These results suggest that the protective effects of combined S-Rg3 and CK pretreatment may be associated with multi-level regulation of inflammation, energy metabolism, redox-related metabolic processes, and cell death-related processes.

In summary, this study screened and validated S-Rg3 and CK as representative ginsenosides detected in red ginseng processing by-products with protective effects against LPS-induced injury in bovine small intestinal epithelial cells. S-Rg3 and CK alleviated redox imbalance and inflammatory responses, preserved tight junction protein expression, and reduced stress-associated changes involving autophagy–lysosome dysfunction, endoplasmic reticulum stress, mitochondrial injury, and apoptosis. Transcriptomic, culture-supernatant-based untargeted metabolomic, and O2PLS-based integrative analyses further indicated that the protective effects of S-Rg3 and CK were not restricted to a single signaling pathway, but involved coordinated regulation of inflammatory transcriptional responses, extracellular metabolic remodeling, inflammatory lipid mediator metabolism, arginine/NO metabolism, amino acid and energy metabolism, and redox-related pathways. These multi-omics changes were consistent with the observed reductions in ROS and NO accumulation, inflammatory activation, barrier disruption, and stress-associated epithelial injury. Overall, these findings support the redox homeostasis–autophagy/lysosome axis as an important mechanistic framework contributing to the protective effects of S-Rg3 and CK against LPS-induced intestinal epithelial injury. This study provides mechanistic evidence that S-Rg3 and CK may act as natural modulators of endotoxin-induced redox injury, autophagy–lysosome dysfunction, and organelle stress in intestinal epithelial cells.

Several limitations should also be acknowledged. First, commercial S-Rg3 and CK standards were used in the cellular experiments to clarify the potential mechanisms of representative bioactive constituents detected in red ginseng processing by-products. Therefore, the observed effects cannot be directly equated with the biological effects of the whole by-product. Red ginseng processing by-products are chemically complex and contain multiple constituents; thus, potential interactions among different active compounds and their collective biological effects require further investigation. Second, this study was based on an in vitro bovine small intestinal epithelial cell model, which provides direct evidence for cellular protective mechanisms but cannot fully reproduce the complex intestinal microenvironment in vivo. Further studies using primary intestinal epithelial cells, intestinal organoids, or appropriate animal models are needed to verify the biological relevance of S-Rg3, CK, and red ginseng processing by-products under endotoxin-related intestinal injury conditions. In addition, although fixed-ratio and SynergyFinder+ analyses supported a positive interaction between S-Rg3 and CK, these results were based on cell viability-derived protective responses in an in vitro injury model, and further validation using additional functional endpoints is needed. Finally, although the BafA1 inhibition experiment provided pharmacological loss-of-function evidence supporting a functional contribution of autophagy–lysosomal restoration to the protective effects of S-Rg3 and CK, pharmacological inhibition alone cannot establish an exclusive causal pathway. Further studies using tandem fluorescent LC3 reporters, genetic manipulation of key autophagy–lysosome-related genes, or additional pathway-specific interventions are warranted to further clarify autophagic flux and the precise causal relationships among autophagy–lysosome function, redox homeostasis, mitochondrial injury, and apoptosis.

## 5. Conclusions

In conclusion, S-Rg3 and CK protected bovine small intestinal epithelial cells against LPS-induced injury by restoring redox homeostasis, suppressing inflammatory activation, preserving tight junction protein expression, and alleviating autophagy–lysosome dysfunction, mitochondrial injury, and apoptosis. Integrated transcriptomic and metabolomic analyses further linked these effects to redox-related and metabolic pathways. These findings support the potential of red ginseng-derived ginsenosides as bioactive candidates for improving intestinal epithelial health in young ruminants, although further in vivo validation is required.

## Figures and Tables

**Table 2:** Major predicted interactions of S-Rg3 and Compound K with selected target proteins based on molecular docking analysis.

Compound	Target	Interacting Residues	Interaction Type
S-Rg3	iNOS	Tyr367, Asn364	Conventional hydrogen bond
S-Rg3	iNOS	Pro344, Val346	Alkyl
S-Rg3	Keap1	Cys368, Val465, Val418, Gly419, Val369, Val608, Arg326	Conventional hydrogen bond
S-Rg3	Keap1	Cys513, Ala466, Val420, Val467, Ala366	Alkyl
S-Rg3	NKIRAS2	Gln136, Val144, Lys145, Tyr163, Lys167, Gln170, Glu112, Thr114	Conventional hydrogen bond
S-Rg3	NKIRAS2	His137, Lys140	Alkyl/Pi-alkyl
S-Rg3	TLR4	Asn529, Asn576, Thr605, Asn575, Cys606, Ala607	Conventional hydrogen bond
S-Rg3	TLR4	Lys503, His527	Pi-alkyl
CK	iNOS	Asp376, Cys194, Asn364	Conventional hydrogen bond
CK	iNOS	Trp188	Pi-sigma
CK	iNOS	Pro192, Ala191, Arg193, Tyr485, Pro344, Met349, Glu371, Val346	Alkyl/Pi-alkyl
CK	Keap1	Val606, Gly367, Val465, Val512	Conventional hydrogen bond
CK	Keap1	Val514, Val369, Cys513, Ala607, Val561	Alkyl
CK	NKIRAS2	Ser33, Glu34, Ser19, Ile36	Conventional hydrogen bond
CK	NKIRAS2	Val151, Lys121, Leu124, Met35	Alkyl
CK	TLR4	Tyr375, Asp377, Arg380	Conventional hydrogen bond
CK	TLR4	Ile356	Alkyl/Pi-alkyl
CK	TLR4	Thr355	Carbon hydrogen bond

Note: Interaction types were assigned according to the two-dimensional interaction diagrams generated using BIOVIA Discovery Studio Visualizer 2021. Only the major, explicitly identified interactions are summarized here; van der Waals contacts and unfavorable interactions are not listed.

## Data Availability

The RNA-sequencing data generated in this study have been deposited in the NCBI Sequence Read Archive (SRA) under BioProject accession PRJNA1522803. The dataset is currently subject to a four-year embargo period because it forms part of an ongoing research project and will be used in subsequent related studies. Therefore, the sequencing data are not publicly accessible at present. The data will be made publicly available through the NCBI SRA upon expiration of the embargo period. Other data supporting the findings of this study are available from the corresponding author upon reasonable request. The datasets presented in this study are available from the corresponding author on reasonable request.

## Supplementary Materials

The following supporting information can be downloaded at: https://www.mdpi.com/article/10.3390/antiox15101290/s1, Supplementary Methods S1: Network pharmacology analysis; Supplementary Methods S2: Molecular docking and relative binding energy calculation; Supplementary Methods S3: Molecular dynamics simulation; Supplementary Methods S4: Fixed-ratio combination assay and Bliss interaction analysis; Figure S1: Molecular docking screening of candidate ginsenosides against selected target proteins; Figure S2: Network pharmacology analysis of red ginseng-derived compounds against calf diarrhea-related intestinal inflammatory injury; Figure S3: Fixed-ratio combination viability and interaction analysis of S-Rg3 and CK in bovine intestinal epithelial cells; Figure S4: Transcriptomic profiling and exploratory machine-learning analysis of LPS-treated bovine intestinal epithelial cells following S-Rg3 and CK pretreatment; Figure S5: KEGG enrichment analysis of upregulated and downregulated differentially expressed genes; Figure S6: Exploratory machine-learning analysis of candidate feature genes; Figure S7: PLS-DA and OPLS-DA analyses of untargeted metabolomic profiles; Figure S8: Untargeted metabolomic profiling of bovine small intestinal epithelial cells following LPS challenge and S-Rg3/CK pretreatment; Figure S9: KEGG enrichment analysis of upregulated and downregulated differential metabolites; Figure S10: Integrated transcriptomic–metabolomic analysis of LPS-treated bovine small intestinal epithelial cells following S-Rg3 and CK pretreatment; Table S1: Information on ginsenoside reference standards used for LC-MS identification; Table S2: Antibodies and staining reagents used in this study; Table S3: Candidate active compounds of red ginseng retrieved from TCMSP.
