# Supplementary material for: Red Ginseng-Derived 20(S)-Ginsenoside Rg3 and Compound K Restore Redox Homeostasis and Alleviate LPS-Induced Barrier Damage in Bovine Intestinal Epithelial Cells
Source: Antioxidants (Basel). 2026 Oct 4;15(10):1290. doi: 10.3390/antiox15101290 (PMC13700754; doi:10.3390/antiox15101290)
Supplement: Supplementary file 1 [file antioxidants-15-01290-s001.zip › Sfigure.pdf]

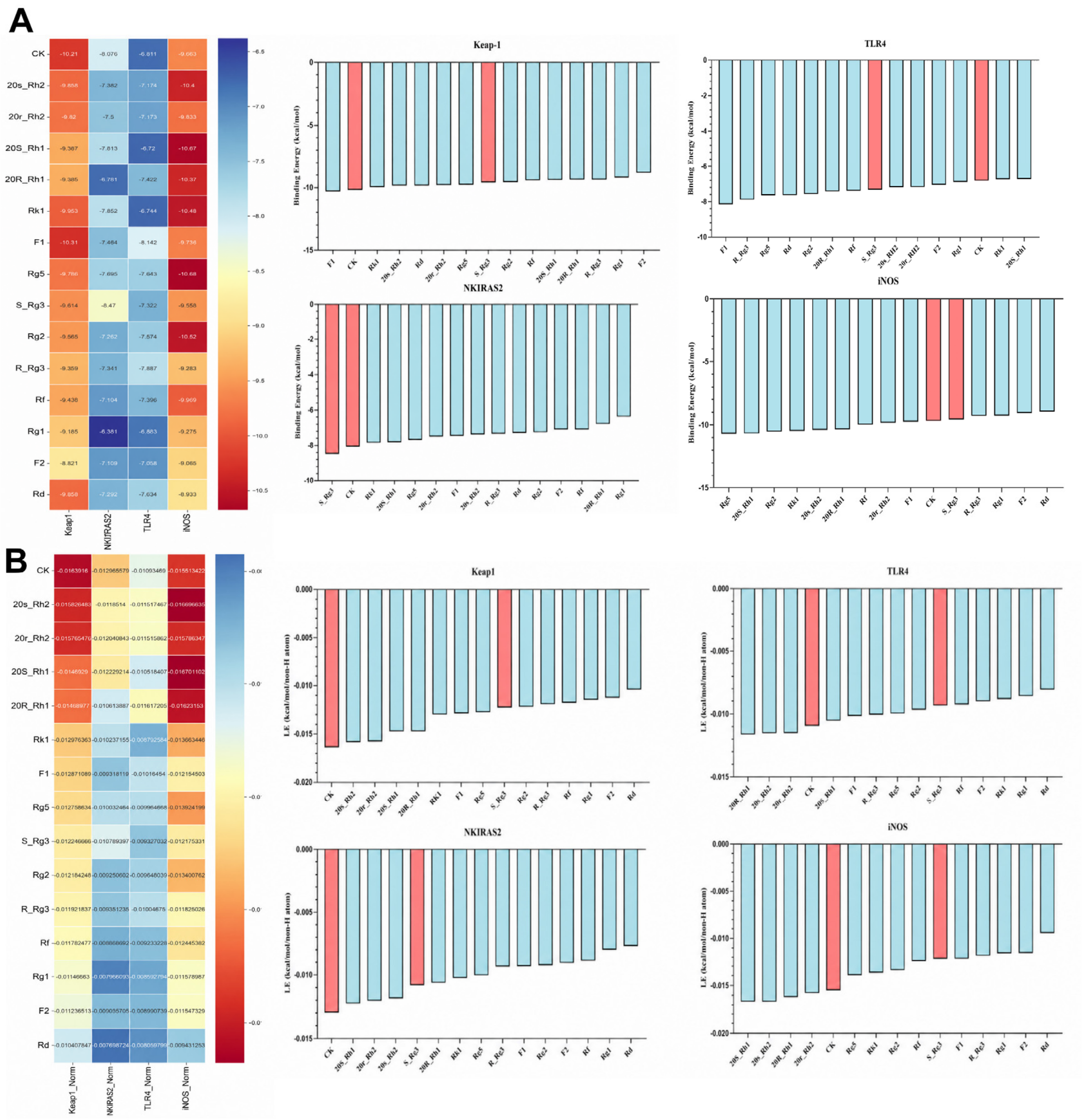

Figure S1. Molecular docking screening of candidate ginsenosides against selected target proteins.(A) Predicted binding energies of candidate ginsenosides with Keap1, TLR4, NKIRAS2, and iNOS, shown as a heatmap and ranked bar plots. (B) Molecular-weight-normalized relative binding energy (RBE) values for the same ligand–target pairs. S-Rg3 and Compound K (CK) are highlighted in the ranked plots. More negative values indicate more favorable predicted interactions.

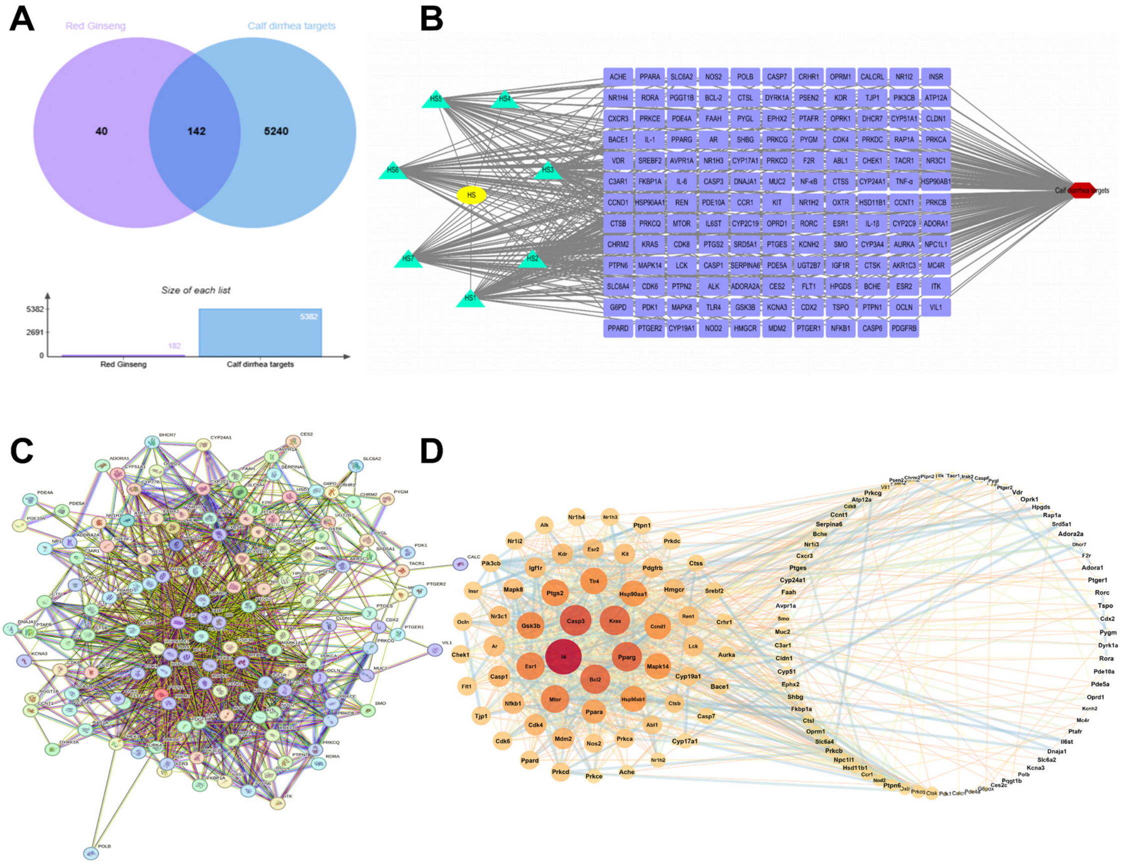

Figure S2. Network pharmacology analysis of red ginseng-derived compounds against calf diarrhea-related intestinal inflammatory injury.(A) Venn diagram showing the overlap between red ginseng-related targets and calf diarrhea-related targets. (B) Compound–target–disease network of the overlapping targets. (C) Protein–protein interaction (PPI) network of the shared targets. (D) Core-target network based on topological analysis of the PPI network.

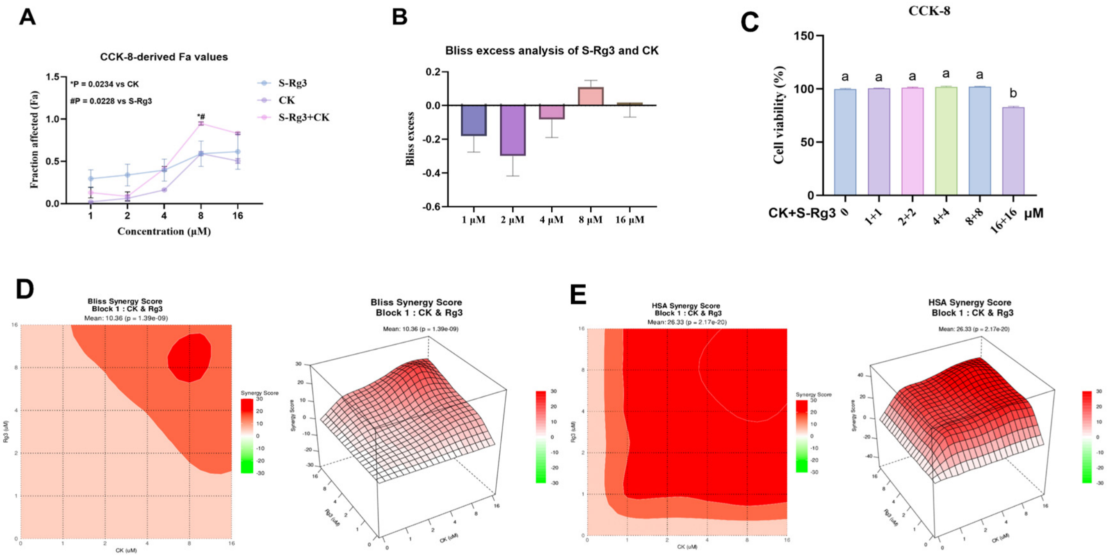

Figure S3. Fixed-ratio combination viability and interaction analysis of S-Rg3 and CK in bovine intestinal epithelial cells.(A) CCK-8-derived fraction affected (Fa) values for S-Rg3, CK, and their 1:1 combination at different concentrations under LPS-induced injury conditions. (B) Bliss excess values for the S-Rg3 and CK combination at different concentrations; positive values indicate an observed combined effect greater than the Bliss-expected additive effect. (C) Cell viability following treatment with S-Rg3 and CK at fixed 1:1 combinations of 1+1, 2+2, 4+4, 8+8, and 16+16 μM for 24 h. (D) Bliss interaction landscape shown as two-dimensional and three-dimensional synergy maps. (E) Highest single agent (HSA) interaction landscape shown as two-dimensional and three-dimensional synergy maps. Data are presented as mean ± SEM. In (A), P = 0.0234 versus CK and #P = 0.0228 versus S-Rg3 at the indicated concentration. In (C), different lowercase letters indicate significant differences among groups ($P < 0.05$).

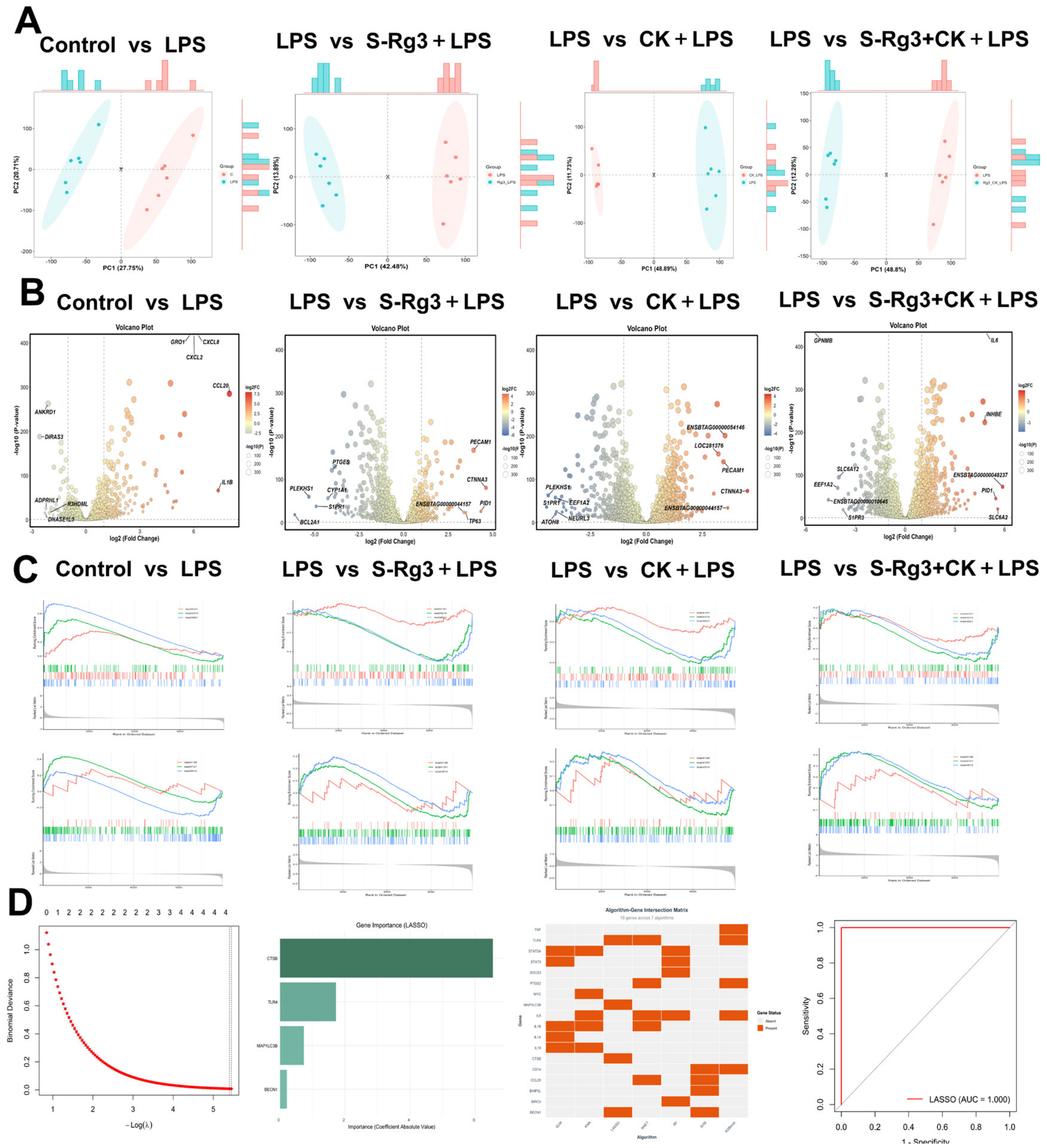

Figure S4. Transcriptomic profiling and exploratory machine-learning analysis of LPS-treated bovine intestinal epithelial cells following S-Rg3 and CK pretreatment.

(A) Principal component analysis (PCA) of the indicated pairwise comparisons. (B) Volcano plots showing differentially expressed genes. (C) Gene set enrichment analysis (GSEA) of representative pathways. (D) Exploratory machine-learning analysis, including LASSO regression, gene-importance ranking, algorithm–gene intersection analysis, and receiver operating characteristic (ROC) evaluation.

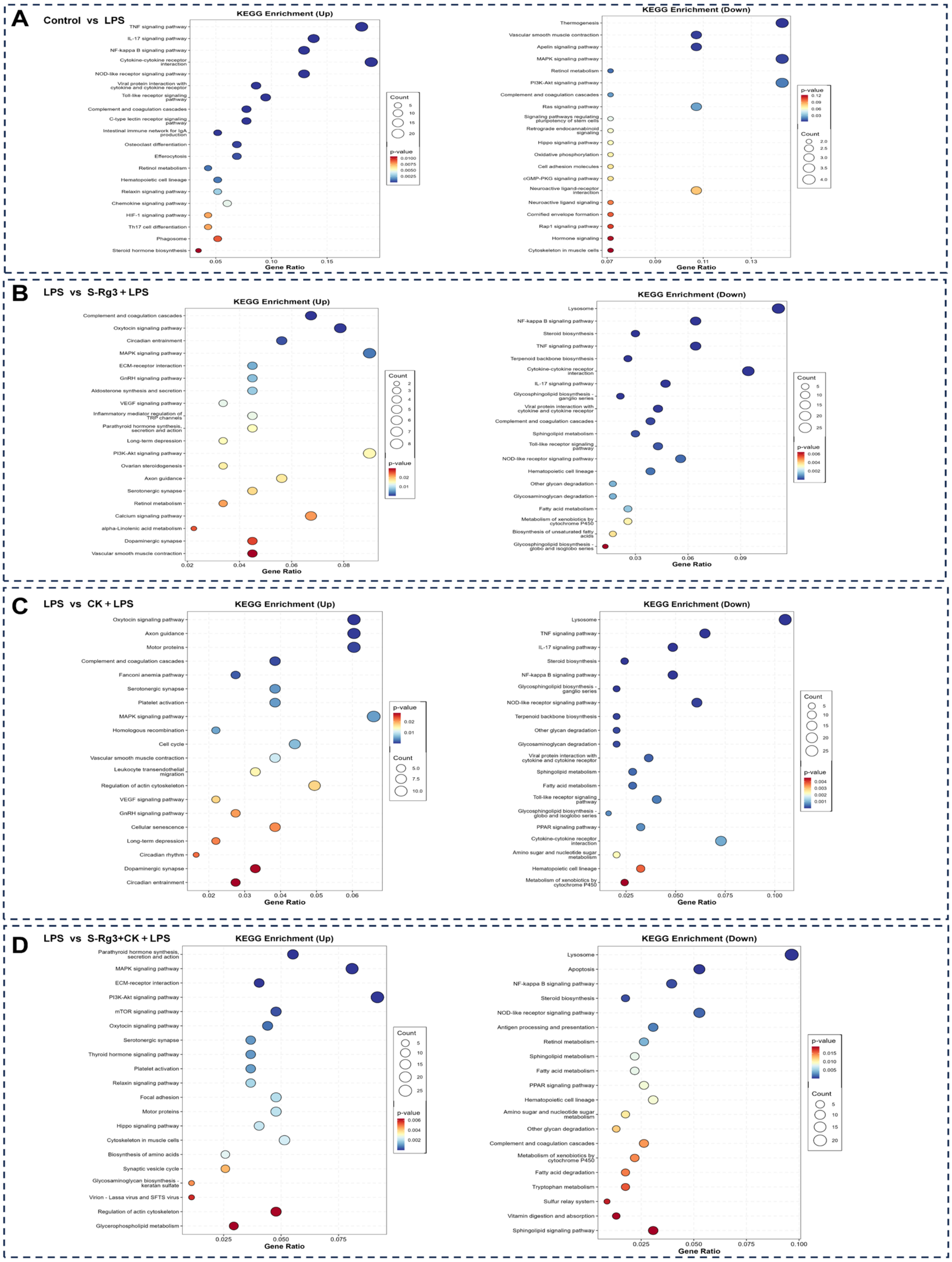

Figure S5. KEGG enrichment analysis of upregulated and downregulated differentially expressed genes. KEGG pathway enrichment analysis was performed separately for upregulated and downregulated DEGs to further characterize pathway changes under different treatment conditions. (A) Control vs LPS. (B) LPS vs S-Rg3+LPS. (C) LPS vs CK+LPS. (D) LPS vs S-Rg3+CK+LPS. Bubble size represents the number of enriched genes, and bubble color represents enrichment significance.

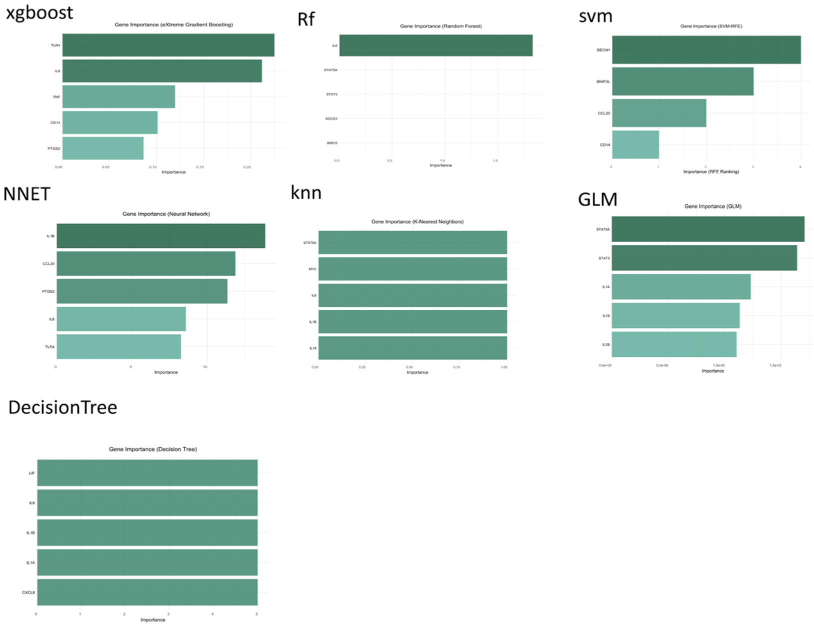

Figure S6. Exploratory machine-learning analysis of candidate feature genes. Multiple machine-learning algorithms were used to evaluate the relative importance of candidate genes selected by LASSO regression. Feature importance was assessed using XGBoost, RF, SVM, ANN, KNN, GLM, and decision tree models. These analyses were used to examine the consistency of candidate gene importance across different algorithms rather than to establish a predictive biomarker model. XGBoost, extreme gradient boosting; RF, random forest; SVM, support vector machine; ANN, artificial neural network; KNN, k-nearest neighbors; GLM, generalized linear model.

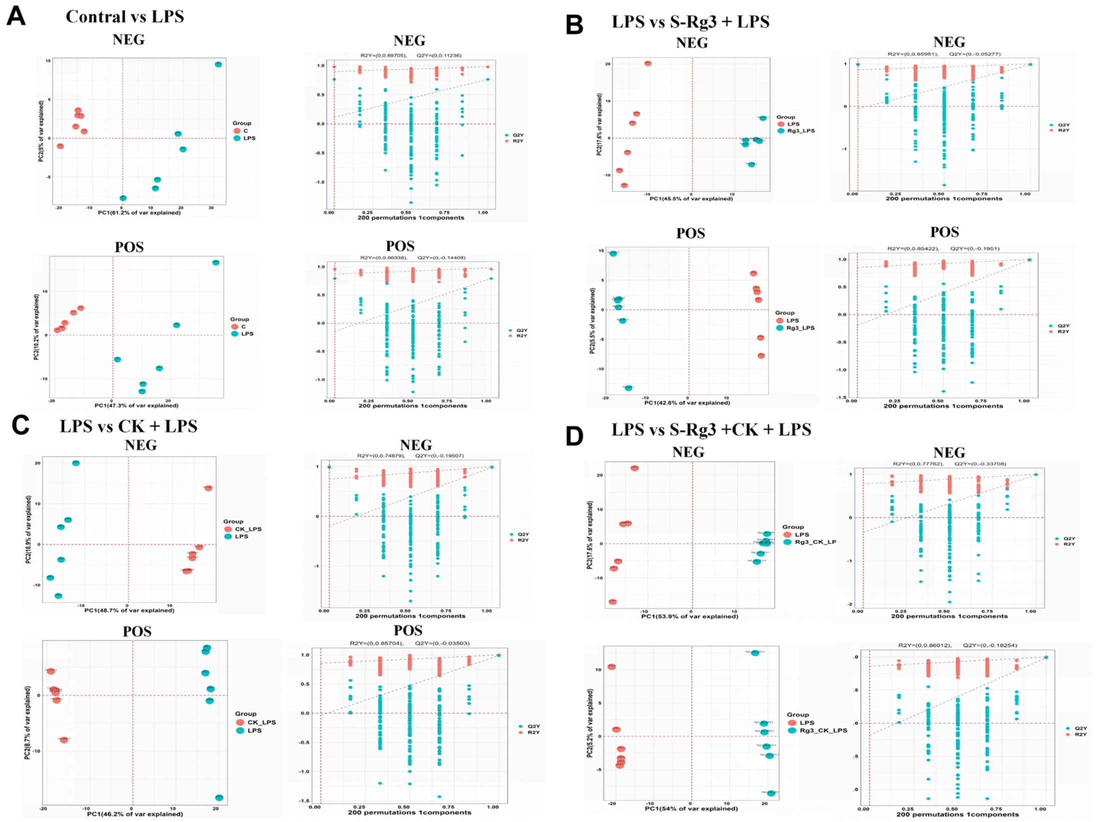

Figure S7. PLS-DA and OPLS-DA analyses of untargeted metabolomic profiles.PLS-DA and OPLS-DA score plots were generated to evaluate the separation trends of untargeted metabolomic profiles among different comparison groups under negative ionization mode (NEG) and positive ionization mode (POS). The comparisons included Control vs LPS, LPS vs S-Rg3+LPS, LPS vs CK+LPS, and LPS vs S-Rg3+CK+LPS. Each point represents one biological sample.

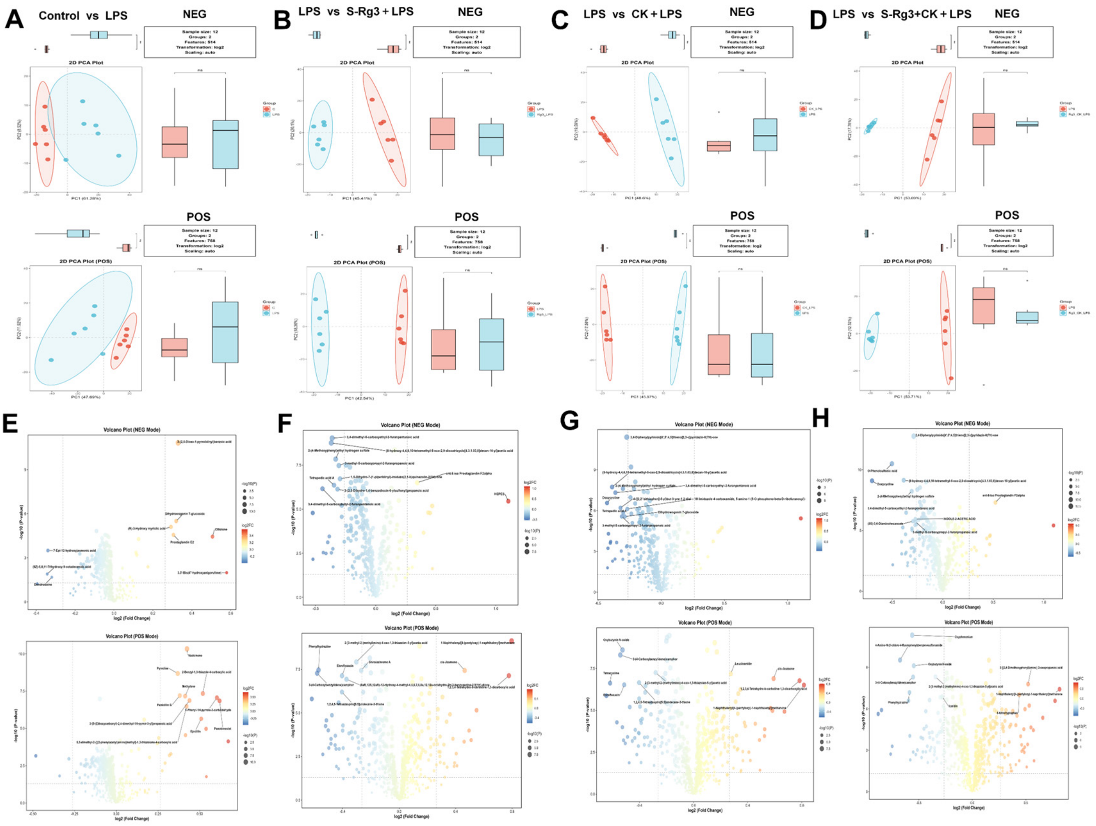

Figure S8. Untargeted metabolomic profiling of bovine small intestinal epithelial cells following LPS challenge and S-Rg3/CK pretreatment.(A–D) Principal component analysis (PCA) of Control vs LPS, LPS vs S-Rg3+LPS, LPS vs CK+LPS, and LPS vs S-Rg3+CK+LPS under negative (NEG) and positive (POS) ionization modes. (E–H) Volcano plots showing differential metabolites for the corresponding comparisons under NEG and POS modes. NEG, negative ionization mode; POS, positive ionization mode.

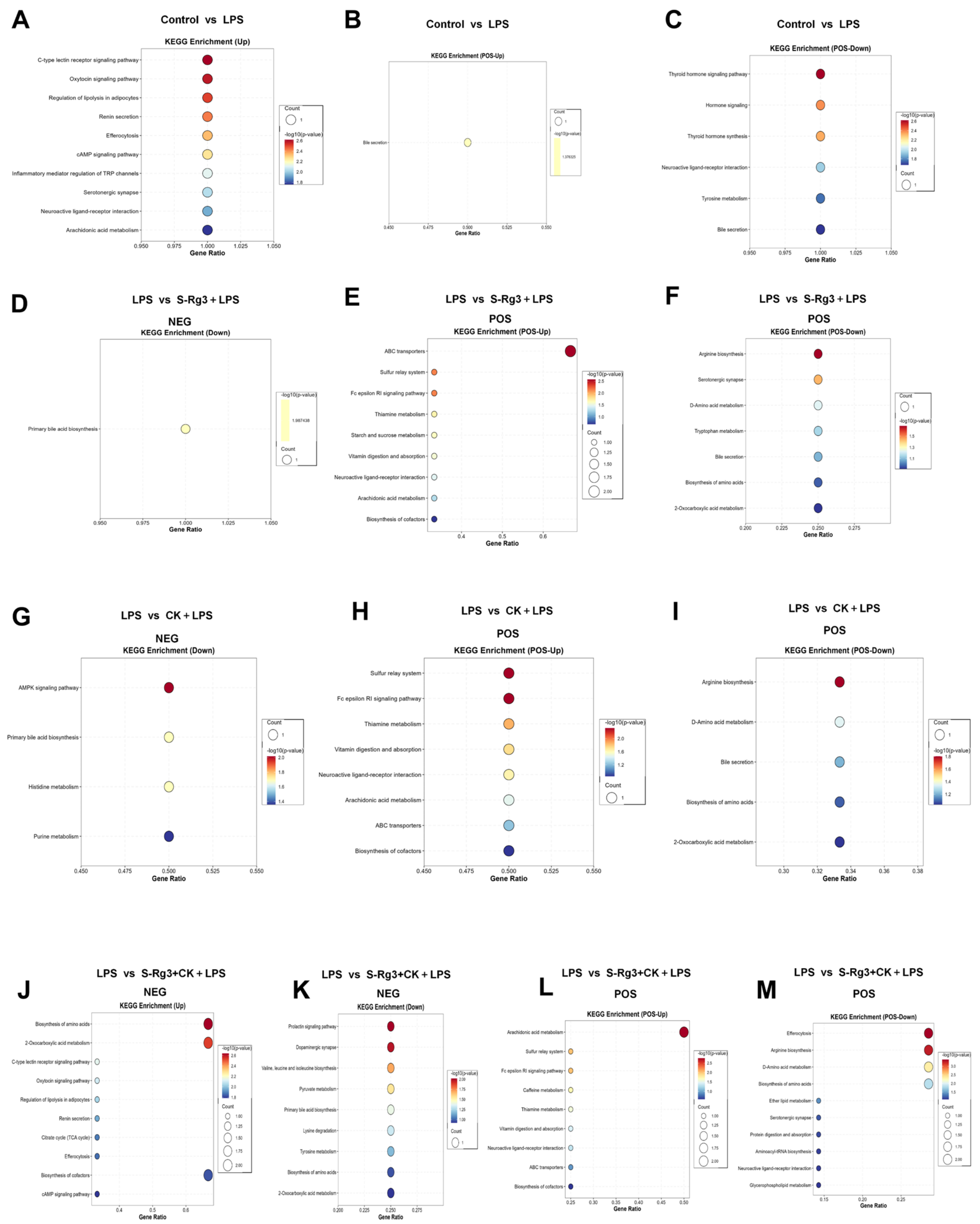

Figure S9. KEGG enrichment analysis of upregulated and downregulated differential metabolites. KEGG pathway enrichment analysis was performed for differential metabolites under negative (NEG) and positive (POS) ionization modes. (A–C) Control vs LPS. (D–F) LPS vs S-Rg3+LPS. (G–I) LPS vs CK+LPS. (J–M) LPS vs S-Rg3+CK+LPS. Bubble size represents the number of enriched metabolites, and bubble color represents enrichment significance.

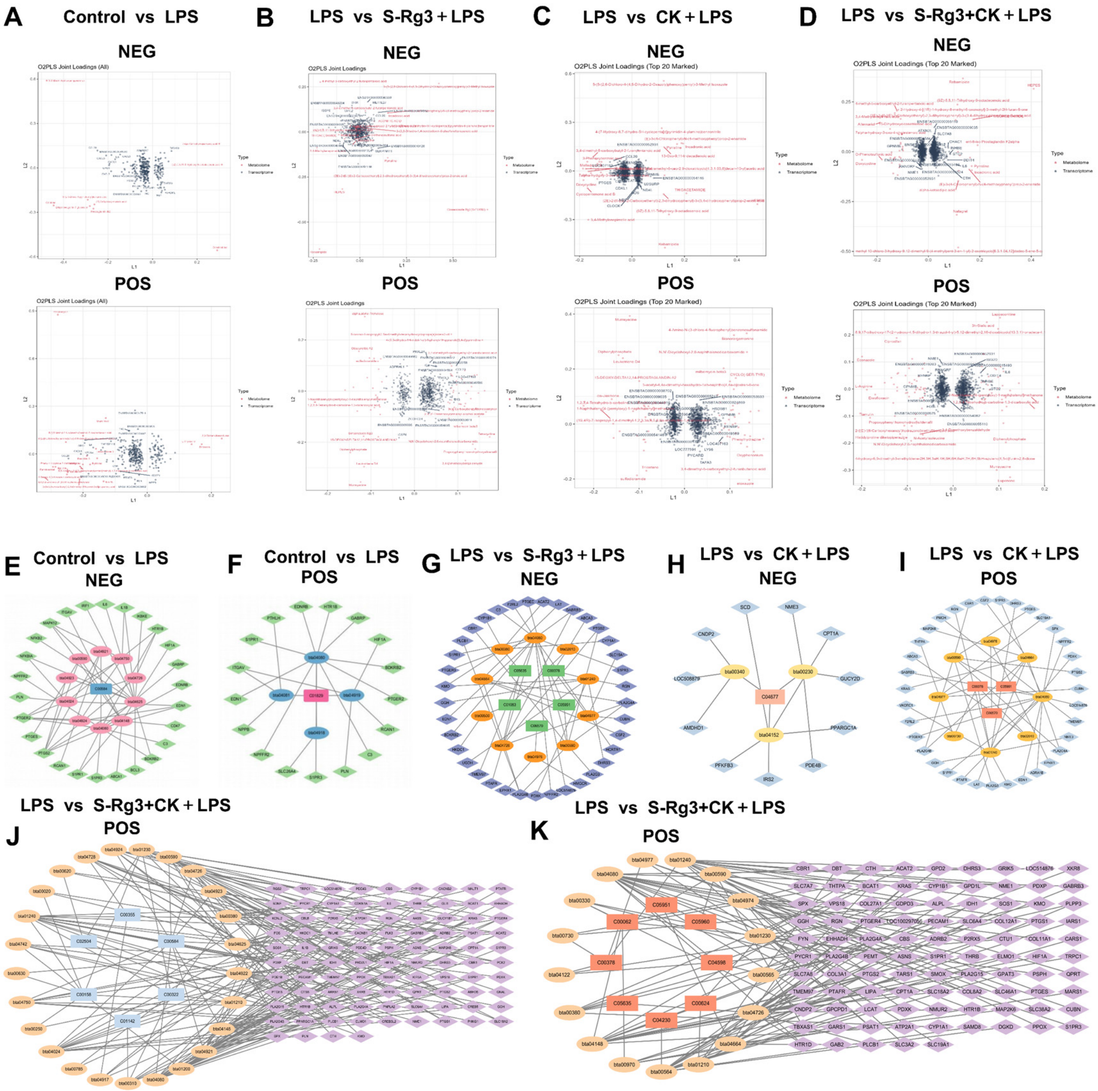

Figure S10. Integrated transcriptomic–metabolomic analysis of LPS-treated bovine small intestinal epithelial cells following S-Rg3 and CK pretreatment.(A–D) O2PLS joint loading plots for Control vs LPS, LPS vs S-Rg3+LPS, LPS vs CK+LPS, and LPS vs S-Rg3+CK+LPS under negative (NEG) and positive (POS) ionization modes. (E–K) Representative pathway–gene–metabolite interaction networks for the corresponding comparisons. NEG, negative ionization mode; POS, positive ionization mode; O2PLS, two-way orthogonal partial least squares.
