# Supplementary material for: Red Ginseng-Derived 20(S)-Ginsenoside Rg3 and Compound K Restore Redox Homeostasis and Alleviate LPS-Induced Barrier Damage in Bovine Intestinal Epithelial Cells
Source: Antioxidants (Basel). 2026 Oct 4;15(10):1290. doi: 10.3390/antiox15101290 (PMC13700754; doi:10.3390/antiox15101290)
Supplement: Supplementary file 1 [file antioxidants-15-01290-s001.zip › STable.pdf]

Table S1. Information on ginsenoside reference standards used for LC-MS identification

No.	Reference standard	Molecular formula	Supplier
1	Ginsenoside Rg1	$C_{42}H_{72}O_{14}$	Refens Biotechnology Co., Ltd. (China)
2	Ginsenoside Rf	$C_{42}H_{72}O_{14}$	Refens Biotechnology Co., Ltd. (China)
3	Ginsenoside Rg2	$C_{42}H_{72}O_{13}$	Refens Biotechnology Co., Ltd. (China)
4	Ginsenoside Ro	$C_{48}H_{76}O_{19}$	Refens Biotechnology Co., Ltd. (China)
5	(S)Ginsenoside Rh1	$C_{36}H_{62}O_{9}$	Refens Biotechnology Co., Ltd. (China)
6	(R)Ginsenoside Rh1	$C_{36}H_{62}O_{9}$	Refens Biotechnology Co., Ltd. (China)
7	Ginsenoside Rd	$C_{48}H_{82}O_{18}$	Refens Biotechnology Co., Ltd. (China)
8	(R)Ginsenoside Rg2	$C_{42}H_{72}O_{13}$	Refens Biotechnology Co., Ltd. (China)
9	Ginsenoside F1	$C_{36}H_{62}O_{9}$	Refens Biotechnology Co., Ltd. (China)
10	Ginsenoside F2	$C_{42}H_{72}O_{13}$	Refens Biotechnology Co., Ltd. (China)
11	Ginsenoside F4	$C_{42}H_{70}O_{12}$	Refens Biotechnology Co., Ltd. (China)
12	Ginsenoside Ra2	$C_{58}H_{98}O_{26}$	Refens Biotechnology Co., Ltd. (China)
13	Ginsenoside Rg6	$C_{42}H_{70}O_{12}$	Refens Biotechnology Co., Ltd. (China)
14	Ginsenoside Rk3	$C_{36}H_{60}O_{8}$	Refens Biotechnology Co., Ltd. (China)
15	Ginsenoside Rs2	$C_{55}H_{92}O_{23}$	Refens Biotechnology Co., Ltd. (China)
16	Ginsenoside Rh4	$C_{36}H_{60}O_{8}$	Refens Biotechnology Co., Ltd. (China)
17	(S)Ginsenoside Rg3	$C_{42}H_{72}O_{13}$	Refens Biotechnology Co., Ltd. (China)
18	(R)Ginsenoside Rg3	$C_{42}H_{72}O_{13}$	Refens Biotechnology Co., Ltd. (China)
19	Compound K	$C_{36}H_{62}O_{8}$	Refens Biotechnology Co., Ltd. (China)
20	Ginsenoside Rk1	$C_{42}H_{70}O_{12}$	Refens Biotechnology Co., Ltd. (China)
21	Ginsenoside Rg5	$C_{42}H_{70}O_{12}$	Refens Biotechnology Co., Ltd. (China)
22	(S)Ginsenoside Rh2	$C_{36}H_{62}O_{8}$	Refens Biotechnology Co., Ltd. (China)
23	(R)Ginsenoside Rh2	$C_{36}H_{62}O_{8}$	Refens Biotechnology Co., Ltd. (China)

24	Ginsenoside Rs5 or isomer	C44H72O13	Refens Biotechnology Co., Ltd. (China)

Note: All reference standards were purchased from Refens Biotechnology Co., Ltd. (China). LC-MS, liquid chromatography-mass spectrometry

Table S2. Antibodies and staining reagents used in this study

Application	Target/Reagent	Supplier	Dilution
Western blot	Nrf2	Shanben Biotech, China	1:1500
Western blot	HO-1	Shanben Biotech, China	1:1500
Western blot	Cleaved caspase-3	Shanben Biotech, China	1:1500
Western blot	HSP70	Bioss, Beijing, China	1:2000
Western blot	HSP90	Bioss, Beijing, China	1:2000
Western blot	p-AKT	Bioss, Beijing, China	1:2000
Western blot	AKT	Bioss, Beijing, China	1:1000
Western blot	Bcl-2	Bioss, Beijing, China	1:2000
Western blot	Bax	Bioss, Beijing, China	1:2000
Western blot	IL-1β	CST, USA	1:2000
Western blot	TNF-α	CST, USA	1:2000
Western blot	NF-κB p65	CST, USA	1:2000
Western blot	p-NF-κB p65	CST, USA	1:2000
Western blot	p-IKK	CST, USA	1:2000
Western blot	IKK	CST, USA	1:2000
Western blot	LC3	CST, USA	1:1000
Western blot	P62	CST, USA	1:2500
Western blot	CHOP	CST, USA	1:2500
Western blot	GRP78	CST, USA	1:2500
Western blot	p-ERK	CST, USA	1:2500
Western blot	ERK	CST, USA	1:2500
Western blot	Occludin	Proteintech, Wuhan, China	1:2000

Western blot	Claudin-1	Proteintech, Wuhan, China	1:2000
Western blot	ZO-1	Proteintech, Wuhan, China	1:2000
Western blot	GAPDH		
Immunofluorescence	IL-1β	YuanTai Biotech, China	1:250
Immunofluorescence	TNF-α	YuanTai Biotech, China	1:250
Immunofluorescence	Occludin	Proteintech, Wuhan, China	1:250
Immunofluorescence	Claudin-1	Proteintech, Wuhan, China	1:250
Immunofluorescence	ZO-1	Proteintech, Wuhan, China	1:250
Fluorescence staining	Phalloidin	Biosharp, China	1:100

Table S3. Candidate active compounds of red ginseng retrieved from TCMSP

Ranking name	Mol ID	Molecule name
HS1	MOL012329	20(S)-Ginsenoside Rg3
HS2	MOL012335	20(R)-Ginsenoside Rg3
HS3	MOL002032	DNOP
HS4	MOL002372	(6Z,10E,14E,18E)-2,6,10,15,19,23-Hexamethyltetracosa-2,6,10,14,18,22-hexaene
HS5	MOL000358	β-Sitosterol
HS6	MOL005332	(3R,5R,8R,9R,10R,12R,13R,14R,17S)-17-[(2S)-2-Hydroxy-6-methylhept-5-en-2-yl]-4,4,8,10,14-pentamethyl-2,3,5,6,7,9,11,12,13,15,16,17-dodecahydro-1H-cyclopenta[a]phenanthrene-3,12-diol
HS7	MOL005334	(3S,5R,8R,9R,10R,12R,13R,14R,17S)-17-[(2S)-2-Hydroxy-6-methylhept-5-en-2-yl]-4,4,8,10,14-pentamethyl-2,3,5,6,7,9,11,12,13,15,16,17-dodecahydro-1H-cyclopenta[a]phenanthrene-3,12-diol

Note: The original TCMSP annotation for MOL012335 is “ginsenoside Rg3_qt”. It is presented here as 20(R)-Ginsenoside Rg3 to distinguish it from 20(S)-Ginsenoside Rg3.
