# Supplementary material for: Red Ginseng-Derived 20(S)-Ginsenoside Rg3 and Compound K Restore Redox Homeostasis and Alleviate LPS-Induced Barrier Damage in Bovine Intestinal Epithelial Cells
Source: Antioxidants (Basel). 2026 Oct 4;15(10):1290. doi: 10.3390/antiox15101290 (PMC13700754; doi:10.3390/antiox15101290)
Supplement: Supplementary file 1 [file antioxidants-15-01290-s001.zip › Supplementary Methods.pdf]

Supplementary Methods S1. Network pharmacology analysis

Considering that human targets are more comprehensively annotated in currently available drug-target prediction databases, Homo sapiens was used as the reference species for target prediction in this study. The predicted targets were used only for preliminary screening of conserved targets and signaling pathways.

Chemical constituents related to red ginseng were retrieved from the Traditional Chinese Medicine Systems Pharmacology Database and Analysis Platform (TCMSP) using “Red Ginseng” as the keyword. Candidate active compounds were screened according to commonly used pharmacokinetic criteria in network pharmacology studies, including oral bioavailability (OB) $\geq$ 30%, drug-likeness (DL) $\geq$ 0.18, and Caco-2 permeability ≥ -0.02.

The standardized names or two-dimensional structures of the screened compounds were submitted to SwissTargetPrediction and PharmMapper for potential target prediction, with the species set as **Homo sapiens**. Targets predicted by the two databases were integrated and deduplicated to obtain the potential target set of red ginseng candidate compounds. Disease-related targets were collected from GeneCards and DisGeNET using “Neonatal Calf Diarrhea”, “Calf Diarrhea”, and “Diarrhea” as keywords. To improve disease relevance, genes with relevance scores higher than the median value in each database were retained, and targets from different databases were merged and deduplicated to construct the calf diarrhea-related disease target set.

The compound-related targets and calf diarrhea-related disease targets were imported into the Bioinformatics online platform to identify overlapping targets. These overlapping targets were considered potential targets through which red ginseng candidate compounds may be involved in calf diarrhea-related intestinal inflammatory injury. A compound-target-disease network was then constructed and visualized using Cytoscape software (version 3.9.1).

For protein-protein interaction (PPI) analysis, the overlapping targets were submitted to the STRING database (version 11.5), with the species set as Homo sapiens

and the minimum required interaction confidence score set to > 0.7. The resulting network was imported into Cytoscape for visualization and topological analysis. Degree, betweenness centrality, and closeness centrality were calculated using the NetworkAnalyzer tool. Targets with values higher than the corresponding median values for all three parameters were defined as hub targets.

Gene Ontology (GO) functional enrichment analysis and Kyoto Encyclopedia of Genes and Genomes (KEGG) pathway enrichment analysis of the overlapping targets were performed using the Bioinformatics online platform. GO terms were classified into biological process (BP), cellular component (CC), and molecular function (MF). The enrichment results were used to predict the biological processes and signaling pathways potentially involved in the effects of red ginseng candidate compounds on calf diarrhea-related intestinal inflammatory injury.

Supplementary Methods S2. Molecular docking and relative binding energy calculation

The two- or three-dimensional structures of candidate compounds were obtained from the PubChem database and energy-minimized before molecular docking. The crystal structures of target proteins were downloaded from the RCSB Protein Data Bank. Before docking, receptor proteins were prepared by removing water molecules and original ligands, adding hydrogen atoms, assigning charges, and converting them into the required docking format. Ligand structures were optimized and saved in the corresponding format for docking analysis.

Docking grids were generated according to the active pocket or the original ligand-binding region of each target protein to ensure that the main binding site was covered. Docking scores were expressed as binding energies, with lower binding energies indicating stronger predicted ligand-receptor binding stability. Docked complexes with favorable binding energies and reasonable conformations were selected for further interaction analysis.

The ligand-receptor interaction patterns were visualized using molecular

visualization software. Non-covalent interactions, including hydrogen bonds, hydrophobic interactions, van der Waals forces, and alkyl interactions, were analyzed to evaluate the binding characteristics between candidate compounds and target proteins.

Considering the differences in molecular weight among ginsenosides, relative binding energy was used as an auxiliary screening index to reduce potential bias caused by molecular size. In this calculation, the binding energy referred to the absolute value of the docking binding energy, and the relative molecular mass referred to the molecular weight of the corresponding compound. A higher relative binding energy indicated stronger predicted binding ability per unit molecular mass.

Supplementary Methods S3. Molecular dynamics simulation

All-atom molecular dynamics simulations were performed using AMBER 24 according to previously reported protocols. Proteins were parameterized using the Amber14SB force field, and water molecules were described using the TIP3P water model. Each ligand-receptor complex was placed in a truncated octahedral water box, with a minimum distance of 10.0 Å between the complex and the box boundary. Na^+ and Cl^- ions were added to neutralize the system and maintain an ionic strength of 0.15 M.

Before production simulation, each system was subjected to 10,000 steps of energy minimization, followed by 100 ps of heating and equilibration under the NVT ensemble and 1 ns of equilibration under the NPT ensemble. After equilibration, a 100 ns unrestrained MD simulation was performed under the NPT ensemble at 300 K and 1 bar. Temperature and pressure were controlled using a Langevin thermostat and a Berendsen barostat, respectively. Covalent bonds involving hydrogen atoms were constrained using the SHAKE algorithm, and the integration time step was set to 2 fs.

Trajectory analyses were performed using the CPPTRAJ module. RMSD, RMSF, Rg, SASA, and the number of hydrogen bonds were calculated to evaluate the conformational stability of the complexes. Binding free energies were calculated using

the MM-PBSA method, and representative conformations were extracted from the stable phase of the simulation for energy evaluation. Results are presented as mean ± SD.

Supplementary Methods S4. Fixed-ratio combination assay and Bliss interaction analysis

To further evaluate the combined effect of S-Rg3 and CK, additional fixed-ratio and matrix-based combination assays were performed using CCK-8-derived cell viability recovery as the endpoint. These assays were conducted under the same LPS-induced injury conditions used in the main cellular experiments. For the 1:1 fixed-ratio assay, cells were pretreated with S-Rg3 alone, CK alone, or S-Rg3 plus CK at 1, 2, 4, 8, and 16 μM for 24 h, followed by stimulation with 50 mg/L LPS for another 24 h. Cell viability was measured using the CCK-8 assay.

The fraction affected (Fa) was calculated based on cell viability recovery relative to the Control and LPS groups using the following formula: Fa = (Viability_treatment − Viability_LPS)/(Viability_control − Viability_LPS). The effects of treatment, concentration, and their interaction on Fa values were analyzed using two-way ANOVA followed by Tukey's multiple comparisons test.

Bliss independence analysis was performed as a descriptive interaction analysis based on CCK-8-derived Fa values. The Bliss-expected effect was calculated as E_Bliss = E_S-Rg3 + E_CK − E_S-Rg3 × E_CK, where E_S-Rg3 and E_CK represent the Fa values of S-Rg3 and CK single treatments at the corresponding concentrations, respectively. Bliss excess was calculated as E_observed − E_Bliss, where E_observed represents the Fa value of the combined treatment. A positive Bliss excess value was interpreted as an observed combined effect higher than the Bliss-expected additive effect, whereas values close to 0 indicated an approximately additive effect.

For matrix-based interaction analysis, cells were pretreated with S-Rg3 and CK in a 6 × 6 concentration matrix at 0, 1, 2, 4, 8, and 16 μM under LPS-induced injury conditions. After 24 h of drug pretreatment, cells were stimulated with 50 mg/L LPS

for another 24 h, and cell viability was measured using the CCK-8 assay. The CCK-8-derived cell viability values were normalized to protective response values relative to the Control and LPS groups using the following formula: Protective response (%) = (Viability_treatment − Viability_LPS)/(Viability_control − Viability_LPS) × 100. The normalized protective response matrix was then analyzed using the SynergyFinder+ web application (version 3.0; https://synergyfinder.org/ and https://synergyfinderplus.org/) to calculate synergy scores based on multiple reference models, including ZIP, HSA, Bliss, and Loewe. Bliss and HSA synergy score surfaces were selected as representative matrix-based interaction results. A synergy score greater than 10 was interpreted as a positive synergistic interaction trend, whereas scores between −10 and 10 were considered approximately additive.
